# Supplementary material for: Neonatal health care costs of very preterm babies in England: a retrospective analysis of a national birth cohort
Source: BMJ Paediatr Open. 2023 May 2;7(1):e001818. doi: 10.1136/bmjpo-2022-001818 (PMC10163543; doi:10.1136/bmjpo-2022-001818)
Supplement: Supplementary data [file bmjpo-2022-001818supp001.pdf]

Supplementary file

Multiple Imputation Methods

An imputation model was constructed, that included complete data on baseline baby and maternal characteristics, and the total cost of care provided at each level of care (intensive care, high-dependency care, special care without carer, special care with carer and normal care). We imputed costs for each level of care for babies with missing data (see Figure A1 below). Baseline characteristics included gestational age at birth, gender, number of fetuses, birthweight, neonatal death, maternal age and mode of delivery. All baseline covariates were subject to a small number of missing data that was imputed using conditional mean imputation prior to inclusion in the imputation model. A predictive mean matching estimation using chained equations with 50 imputations was implemented [1]. Mean estimates and estimates of standard errors were combined between imputed datasets using Rubin's rule [2] without any adjustment. Combined mean cost estimates and adjusted standard errors (SE) by gestational week across imputed datasets are shown to report the results of the multiple imputation analysis (Table A5 below).

Figure A1: Flow of NNRD data available to estimate neonatal care costs for the very preterm babies discharged from neonatal units in England between 2014-2018.

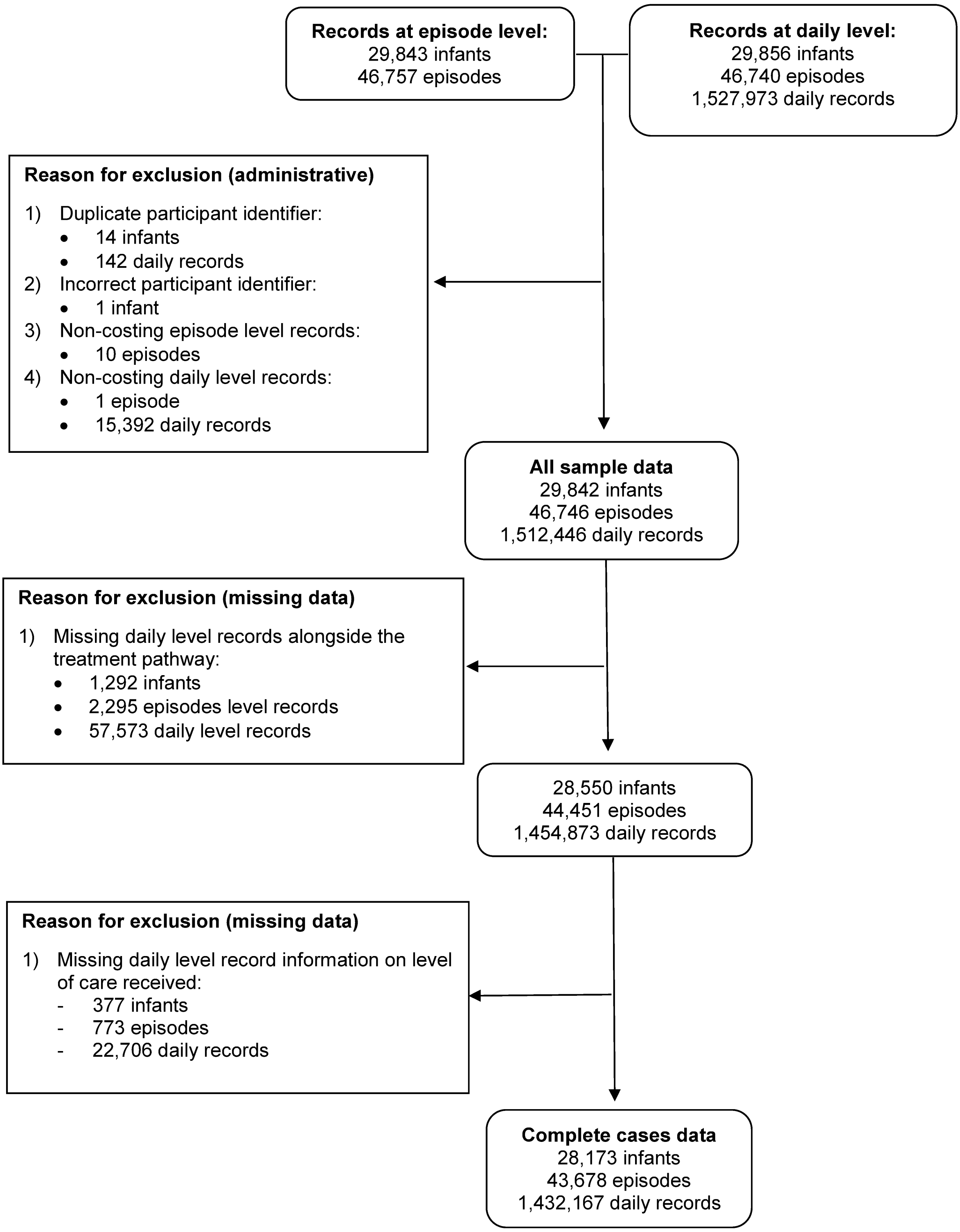

Table A1: Unit costs (expressed in 2018/19 UK pound sterling) used within the cost analysis

Resource Use Item	Unit cost 2018/19 UK £	Source
Neonatal unit inpatient bed days		
- intensive care level	£1,531	National Cost Collection Data Publication. National Schedule of NHS Costs 2018/19. HRG code XA01Z [3]
- high dependency care level	£1,007	National Cost Collection Data Publication. National Schedule of NHS Costs 2018/19. HRG code XA02Z [3]
- special care level, (carer not resident alongside baby)	£661	National Cost Collection Data Publication. National Schedule of NHS Costs 2018/19. HRG code XA03Z [3]
- special care level, (carer resident at cot-side and caring for baby)	£493	National Cost Collection Data Publication. National Schedule of NHS Costs 2018/19. HRG code XA04Z [3]
- normal care level	£514	National Cost Collection Data Publication. National Schedule of NHS Costs 2018/19. HRG code XA05Z [3]
Neonatal Critical Care, Transportation	£1,257	National Cost Collection Data Publication. National Schedule of NHS Costs 2018/19. HRG code XA06Z [3]
Inhaled nitric oxide (iNO) (per day)	£267	European iNO Registry for Liverpool Women's NHS Foundation Trust
*Surfactant replacement – poractant alfa (per day, by birth weight)**		
- Birth weight ≤0.6kg (1 bottle 1.5ml)	£282	British National Formulary online: NHS indicative price [4]
- 0.7kg≤ Birth weight ≤1.2kg (1 bottle 3ml)	£547	
- 1.3kg≤ Birth weight ≤1.8kg (1 bottle 3ml and 1 bottle 1.5ml)	£829	
- 1.9kg≤ Birth weight ≤2.4kg (2 bottles 3ml)	£1,095	
- 2.5kg≤ Birth weight ≤3kg (2 bottles 3ml and 1 bottle 1.5ml)	£1,376	
- 3.1kg≤ Birth weight ≤3.6kg (3 bottles 3ml)	£1,642	
- 3.7kg ≤ Birth weight≤4.2kg (3 bottles 3ml and 1 bottle 1.5ml)	£1,924	
Total parental nutrition (per day, over 14 days of use)**	£48	Walter et al. (2012); inflated to 2018/19 prices [5]
Palivizumab (per day at 15ml/kg birth weight)	£435	British National Formulary online: NHS indicative price [4]
ROP surgery	£1,731	National Cost Collection Data Publication. National Schedule of NHS Costs 2018/19. HRG code BZ86C, elective care [3]
Neonatal surgery***	Various	HRG4+ 2017/18 Reference Costs Grouper; inflated to 2018/19 prices [6]

ROP: retinopathy of prematurity;
*Surfactant use was reported from 3 separate fields from the datasets: Surfactant given today, Drugs given today and Surfactant given at resuscitation. The first two variables were recorded at the daily level, while the third variable was recorded at the episode level and therefore we defined its use for the first daily record of this episode;
**The use of total parental nutrition (TPN) was reported from 2 separate fields from the datasets: TPN given today and Drugs given today;
***Different types of neonatal surgery were identified from OPCS and ICD-10 codes using the HRG4+ 2017/18 Reference Costs Grouper Software.

Table A2: Baseline characteristics of babies born between 27 and 31 weeks gestation with and without missing NNRD data on daily records or level of daily care provided – data are frequencies (percentages) unless otherwise stated

	Babies with missing daily records or level of care data (n=1,669)	Babies with complete information (n=28,173)	p value*
	n (%)	n (%)	
**Gestational age at birth**			
27 weeks	284 (17.0%)	3,296 (11.7%)	
28 weeks	304 (18.2%)	4,370 (15.5%)	
29 weeks	289 (17.3%)	5,036 (17.9%)	p<0.001
30 weeks	354 (21.2%)	6,625 (23.5%)	
31 weeks	435 (26.1%)	8,827 (31.3%)	
Missing	*3 (0.2%)*	*19 (0.1%)*	
**Gender of baby**			
Male	961 (57.6%)	15,363 (54.5%)	0.046
Female	704 (42.2%)	12,755 (45.3%)	
Missing	*4 (0.2%)*	*55 (0.2%)*	
**Number of fetus**			
Singleton birth	1,229 (73.6%)	20,555 (73.0%)	0.632
Multiple birth	438 (26.2%)	7,598 (27.0%)	
Missing	*2 (0.1%)*	*20 (0.1%)*	
**Birthweight (g) - mean(SD)**	1,282.6 (358.2)	1,330.2 (332.2)	p<0.001
Missing	*8 (0.5%)*	*82 (0.3%)*	
**Apgar score at 5min - mean(SD)**	8.1 (1.7)	8.1 (1.8)	0.030
Missing	*156 (9.4%)*	*2,877 (10.2%)*	
**Died in neonatal care**	22 (1.3%)	985 (3.5%)	p<0.001
Missing	*1 (0.1%)*	*0 (0.0%)*	

SD: standard deviation; *Continuous variables were tested by independent t-test, categorical variables by chi-square test

Yang M, *et al. BMJ Paediatrics Open* 2023; 7:e001818. doi: 10.1136/bmjpo-2022-001818

Table A3: A comparison of gestational age at birth between very preterm births in the study cohort and those identified from national live births statistics for England for in 2016, 2017 and 2018

Year<br>Gestational weeks	Study cohort	National data for England from Office for National Statistics (ONS)	p-value*
**2016**			
27	704 (12.6%)	768 (12.6%)	
28	883 (15.8%)	950 (15.5%)	
29	1,015 (18.2%)	1,101 (18.0%)	0.977
30	1,282 (23.0%)	1,412 (23.1%)	
31	1,689 (30.3%)	1,881 (30.8%)	
**2017**			
27	638 (11.7%)	690 (11.6%)	
28	860 (15.7%)	949 (16.0%)	
29	991 (18.1%)	1,078 (18.2%)	0.987
30	1,305 (23.9%)	1,427 (24.1%)	
31	1,670 (30.6%)	1,787 (30.1%)	
**2018**			
27	476 (11.0%)	696 (12.0%)	
28	620 (14.3%)	888 (15.3%)	
29	760 (17.5%)	1,039 (17.9%)	0.151
30	1,035 (23.8%)	1,358 (23.4%)	
31	1,448 (33.3%)	1,835 (31.6%)	

*chi-square test of proportions
Source: Office for National Statistics; births extracted from a dataset containing birth registrations. 2016 and 2017 figures exclude births where mothers' usual residence was outside of England. 2018 figures include births where mothers' usual residence was in England and Wales. All figures were based on babies born in the calendar year.

Table A4: Counts of hospital transfers, surgeries and days receiving other non-routine procedures during neonatal admissions for very preterm babies in England for the period 2014-2018 (n = 28,154)*

	27 weeks gestation n=3,296	28 weeks gestation n=4,370	29 weeks gestation n=5,036	30 weeks gestation n=6,625	31 weeks gestation n=8,827
**Hospital transfer within 24 hours of birth**					
Number of transfers	287	330	293	286	311
Number (%) of babies receiving	276 (8.4%)	323 (7.4%)	286 (5.7%)	282 (4.3%)	307 (3.5%)
**Hospital transfer after 24 hours of birth**					
Number of transfers	872	836	713	659	571
Number (%) of babies receiving	654 (19.8%)	665 (15.2%)	593 (11.8%)	588 (8.9%)	523 (5.9%)
**Nitric oxide**					
Number of days of care	1,333	1,363	953	918	660
Number (%) of babies receiving	425 (12.9%)	467 (10.7%)	360 (7.1%)	419 (6.3%)	326 (3.7%)
**Surfactant replacement**					
Number of times given	5,932	6,561	5,525	4,201	3,667
Number (%) of babies receiving	2,743 (83.2%)	3,258 (74.6%)	3,023 (60.0%)	2,677 (40.4%)	2,386 (27.0%)
**TPN**					
Number of days of care (over 14 days' use)	20,182	17,919	11,200	7,525	5,074
Number (%) of babies receiving	1,391 (42.2%)	1,411 (32.3%)	990 (19.7%)	692 (10.4%)	515 (5.8%)
**Palivizumab**					
Number of times given	90	103	52	31	14
Number (%) of babies receiving	77 (2.3%)	68 (1.6%)	38 (0.8%)	26 (0.4%)	12 (0.1%)
**ROP surgery**					
Number of surgeries	116	53	36	34	15
Number (%) of babies receiving	91 (2.8%)	45 (1.0%)	32 (0.6%)	30 (0.5%)	15 (0.2%)
**Neonatal surgery**					
Number of surgeries	291	283	203	175	169
Number (%) of babies receiving	255 (7.7%)	266 (6.1%)	185 (3.7%)	162 (2.4%)	161 (1.8%)

TPN: total parenteral nutrition, ROP: retinopathy or prematurity.*19 babies had missing gestational age information.

Table A5: Mean (SE) level of care cost per baby (2018/19 UK £) for very preterm babies in England over the period 2014-2018 using multiple imputation (n = 29,842)

	**27 weeks gestation (n = 3,580)**	**28 weeks gestation (n = 4,674)**	**29 weeks gestation (n = 5,325)**	**30 weeks gestation (n = 6,979)**	**31 weeks gestation (n = 9,284)**
**Resource use item**	**Mean (SE) cost per baby**	**Mean (SE) cost per baby**	**Mean (SE) cost per baby**	**Mean (SE) cost per baby**	**Mean (SE) cost per baby**
**Level of daily care**					
Intensive care	£27,845 (£436)	£20,883 (£309)	£14,512 (£217)	£8,445 (£133)	£5,097 (£108)
High-dependency care	£27,212 (£386)	£19,284 (£292)	£11,486 (£198)	£7,788 (£135)	£5,112 (£82)
Special care without carer	£20,254 (£201)	£21,506 (£164)	£21,712 (£127)	£19,883 (£98)	£16,434 (£75)
Special care with carer	£762 (£22)	£829 (£22)	£825 (£20)	£850 (£18)	£832 (£15)
Normal care	£0 (£0)	£0.44 (£0.44)	£0 (£0)	£0.24 (£0.24)	£0.52 (£0.42)
**Total level of care costs**	**£76,072 (£613)**	**£62,502 (£461)**	**£48,534 (£326)**	**£36,966 (£222)**	**£27,475 (£159)**

SE: standard error

Yang M, *et al. BMJ Paediatrics Open* 2023; 7:e001818. doi: 10.1136/bmjpo-2022-001818

References

1. White, I.R., Royston, P., and Wood, A.M. Multiple imputation using chained equations: Issues and guidance for practice. Stat Med, 2011. **30**(4): 377-99.

2. Little, R.J. and Rubin, D.B. Statistical Analysis with Missing Data. 2nd ed. Wiley Series in Probability and Statistics. 2002, Hoboken, NJ: Wiley.

3. NHS England. (2019). National Cost Collection Data Publication. National Schedule of NHS Costs 2018/2019. Available from https://www.england.nhs.uk/publication/2018-19-national-cost-collection-data-publication/. [Accessed 12th November 2022].

4. Joint Formulary Committee. British National Formulary (online). Available from http://www.medicinescomplete.com. [Accessed 12th November 2022].

5. Walter, E., Liu, F.X., Maton, P., Storme, T., Perrinet, M., von Delft, O., Puntis, J., Hartigan, D., Dragosits, A., and Sondhi, S. Cost analysis of neonatal and pediatric parenteral nutrition in Europe: a multi-country study. Eur J Clin Nutr, 2012. **66**(5): 639-44.

6. NHS Digital. HRG4 2017/18 Local Payment Grouper. Available from https://digital.nhs.uk/services/national-casemix-office/downloads-groupers-and-tools/payment-hrg4-2017-18-local-payment-grouper. [Accessed 12 November 2022].

List of participating members of the UK Neonatal Collaborative in the OPTI-PREM Study

Institution	Neonatal Network	Clinical Lead
Airedale General Hospital	Yorkshire Neonatal Network	Dr Matthew Babirecki
Barnet Hospital	London - North Central Neonatal Network	Dr Tim Wickham
Barnsley District General Hospital	North Trent Neonatal Network	Dr Sanaa Hamdan
Basildon Hospital	London - North East and North Middlesex Neonatal Network	Dr Aashish Gupta
Basingstoke & North Hampshire Hospital	Thames Valley & Wessex Neonatal Networks	Dr Ruth Wigfield
City Hospital, Birmingham	Midlands South West Newborn Network	Dr Julie Nycyk
Broomfield Hospital	Norfolk, Suffolk & Cambridgeshire Neonatal Network	Dr Ahmed Hassan
Calderdale Royal Hospital	Yorkshire Neonatal Network	Dr Karin Schwarz
Chesterfield & North Derbyshire Royal Hospital	North Trent Neonatal Network	Dr Aiwyne Foo
Colchester General Hospital	Norfolk, Suffolk & Cambridgeshire Neonatal Network	Dr Aravind Shastri
Countess of Chester Hospital	Cheshire and Merseyside Neonatal Network	Dr Stephen Brearey
Croydon University Hospital	London - South West Neonatal Network	Dr John Chang
Diana Princess of Wales Hospital	North Trent Neonatal Network	Dr Pauline Adiotomre
Doncaster Royal Infirmary	North Trent Neonatal Network	Dr Jamal S Ahmed
Dorset County Hospital	Thames Valley & Wessex Neonatal Networks	Dr Abby Deketelaere
East Surrey Hospital	South East Coast Neonatal ODN	Dr K Abdul Khader
Great Western Hospital	South West Region	Dr Stanley Zengeya
Hillingdon Hospital	London - North West Neonatal Network	Dr Tristan Bate
Hinchingbrooke Hospital	Norfolk, Suffolk & Cambridgeshire Neonatal Network	Dr Hilary Dixon
Ipswich Hospital	Norfolk, Suffolk & Cambridgeshire Neonatal Network	Dr Matthew James
James Paget Hospital	Norfolk, Suffolk & Cambridgeshire Neonatal Network	Dr Ambadkar
Kettering General Hospital	Midlands Central Neonatal Network	Dr Patty Rao
King's Mill Hospital	Trent Perinatal Network	Dr Dhaval Dave
Kingston Hospital	London - South West Neonatal Network	Dr Vinay Pai
Leighton Hospital	Cheshire and Merseyside Neonatal Network	Dr Jayachandran
Lincoln County Hospital	Trent Perinatal Network	Dr Kollipara
Lister Hospital	Beds-Herts Neonatal Network	Dr J Kefas
Macclesfield District General Hospital	Cheshire and Merseyside Neonatal Network	Dr Gail Whitehead
Manor Hospital	Staffordshire, Shropshire & Black Country Newborn & Maternity Network	Dr Krishnamurthy
Milton Keynes Foundation Trust Hospital	Thames Valley & Wessex Neonatal Networks	Dr I Misra
Newham General Hospital	London - North East and North Middlesex Neonatal Network	Dr Imdad Ali
North Middlesex University Hospital	London - North East and North Middlesex Neonatal Network	Dr Lesley Alsford
North Tyneside General Hospital	Northern Neonatal Network	Vivien Spencer
Northampton General Hospital	Midlands Central Neonatal Network	Dr Subodh Gupta
Northwick Park Hospital	London - North West Neonatal Network	Dr Richard Nicholl
Ormskirk District General Hospital	Cheshire and Merseyside Neonatal Network	Dr Tim McBride
Peterborough City Hospital	Norfolk, Suffolk & Cambridgeshire Neonatal Network	Dr Katharine McDevitt
Pinderfields General Hospital	Yorkshire Neonatal Network	Dr David Gibson
Poole Hospital NHS Foundation Trust	Thames Valley & Wessex Neonatal Networks	Prof Minesh Khashu
Princess Alexandra Hospital	Norfolk, Suffolk & Cambridgeshire Neonatal Network	Dr Caitlin Toh
Queen Elizabeth Hospital, King's Lynn	Norfolk, Suffolk & Cambridgeshire Neonatal Network	Dr Glynis Rewitzky
Queen Elizabeth Hospital, Woolwich	London - South East Neonatal Network	Dr Olutoyin Banjoko
Queen's Hospital, Romford	London - North East and North Middlesex Neonatal Network	Dr Wilson Lopez

Rotherham District General Hospital	North Trent Neonatal Network	Dr Shameel Mattara
Royal Albert Edward Infirmary	Greater Manchester Neonatal Network	Dr Christos Zipitis
Royal Berkshire Hospital	Thames Valley & Wessex Neonatal Networks	Dr Peter De Halpert
Royal Cornwall Hospital	South West Region	Dr Paul Munyard
Royal Derby Hospital	Trent Perinatal Network	Dr John McIntyre
Royal Devon & Exeter Hospital	South West Region	Dr David Bartle
Royal Hampshire County Hospital	Thames Valley & Wessex Neonatal Networks	Dr Katie Yallop
Royal Lancaster Infirmary	Lancashire and South Cumbria Neonatal Network	Dr Joanne Fedee
Royal Oldham Hospital	Greater Manchester Neonatal Network	Dr Natasha Maddock
Royal Shrewsbury Hospital	Staffordshire, Shropshire & Black Country Newborn & Maternity Network	Dr Deshpande
Royal United Hospital	South West Region	Dr Stephen Jones
Russells Hall Hospital	Staffordshire, Shropshire & Black Country Newborn & Maternity Network	Dr Mahadevan
Salisbury District Hospital	Thames Valley & Wessex Neonatal Networks	Dr Nick Brown
Scunthorpe General Hospital	North Trent Neonatal Network	Dr Pauline Adiotomre
Southend Hospital	London - North East and North Middlesex Neonatal Network	Dr Arfa Khan
St Helier Hospital	London - South West Neonatal Network	Dr Salim Yasin
St Mary's Hospital, IOW	Thames Valley & Wessex Neonatal Networks	Dr Sian Butterworth
St Mary's Hospital, London	London - North West Neonatal Network	Dr Sunit Godambe
St Richard's Hospital	Thames Valley & Wessex Neonatal Networks	Dr Nick Brennan
Stepping Hill Hospital	Greater Manchester Neonatal Network	Dr Carrie Heal
Stoke Mandeville Hospital	Thames Valley & Wessex Neonatal Networks	Dr Sanjay Salgia
Tameside General Hospital	Greater Manchester Neonatal Network	Dr Jacqeline Birch
Taunton & Somerset Hospital	South West Region	Dr Chris Knight
Tunbridge Wells Hospital	South East Coast Neonatal ODN	Dr Hamudi Kisat
University Hospital Lewisham	London - South East Neonatal Network	Dr Jauro Kuna
University Hospital of North Durham	Northern Neonatal Network	Dr Mehdi Garbash
University Hospital of South Manchester	Greater Manchester Neonatal Network	Dr Gopi Vemuri
Victoria Hospital, Blackpool	Lancashire and South Cumbria Neonatal Network	Dr Chris Rawlingson
Warrington Hospital	Cheshire and Merseyside Neonatal Network	Dr Delyth Webb
Watford General Hospital	Beds-Herts Neonatal Network	Dr Sankara Narayanan
West Suffolk Hospital	Norfolk, Suffolk & Cambridgeshire Neonatal Network	Dr Ian Evans
Wexham Park Hospital	Thames Valley & Wessex Neonatal Networks	Dr Rekha Sanghavi
Whipps Cross University Hospital	London - North East and North Middlesex Neonatal Network	Dr Caroline Sullivan
Whiston Hospital	Cheshire and Merseyside Neonatal Network	Dr Rosaline Garr
Whittington Hospital	London - North Central Neonatal Network	Dr Wynne Leith
Worcestershire Royal Hospital	Midlands South West Newborn Network	Dr Andrew Gallagher
York District Hospital	Yorkshire Neonatal Network	Dr Guy Millman
Gloucestershire Royal Hospital	South West Region	Dr Simon Pirie
Arrowe Park Hospital	Cheshire and Merseyside Neonatal Network	Dr Anand Kamalanathan
Birmingham Heartlands Hospital	Midlands South West Newborn Network	Dr Phil Simmons
Birmingham Women's Hospital	Midlands South West Newborn Network	Dr Anju Singh
Bradford Royal Infirmary	Yorkshire Neonatal Network	Dr Sunita Seal
Chelsea & Westminster Hospital	London - North West Neonatal Network	Dr Mark Thomas
Derriford Hospital	South West Region	Dr Alex Allwood
Guy's & St Thomas' Hospital	London - South East Neonatal Network	Dr Timothy Watts
Homerton Hospital	London - North East and North Middlesex Neonatal Network	Dr Narendra Aladangady
hull royal infirmary	Yorkshire Neonatal Network	Dr Hassan Gaili
James Cook University Hospital	Northern Neonatal Network	Dr M Lal
King's College Hospital	London - South East Neonatal Network	Dr Ann Hickey
Lancashire Women & Newborn Centre	Lancashire and South Cumbria Neonatal Network	Dr Meera Lama
Leeds Neonatal Service	Yorkshire Neonatal Network	Dr Lawrence Miall
Leicester Neonatal Service	Midlands Central Neonatal Network	Dr Jonathan Cusack

Yang M, *et al. BMJ Paediatrics Open* 2023; 7:e001818. doi: 10.1136/bmjpo-2022-001818

Liverpool Women's Hospital	Cheshire and Merseyside Neonatal Network	Dr Bill Yoxall
Luton & Dunstable Hospital	Beds-Herts Neonatal Network	Dr Jennifer Birch
Medway Maritime Hospital	South East Coast Neonatal ODN	Dr Aung Soe
New Cross Hospital	Staffordshire, Shropshire & Black Country Newborn & Maternity Network	Dr Tilly Pillay
Norfolk & Norwich University Hospital	Norfolk, Suffolk & Cambridgeshire Neonatal Network	Dr Mark Dyke
Nottingham City Hospital	Trent Perinatal Network	Dr Steven Wardle
Nottingham University Hospital (QMC)	Trent Perinatal Network	Dr Steven Wardle
Oxford University Hospitals, John Radcliffe Hospital	Thames Valley & Wessex Neonatal Networks	Dr Eleri Adams
Princess Anne Hospital	Thames Valley & Wessex Neonatal Networks	Dr Mike Hall
Queen Alexandra Hospital	Thames Valley & Wessex Neonatal Networks	Dr Charlotte Groves
Queen Charlotte's Hospital	London - North West Neonatal Network	Dr Sunit Godambe
Rosie Maternity Hospital, Addenbrookes	Norfolk, Suffolk & Cambridgeshire Neonatal Network	Dr Angela D'Amore
Royal Bolton Hospital	Greater Manchester Neonatal Network	Dr Paul Settle
Royal Preston Hospital	Lancashire and South Cumbria Neonatal Network	Dr Richa Gupta
Royal Stoke University Hospital	Staffordshire, Shropshire & Black Country Newborn & Maternity Network	Dr Alison Moore
Royal Sussex County Hospital	South East Coast Neonatal ODN	Dr P Amess
Royal Victoria Infirmary	Northern Neonatal Network	Dr Alan Fenton
NORTH BRISTOL NHS TRUST (SOUTHMEAD)	South West Region	Dr Paul Mannix
St George's Hospital	London - South West Neonatal Network	Dr Charlotte Huddy
St Mary's Hospital, Manchester	Greater Manchester Neonatal Network	Dr Ngozi Edi-Osagie
St Michael's Hospital	South West Region	Dr Pamela Cairns
St Peter's Hospital	South East Coast Neonatal ODN	Dr Peter Reynolds
Sunderland Royal Hospital	Northern Neonatal Network	Dr Majd Abu-Harb
The Jessop Wing, Sheffield	North Trent Neonatal Network	Dr Simon Clark
The Royal London Hospital - Constance Green	London - North East and North Middlesex Neonatal Network	Dr Vadivelam Murthy
University College Hospital	London - North Central Neonatal Network	Dr Giles Kendall
University Hospital Coventry	Midlands Central Neonatal Network	Dr Kate Blake
University Hospital of North Tees	Northern Neonatal Network	Dr Hari Kumar
William Harvey Hospital	South East Coast Neonatal ODN	Dr Vimal Vasu

Yang M, *et al. BMJ Paediatrics Open* 2023; 7:e001818. doi: 10.1136/bmjpo-2022-001818
