# Supplementary material for: Neonatal health care costs of very preterm babies in England: a retrospective analysis of a national birth cohort
Source: BMJ Paediatr Open. 2023 May 2;7(1):e001818. doi: 10.1136/bmjpo-2022-001818 (PMC10163543; doi:10.1136/bmjpo-2022-001818)
Supplement: Author's manuscript [file bmjpo-2022-001818.draft_revisions.pdf]

BMJ Paediatrics Open

BMJ Paediatrics Open is committed to open peer review. As part of this commitment we make the peer review history of every article we publish publicly available.

When an article is published we post the peer reviewers' comments and the authors' responses online. We also post the versions of the paper that were used during peer review. These are the versions that the peer review comments apply to.

The versions of the paper that follow are the versions that were submitted during the peer review process. They are not the versions of record or the final published versions. They should not be cited or distributed as the published version of this manuscript.

BMJ Paediatrics Open is an open access journal and the full, final, typeset and author-corrected version of record of the manuscript is available on our site with no access controls, subscription charges or pay-per-view fees (http://bmjpaedsopen.bmj.com).

If you have any questions on BMJ Paediatrics Open's open peer review process please email info.bmjpo@bmj.com

BMJ Paediatrics Open

Neonatal health care costs of very preterm babies in England: a retrospective analysis of a national birth cohort

Journal:	*BMJ Paediatrics Open*
Manuscript ID	bmjpo-2022-001818
Article Type:	Original research
Date Submitted by the Author:	19-Dec-2022
Complete List of Authors:	Yang, Miaoqing; University of Oxford, Nuffield Department of Population Health<br>Campbell, Helen; University of Oxford, Nuffield Department of Population Health<br>Pillay, Thillagavathie; University Hospitals of Leicester NHS Trust, Neonatology; University of Wolverhampton Faculty of Science and Engineering, School of Medicine and Clinical Practice<br>Boyle, Elaine M.; University of Leicester, Health Sciences<br>Modi, Neena; Imperial College London, Neonatal Medicine<br>Rivero-Arias, Oliver; University of Oxford, Nuffield Department of Population Health
Keywords:	Health services research, Neonatology

SCHOLARONE™
Manuscripts

I, the Submitting Author has the right to grant and does grant on behalf of all authors of the Work (as defined in the below author licence), an exclusive licence and/or a non-exclusive licence for contributions from authors who are: i) UK Crown employees; ii) where BMJ has agreed a CC-BY licence shall apply, and/or iii) in accordance with the terms applicable for US Federal Government officers or employees acting as part of their official duties; on a worldwide, perpetual, irrevocable, royalty-free basis to BMJ Publishing Group Ltd ("BMJ") its licensees and where the relevant Journal is co-owned by BMJ to the co-owners of the Journal, to publish the Work in this journal and any other BMJ products and to exploit all rights, as set out in our licence.

The Submitting Author accepts and understands that any supply made under these terms is made by BMJ to the Submitting Author unless you are acting as an employee on behalf of your employer or a postgraduate student of an affiliated institution which is paying any applicable article publishing charge ("APC") for Open Access articles. Where the Submitting Author wishes to make the Work available on an Open Access basis (and intends to pay the relevant APC), the terms of reuse of such Open Access shall be governed by a Creative Commons licence – details of these licences and which Creative Commons licence will apply to this Work are set out in our licence referred to above.

Other than as permitted in any relevant BMJ Author's Self Archiving Policies, I confirm this Work has not been accepted for publication elsewhere, is not being considered for publication elsewhere and does not duplicate material already published. I confirm all authors consent to publication of this Work and authorise the granting of this licence.

Confidential: For Review Only

Neonatal health care costs of very preterm babies in England: a retrospective analysis of a national birth cohort

Miaoqing Yang[1,2], Helen Campbell[1], Thillagavathie Pillay[3], Elaine Boyle[4], Neena Modi[5], Oliver Rivero-Arias[1]

Running title: Neonatal costs of very preterm babies in England

[1] National Perinatal Epidemiology Unit (NPEU), Nuffield Department of Population Health, University of Oxford, Oxford, UK.
[2] Centre for Guidelines, National Institute for Health and Care Excellence, London, UK.
[3] Faculty of Science and Engineering, University of Wolverhampton, Wolverhampton, UK.
[4] Department of Population Health Sciences, University of Leicester, Leicester, UK.
[5] Section of Neonatal Medicine, School of Public Health, Chelsea and Westminster Hospital campus, Imperial College London, London, UK

Corresponding author
Associate Professor Oliver Rivero-Arias
National Perinatal Epidemiology Unit (NPEU)
Nuffield Department of Population Health
Old Road Campus
University of Oxford
OX3 7LF
Email: oliver.rivero@npeu.ox.ac.uk

Confidential: For Review Only

Abstract (244 words)

Objectives: Babies born between 27^{+0} and 31^{+6} weeks of gestation represent the largest group of very preterm babies requiring NHS care, however up-to-date cost figures for the UK are not currently available. This study estimates neonatal costs to hospital discharge for this group of very preterm babies in England.

Design: Retrospective analysis of resource use data recorded within the National Neonatal Research Database (NNRD).

Setting: Neonatal units in England.

Patients: Babies born between 27^{+0} and 31^{+6} weeks of gestation in England and discharged from a neonatal unit between 2014 and 2018.

Main outcome measures: Days receiving different levels of neonatal care were costed, along with other specialised clinical activities. Mean resource use and costs per baby are presented by gestational age at birth, along with total costs for the cohort.

Results: Based upon data for 28,173 very preterm babies, the annual total costs of neonatal care were estimated to be £262 million, with 95% of costs attributable to routine daily care provided by units. The mean (SD) cost per baby of daily care varied by gestational age at birth; £76,714 (£35,281) at 27 weeks as compared with £27,824 (£15,164) at 31 weeks.

Conclusions: Neonatal healthcare costs for very preterm babies vary substantially by gestational age at birth. As the first and largest study to estimate the costs of very preterm care in England using NNRD data, the findings presented here are a useful resource to stakeholders including NHS managers, clinicians, researchers, and policy makers.

Confidential: For Review Only

What is already known on this topic

Existing cost estimates for very preterm care in England are now over a decade old and may not reflect modern care practices.

What this study adds

To our knowledge this is the largest study to utilise individual patient-level data from a national research database to estimate neonatal healthcare costs for very preterm babies in England. The study has generated current cost estimates at the level of the individual baby and for the cohort as a whole and confirmed the previously reported inverse relationship between healthcare costs and gestational age at birth.

How this study might affect research, practice or policy

The outputs from this work provide a valuable resource for research assessing the economic implications of interventions to prevent preterm birth, the provision of care for preterm babies, as well as helping inform NHS resource allocation decisions.

Confidential: For Review Only

Introduction

Babies born between 27^{+0} and 31^{+6} weeks of gestation (hereafter called ‘born at 27–31 weeks’) represent the largest group of very preterm babies requiring NHS care.[1] These babies also account for about 12% of all viable preterm babies born in England and usually require admission to a neonatal unit.[1]

Previous work in the UK and elsewhere has attempted to estimate the healthcare and societal cost of preterm birth.[2-6] However, there is marked variability in reported cost estimates, due to differences in study perspectives, included babies, data sources and methods used to assign costs. A number of studies have reported cost estimates associated with the initial period of hospitalisation for babies born at 27-31 weeks.[2 3 6] Whilst these studies have shown the costs of neonatal care for very preterm babies to be inversely related to gestational age at birth, estimates for the UK are now over a decade old and there is a need for new analyses. As part of the OPTI-PREM suite of studies aimed at optimising neonatal service provision for very preterm babies in England, we conducted a retrospective cohort study to describe the levels of neonatal care, key specialist procedures, and healthcare costs attributable to the management of these babies.[1] The analysis makes use of healthcare resource use data routinely collated within the National Neonatal Research Database (NNRD) on babies admitted to all neonatal units in England, and so is, to the best of our knowledge, the largest participant-level study on the neonatal costs of very preterm babies in England.

Methods

Study design and population

For this retrospective analysis, we included all admissions to neonatal units in England for babies born at 27-31 weeks and discharged or died between January 2014 and December 2018. Admissions were identifiable through the NNRD, which was created in 2007 to support activities including audit, evaluations, and clinical, health services and policy research, and is maintained at the Neonatal Data Analysis Unit (NDAU) at Imperial College London [https://www.imperial.ac.uk/neonatal-data-analysis-unit/]. All NHS neonatal units in England, Scotland, Wales, and the Isle of Man use their Electronic

Confidential: For Review Only

Patient Record (EPR) supplier systems to routinely submit detailed information to the NNRD on the clinical care they provide to babies. Submitted data are quality-assured and curated to a research standard. Data comprise demographics, diagnoses, health outcomes, and daily interventions (including the level of care namely intensive, high dependency, special, or normal, provided each day) and treatments administered during the inpatient episode.

Data were extracted from the NNRD on 5th October 2020. Daily intervention data and treatment episode data were received in two separate datasets and were linked by means of participant identification number. Data were imported into Stata software and manipulated such that for each baby, a daily intervention record (which included the level of care provided) was available for each day of the admission episode. Babies missing daily intervention records or for whom daily records were available but level of care data had not been recorded, were excluded from the analysis.

Resource use and costs

The cost analysis was conducted from the perspective of the National Health Service (NHS) in England. To calculate the costs associated with the routine daily neonatal care received by each baby, we multiplied the number of days spent receiving each level of care, by level-specific national average bed day costs sourced from the 2018/19 National Schedule of NHS Costs.[7] Each bed day cost is derived by costing the daily care items included in its corresponding neonatal critical care healthcare resource group (HRG). For this analysis the neonatal bed day costs used were: intensive care (HRG XA01Z), high dependency care (HRG XA02Z), special care without carer resident alongside baby (HRG XA03Z), special care with carer resident alongside baby (HRG XA04Z), and normal care (HRG XA05Z). Supplementary Table A1 shows these unit costs.

Not all major clinical activities are included in these neonatal critical care HRGs. After in-depth discussions with clinical experts, we identified five high cost non-routine procedures captured within the NNRD, that were considered to be important in this particular population. These were nitric oxide, surfactant replacement, total parental nutrition (TPN) (over 14 days of use), palivizumab use and surgical care. Types of neonatal surgery were identified from their corresponding OPCS and ICD-10 codes for costing purposes, and costs for other activities were obtained from various sources as detailed

https://mc.manuscriptcentral.com/bmjpo

Confidential: For Review Only

in supplementary Table A1. NNRD data for these five non-routine procedures were assumed to be complete, with no entry in the corresponding dataset fields taken as an indication that the procedure did not take place.

Statistical analysis

When describing neonatal and maternal characteristics, counts and proportions and means and standard deviations (SD) were used for categorical and continuous variables respectively. Analyses of resource use data and costs were performed by gestational age at birth. Means (SD) were used to summarise days provided at each level of care and counts were made of the numbers of babies for whom non-routine procedures were recorded, along with the number of cases (for example surgeries) or days (for example for nitric oxide) for which such treatment was given. Total costs were reported for the cohort as a whole and again by gestational age at birth. Analyses were conducted using Stata MP.[8]

Patient and Public Involvement

An OPTI-PREM parent panel of mothers and fathers of babies born at 27-31 weeks in England was established with support from the national charity BLISS. The parent panel engaged in the study design and review of the funding protocol, development of parent information leaflets and neonatal unit posters. They attended team and study steering committee meetings, provided input at national stakeholder discussions, and contributed with the interpretation of final results, and our dissemination strategy.

Results

Study population

Within the NNRD data extraction were 29,842 infants, with a total of 1,512,446 daily records and 46,746 episode records. Following the removal of infants with missing daily record information (n=1,292) and those with missing data on the level of daily care provided (n=377), a total of 28,173 babies (94% of the starting cohort of 29,842) remained and were included in the cost analysis (full details of the record matching process and data cleaning can be found in supplementary Figure A1). Table 1 summarises the characteristics of these babies and their mothers. A comparison between babies included in the analysis and those excluded due to missing data (n=1,669) was performed and showed the latter were

Confidential: For Review Only

more likely to have been born at earlier gestations (for example 17% versus 12% were born at 27 weeks; see supplementary Table A2). Despite this, birth statistics for the study cohort were still comparable to those of all very preterm babies born in England between 2016 to 2018 (data compiled by the Office for National Statistics; see supplementary Table A3).

Resource use

Table 2 shows the mean (SD) per baby duration spent receiving each level of daily care according to gestational age at birth. Data show a consistent inverse relationship between gestational age at birth and the intensity of daily care provided. Figure 1 plots the mean durations along with mean overall length of stay on the neonatal unit and illustrates the longer durations of higher intensity care (and indeed overall neonatal care) provided to babies born at earlier gestations. For example, babies born at 27 weeks spent on average, 18.09 days (SD=15.70 days) receiving intensive care and 26.77 days (SD=22 days) receiving high-dependency care, while babies born at 31 weeks received an average of 3.31 days (SD=6.68 days) of intensive care and 5.05 days (SD=7.51 days) of high-dependency care. Also of note is that across all gestational age at birth groups, days spent receiving special care without a carer present accounted for the largest proportion of days spent on the neonatal unit.

Supplementary Table A4 presents the number of babies who underwent hospital transfers and received key non-routine procedures, again by gestational age at birth. A clear trend for greater resource utilisation amongst infants born at earlier gestations can be seen.

Costs

The mean (SD) cost per baby for the various levels of care and non-routine procedures are shown by gestational age at birth in Table 3. As expected, and given the observations for resource use, costs can be seen to increase as gestational age at birth decreases. Mean (SD) routine daily care costs for a baby born at 27 weeks, for example, were, at £75,594 (£34,874), 2.8 times greater than costs for a baby born at 31 weeks (£27,401 (£14,974)). The final column in Table 3 shows that for the 2014-2018 cohort as a whole, just over 50% of total costs were attributable to the provision of intensive and high dependency care, with a further 42% coming from the provision of special care (without a carer). Further exploration of the composition of total costs in Figure 2 reveals variation by gestational age at birth.

Confidential: For Review Only

Almost 70% of total costs for babies born at 27 weeks were associated with the use of intensive- and high-dependency care, while the proportion decreased to only 36% for babies born at 31 weeks. The usage, and thus cost of special care (without a carer present) increases with increasing gestational age at birth. Across all gestational at birth groups, non-routine procedures accounted for only a very small proportion of total costs.

The overall total cost estimated for the cohort over the five years from 2014 to 2018 (£1.3billion in Table 3) suggests the annual costs of neonatal care for babies born between 27 and 31 gestational weeks and admitted to a neonatal unit in England to be around £262 million, with the provision of routine daily care on these units accounting for 96% (£252 million) of overall costs.

Discussion

Main findings

To the best of our knowledge, this is the first study to make use of individual participant data within a UK national database to estimate the costs of care for all very preterm babies born between 27^{+0} and 31^{+6} weeks of gestation admitted to neonatal units in England. With data on over 28,000 babies analysed, it is also one of the largest studies to investigate neonatal care costs at both an individual infant and a national level. Analyses at the level of the individual baby revealed the main cost drivers to be the daily neonatal care provided to support babies. Analyses by gestational age at birth provided further evidence of the previously reported inverse relationship between resource use and healthcare costs and the degree of prematurity, with care for a baby born at 27 weeks estimated to cost almost three times more than for a baby born at 31 weeks.[5] Our work also provides valuable information on the contribution of different resource components to overall costs and illustrates how the mix of intensive of care required by these babies varies with gestational age at birth.

A small number of studies have previously estimated neonatal care costs for preterm babies, one of the most recent being by Rios et al. in Canada[6] which also used individual patient level resource use data from a neonatal database (the Canadian Neonatal Network Database) and included around 8,000 babies born between 27 and 31 weeks gestation. A comparison between the costs estimated for the babies in the Rios et al. study and the costs presented here (currency conversion from Can $ to UK £

Confidential: For Review Only

made using Purchasing Power Parities) revealed the Canadian cost estimates to be consistently lower across all gestational age groups.[9] The most obvious explanation for these differences lies with the scope of the neonatal databases used by each study and the resulting implications for duration of neonatal unit stay. In general Rios et al. reported consistently lower mean lengths of stay for all gestional ages compared to our estimates due to their focus on intensive care units and the exclusion of the costs of care in lower dependency neonatal units. Thus the Rios et al. estimates do not reflect the true costs of healthcare provision for this group of babies. Comparisons with UK studies assessing the costs of neonatal care for very preterm babies are also challenging, with some studies now being over a decade old and other more recently published works focussing on preterm births at other gestations (i.e. moderate and late preterm births or extremely preterm births).[2 4 10 11] One reassuring finding is that the costs of neonatal care for very preterm births presented here fall between the costs reported for moderate / late preterm infants and for extremely preterm infants in the UK.

Strengths and limitations

This study makes a number of contributions to the published literature. Firstly, existing UK cost estimates for neonatal care following very preterm birth are over a decade old and so are unlikely to reflect current standards of practice.[2] This study provides up-to-date figures based on a large cohort of more than 28,000 babies who were discharged from or died within neonatal units across England between 2014 and 2018. Secondly, previous UK cost studies in the area have mainly relied on data synthesised from secondary sources to estimate costs. In utilising the NNRD, this study has been able to employ detailed and quality assured individual participant data derived from electronic patient records (EPR). Furthermore, the dataset offered a unique opportunity to capture near population-wide data (and thus costs) on day-to-day care provided across all neonatal units without imposing any additional burden on study participants. Thirdly, the richness of the dataset permitted us not only to cost routine daily care provided at differing levels of intensity, but also to consider the cost implications of a number of major non-routine procedures, which are not captured within the HRG codes for neonatal critical care. Finally, by generating up to date estimates of the mean resource use and costs of neonatal care for very preterm babies in England, this study provides valuable data of interest to a range of stakeholders, including NHS managers, clinicians providing care, researchers assessing the economic

https://mc.manuscriptcentral.com/bmjpo

Confidential: For Review Only

implications of therapies and interventions to prevent and treat preterm birth, and decision makers charged with implementing new policies and allocating resources.

A number of limitations must also be acknowledged. This study considered only healthcare costs associated with the initial period of hospitalisation, though the economic consequences of preterm birth extend over prolonged periods of time, in some cases over the lifetime of the individual.[2 11 12] Preterm birth can lead to additional healthcare as well as social care needs and special educational needs throughout childhood.[13 14] Further research is needed to adequately capture these wider costs accurately, including the economic costs to families while their preterm baby was hospitalised, and then throughout their lives. A further limitation is the exclusion from the analysis of babies with missing data on daily care provision, who were shown to have been born at earlier gestations than babies with complete data (supplementary Table A2). Whilst these babies accounted for only 6% of the initial NNRD cohort and data showed no differences in gestational age at birth between the babies with complete data and all very preterm births registered in England (supplementary Table A4), the inclusion of such babies would have nevertheless increased total cohort costs and the estimates of annual total costs for this patient group shown in Table 3 from £1,309,192,377 to £1,393,642,826 and the annual total cost from £262 million to £279 million.

Conclusion

This study has generated up-to-date estimates of the costs of providing neonatal care to very preterm babies in England and the first to use individual participant-level data from a database with national coverage. Resource use and costs vary by gestational age at birth. The outputs from this work can be used to inform clinical and budgetary service planning and ensure the efficient allocation of health care resources.

Confidential: For Review Only

References

1. Pillay T, Modi N, Rivero-Arias O, et al. Optimising neonatal service provision for preterm babies born between 27 and 31 weeks gestation in England (OPTI-PREM), using national data, qualitative research and economic analysis: a study protocol. *BMJ Open* 2019;9(8):e029421. doi: 10.1136/bmjopen-2019-029421 [published Online First: 20190822]
2. Mangham LJ, Petrou S, Doyle LW, et al. The cost of preterm birth throughout childhood in England and Wales. *Pediatrics* 2009;123(2):e312-27. doi: 10.1542/peds.2008-1827
3. Korvenranta E, Linna M, Rautava L, et al. Hospital costs and quality of life during 4 years after very preterm birth. *Arch Pediatr Adolesc Med* 2010;164(7):657-63. doi: 10.1001/archpediatrics.2010.99
4. Khan KA, Petrou S, Dritsaki M, et al. Economic costs associated with moderate and late preterm birth: a prospective population-based study. *BJOG* 2015;122(11):1495-505. doi: 10.1111/1471-0528.13515 [published Online First: 20150722]
5. Petrou S, Yiu HH, Kwon J. Economic consequences of preterm birth: a systematic review of the recent literature (2009-2017). *Arch Dis Child* 2019;104(5):456-65. doi: 10.1136/archdischild-2018-315778 [published Online First: 20181109]
6. Rios JD, Shah PS, Beltempo M, et al. Costs of Neonatal Intensive Care for Canadian Infants with Preterm Birth. *J Pediatr* 2021;229:161-67 e12. doi: 10.1016/j.jpeds.2020.09.045 [published Online First: 20200923]
7. NHS England. National Cost Collection Data Publication. National Schedule of NHS Costs 2018/2019 2019 [Available from: https://www.england.nhs.uk/publication/2018-19-national-cost-collection-data-publication/ accessed 12th November 2022.
8. Stata Statistical Software Release 15 [program]. College Station, TX: StataCorp LP, 2015.
9. Eurostat-OECD. Methodological Manual on Purchasing Power Parities. Luxembourg: Publications Office of the European Union: OECD 2012.
10. Hinchliffe SR, Seaton SE, Lambert PC, et al. Modelling time to death or discharge in neonatal care: an application of competing risks. *Paediatr Perinat Epidemiol* 2013;27(4):426-33. doi: 10.1111/ppe.12053 [published Online First: 20130415]
11. Kim SW, Andronis L, Seppanen AV, et al. Economic costs at age five associated with very preterm birth: multinational European cohort study. *Pediatr Res* 2022;92(3):700-11. doi: 10.1038/s41390-021-01769-z [published Online First: 20211112]
12. Ni Y, Mendonca M, Baumann N, et al. Social Functioning in Adults Born Very Preterm: Individual Participant Meta-analysis. *Pediatrics* 2021;148(5) doi: 10.1542/peds.2021-051986 [published Online First: 20211026]
13. Alterman N, Johnson S, Carson C, et al. Gestational age at birth and child special educational needs: a UK representative birth cohort study. *Arch Dis Child* 2021;106(9):842-48. doi: 10.1136/archdischild-2020-320213 [published Online First: 20210122]
14. Alterman N, Johnson S, Carson C, et al. Gestational age at birth and academic attainment in primary and secondary school in England: Evidence from a national cohort study. *PloS one* 2022;17(8):e0271952. doi: 10.1371/journal.pone.0271952 [published Online First: 20220817]

For peer review only

Data availability statement

The National Neonatal Research Database can be accessed by making a request to the Neonatal Data Analysis Unit at Imperial College London through the Health Data Research UK Gateway (https://web.www.healthdatagateway.org/search?search=NNRD&datasetSort=latest&tab=Datasets).

Ethics statements

Patient consent for publication

Not required.

Ethics approval

Research ethics approval for the OPTI-PREM programme of work was obtained through the national Integrated Research Application System (IRAS, reference number 212 304 and research ethics committee reference number 17/NE/0800; North East – Tyne and Wear South).

Funding

This work is supported by the National Institute for Health Research, Health Services and Delivery Research Stream, project number 15/70/104 CRN accrual was approved by the NIHR for the period (1 August 2017 to 31 August 2018).

Competing interests

Authors declare no conflict of interests.

Contributors

ORA developed the study concept and aims, and developed the study proposal with MY, TP, EB, and NM. MY conducted the statistical analyses under the supervision of ORA. MY wrote the first draft of the manuscript with ORA and HC. All other authors reviewed the draft and provided critical input. All authors approve the final version.

Acknowledgments

Confidential: For Review Only

To parents, patients and families participating in the OPTI-PREM study, and to the OPTI-PREM Parent Panel, for its support. The OPTI-PREM study is grateful to its NIHR Project Steering Committee: Andrew Ewer (chair), Stavros Petrou, Gillian Santorelli, Josie Anderson, David Loughton, Lisa Stanton, Karen Luyt, and to the National Neonatal Collaborative. We are indebted to all members of the UK Neonatal Collaborative that participated and made this study possible (see supplementary file for list of members).

The OPTI-PREM Study team: Elaine M Boyle, Neena Modi, Oliver Rivero-Arias, Bradley Manktelow, Sarah E Seaton, Natalie Armstrong, Miaoqing Yang, Abdul Qader T Ismail, Vasiliki Bountziouka, Caroline S Cupit, Alexis Paton, Victor L Banda, Elizabeth S Draper, Kelvin Dawson and Thillagavathie Pillay (Chief Investigator).

Confidential: For Review Only

Table 1: Baby and maternal characteristics of study cohort - data are frequencies (percentages) unless otherwise stated

	**Cohort (n = 28,173)**
	**n (%)**
Baby characteristics	
**Gestational age at birth**	
27 weeks	3,296 (11.70%)
28 weeks	4,370 (15.51%)
29 weeks	5,036 (17.88%)
30 weeks	6,625 (23.52%)
31 weeks	8,827 (31.33%)
Missing	*19 (0.07%)*
**Gender of baby**	
Male	15,363 (54.53%)
Female	12,755 (45.27%)
Missing	*55 (0.20%)*
**Number of fetuses**	
Singleton birth	20,555 (72.96%)
Multiple birth	7,598 (26.97%)
Missing	*20 (0.07%)*
**Birthweight (g) – mean (SD)**	1330.17 (332.18)
Missing	*82 (0.29%)*
**Apgar score at 5min – mean (SD)**	8.05 (1.76)
Missing	*2,877 (10.21%)*
**Died in neonatal care**	985 (3.50%)
Missing	*0 (0.00%)*
Maternal characteristics	
**Age (years) – mean (SD)**	30.79 (6.28)
Missing	*279 (0.99%)*
**Ethnicity**	
White	17,647 (62.64%)
Black	2,011 (7.14%)
Asian	3,144 (11.16%)
Mixed	433 (1.54%)
Other	483 (1.71%)
Missing	*4,455 (15.81%)*
**Diabetes**	2,378 (8.44%)
Missing	*10,736 (38.11%)*
**Hypertension**	3,506 (12.44%)
Missing	*10,460 (37.13%)*
**Infection**	3,029 (10.75%)
Missing	*10,595 (37.61%)*
**Mode of delivery**	
Vaginal birth	9,056 (32.14%)
Caesarean birth	17,865 (63.41%)
Missing	*1,252 (4.44%)*

Confidential: For Review Only

**Quintiles of IMD**	
1st Q, least deprived	3,355 (11.91%)
2nd Q	3,761 (13.35%)
3rd Q	4,516 (16.03%)
4th Q	5,930 (21.05%)
5th Q, most deprived	8,057 (28.60%)
Missing	*2,554 (9.07%)*

SD: standard deviation; IMD: Index of Multiple Deprivation; Q: quintile

Confidential: For Review Only

Table 2: Summary of type and duration of daily care received by very preterm babies during neonatal unit admissions in England for the period 2014-2018

	Level of care provided				
**Gestational age at birth**	**Intensive care (XA01Z)**	**High-dependency care (XA02Z)**	**Special care without carer (XA03Z)**	**Special care with carer (XA04Z)**	**Normal care (XA05Z)**
**27 weeks gestation (n=3,296)**					
Number of days of care	59,494	88,212	100,770	5,113	0
Number of babies receiving care	3,275	3,065	3,021	1,681	0
Mean duration of care (in days)*	18.09	26.77	30.54	1.54	0.00
SD	15.70	22.00	17.97	2.50	0.00
95% CI	(17.94 to 18.23)	(26.59 to 26.95)	(30.35 to 30.73)	(1.50 to 1.59)	(0.00 to 0.00)
Median duration of care (in days)*	14.00	23.00	30.50	1.00	0.00
Range	(0.00,174.00)	(0.00,243.00)	(0.00,161.00)	(0.00,31.00)	(0.00,0.00)
**28 weeks gestation (n=4,370)**					
Number of days of care	59,440	83,399	141,630	7,345	4
Number of babies receiving care	4,298	4,050	4,097	2,268	1
Mean duration of care (in days)*	13.63	19.09	32.39	1.68	0.00
SD	13.37	19.45	16.52	2.95	0.06
95% CI	(13.52 to 13.74)	(18.96 to 19.22)	(32.22 to 32.55)	(1.64 to 1.72)	(0.00 to 0.00)
Median duration of care (in days)*	11.00	14.00	33.00	1.00	0.00
Range	(0.00,246.00)	(0.00,203.00)	(0.00,127.00)	(0.00,44.00)	(0.00,4.00)
**29 weeks gestation (n=5,036)**					
Number of days of care	47,728	57,613	165,090	8,449	0
Number of babies receiving care	4,774	4,631	4,868	2,671	0
Mean duration of care (in days)*	9.50	11.44	32.77	1.67	0.00
SD	10.19	14.10	13.78	2.95	0.00
95% CI	(9.42 to 9.59)	(11.35 to 11.54)	(32.61 to 32.92)	(1.64 to 1.71)	(0.00 to 0.00)
Median duration of care (in days)*	8.00	7.00	33.00	1.00	0.00
Range	(0.00,258.00)	(0.00,196.00)	(0.00,119.00)	(0.00,45.00)	(0.00,0.00)
**30 weeks gestation (n=6,625)**					
Number of days of care	36,599	51,479	199,283	11,428	3
Number of babies receiving care	5,196	5,724	6,471	3,582	1
Mean duration of care (in days)*	5.54	7.77	30.07	1.72	0.00
SD	7.11	11.11	12.13	2.93	0.04
95% CI	(5.48 to 5.60)	(7.71 to 7.84)	(29.94 to 30.20)	(1.69 to 1.75)	(0.00 to 0.00)
Median duration of care (in days)*	4.00	5.00	30.00	1.00	0.00
Range	(0.00,133.00)	(0.00,213.00)	(0.00,115.00)	(0.00,37.00)	(0.00,3.00)
**31 weeks gestation (n=8,827)**					
Number of days of care	29,094	44,547	219,355	14,948	9
Number of babies receiving care	5,124	7,055	8,672	4,718	2
Mean duration of care (in days)*	3.31	5.05	24.85	1.69	0.00
SD	6.68	7.51	10.50	2.80	0.08
95% CI	(3.27 to 3.34)	(5.00 to 5.10)	(24.74 to 24.95)	(1.66 to 1.71)	(0.00 to 0.00)
Median duration of care (in days)*	1.00	3.00	24.00	1.00	0.00
Range	(0.00,201.00)	(0.00,154.00)	(0.00,240.00)	(0.00,36.00)	(0.00,7.00)

SD: standard deviation, CI: parametric confidence interval, *Estimated across all babies within a gestational age group

Confidential: For Review Only

Table 3: Mean (SD) per baby and total neonatal care costs (2018/19 UK £) for very preterm babies in England over the period 2014-2018

	27 weeks gestation	28 weeks gestation	29 weeks gestation	30 weeks gestation	31 weeks gestation	
**Resource use item**	**Mean (SD) cost per baby**	**Mean (SD) cost per baby**	**Mean (SD) cost per baby**	**Mean (SD) cost per baby**	**Mean (SD) cost per baby**	**Total neonatal care costs (percentage) for 2014-2018**
**Level of daily care**						
Intensive care	£27,690 (£24,039)	£20,865 (£20,473)	£14,548 (£15,603)	£8,482 (£10,887)	£5,062 (£10,235)	£356,747,496 (27.25%)
High-dependency care	£26,956 (£22,157)	£19,221 (£19,584)	£11,523 (£14,203)	£7,828 (£11,186)	£5,083 (£7,567)	£327,832,878 (25.04%)
Special care without carer	£20,187 (£11,880)	£21,407 (£10,921)	£21,659 (£9,110)	£19,875 (£8,019)	£16,423 (£6,939)	£546,285,433 (41.73%)
Special care with carer	£761 (£1,235)	£826 (£1,454)	£824 (£1,453)	£847 (£1,444)	£831 (£1,378)	£23,230,160 (1.77%)
Normal care	£0 (£0)	£0.47 (£31)	£0 (£0)	£0.23 (£19)	£0.52 (£40)	£8,224 (0.00%)
**Total level of care costs**	**£75,594 (£34,874)**	**£62,319 (£30,841)**	**£48,554 (£23,426)**	**£37,033 (£18,276)**	**£27,401 (£14,947)**	**£1,254,104,191 (95.79%)**
**Hospital transfers**	**£1,120 (£1,435)**	**£886 (£1,277)**	**£712 (£1,148)**	**£566 (£997)**	**£423 (£860)**	**£18,660,165 (1.43%)**
**Non-routine procedures**						
Nitric oxide	£105 (£557)	£81 (£436)	£49 (£354)	£36 (£312)	£19 (£148)	£1,356,929 (0.10%)
Surfactant replacement	£1,006 (£2,052)	£933 (£1,980)	£784 (£1,970)	£497 (£995)	£351 (£1,340)	£17,742,128 (1.36%)
TPN >14 days use	£930 (£2,203)	£609 (£1,838)	£316 (£1,398)	£147 (£883)	£71 (£550)	£8,919,898 (0.68%)
Palivizumab	£172 (£1,188)	£152 (£1,518)	£74 (£1,007)	£40 (£716)	£16 (£467)	£2,022,625 (0.15%)
ROP surgery	£61 (£398)	£21 (£223)	£12 (£165)	£8.9 (£138)	£2.9 (£71)	£439,674 (0.03%)
Neonatal surgery	£456 (£2,053)	£358 (£1,844)	£206 (£1,325)	£135 (£996)	£107 (£934)	£5,946,767 (0.45%)
**Total non-routine procedures costs**	**£2,730 (£4,594)**	**£2,153 (£4,228)**	**£1,442 (£3,333)**	**£864 (£2,143)**	**£567 (£1,955)**	**£36,428,021(2.78%)**
Total neonatal care costs for 2014-2018						***£1,309,192,377***
Annual total neonatal costs						***£261,838,475***

SD: standard deviation, NHS: National Health Service, TPN: total parenteral nutrition, ROP: retinopathy of prematurity

Confidential: For Review Only

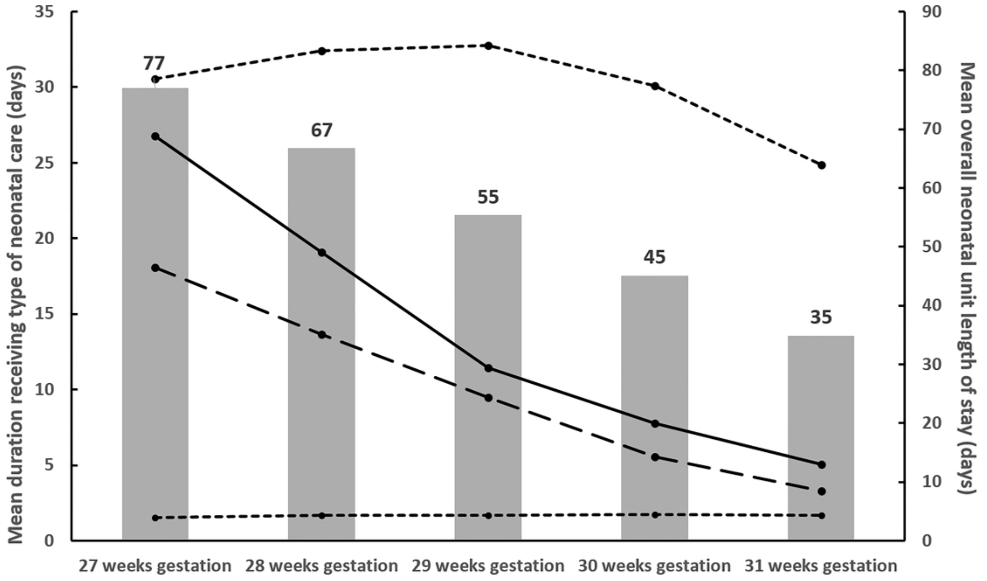

Figure 1 - Mean durations of different levels of daily care provided and overall length of stay per baby (in days) during neonatal unit admissions in England for the period 2014-2018. Data shown by gestational age at birth

463x275mm (300 x 300 DPI)

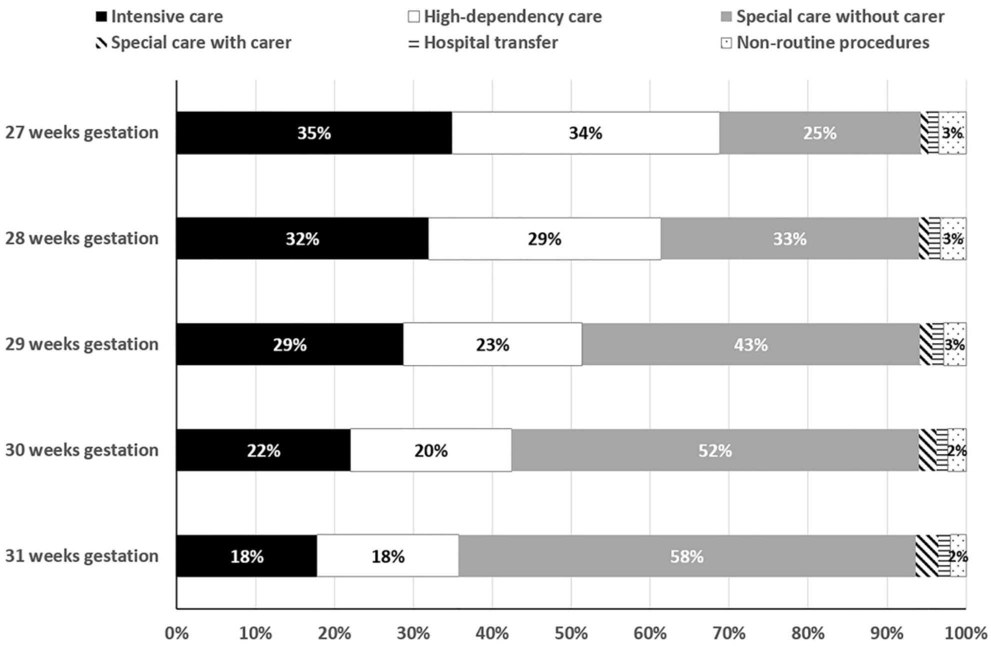

Figure 2 - Proportion of neonatal care cost attributable to different cost categories, by gestational age at birth

420x277mm (300 x 300 DPI)

Supplementary file

Table A1: Unit costs (expressed in 2018/19 UK pound sterling) used within the cost analysis

Resource Use Item	Unit cost 2018/19 UK £	Source
Neonatal unit inpatient bed days		
- intensive care level	£1,531	National Cost Collection Data Publication. National Schedule of NHS Costs 2018/19. HRG code XA01Z [1]
- high dependency care level	£1,007	National Cost Collection Data Publication. National Schedule of NHS Costs 2018/19. HRG code XA02Z [1]
- special care level, (carer not resident alongside baby)	£661	National Cost Collection Data Publication. National Schedule of NHS Costs 2018/19. HRG code XA03Z [1]
- special care level, (carer resident at cot-side and caring for baby)	£493	National Cost Collection Data Publication. National Schedule of NHS Costs 2018/19. HRG code XA04Z [1]
- normal care level	£514	National Cost Collection Data Publication. National Schedule of NHS Costs 2018/19. HRG code XA05Z [1]
Neonatal Critical Care, Transportation	£1,257	National Cost Collection Data Publication. National Schedule of NHS Costs 2018/19. HRG code XA06Z [1]
Inhaled nitric oxide (iNO) (per day)	£266.52	European iNO Registry for Liverpool Women's NHS Foundation Trust
*Surfactant replacement – poractant alfa (per day, by birth weight)**		
- Birth weight ≤0.6kg (1 bottle 1.5ml)	£281.61	British National Formulary online: NHS indicative price [2]
- 0.7kg≤ Birth weight ≤1.2kg (1 bottle 3ml)	£547.40	
- 1.3kg≤ Birth weight ≤1.8kg (1 bottle 3ml and 1 bottle 1.5ml)	£829.01	
- 1.9kg≤ Birth weight ≤2.4kg (2 bottles 3ml)	£1,094.8	
- 2.5kg≤ Birth weight ≤3kg (2 bottles 3ml and 1 bottle 1.5ml)	£1,376.41	
- 3.1kg≤ Birth weight ≤3.6kg (3 bottles 3ml)	£1,642.2	
- 3.7kg ≤ Birth weight≤4.2kg (3 bottles 3ml and 1 bottle 1.5ml)	£1,923.81	
Total parental nutrition (per day, over 14 days of use)**	£48.15	Walter et al. (2012); inflated to 2018/19 prices [3]
Palivizumab (per day at 15ml/kg birth weight)	£435	British National Formulary online: NHS indicative price [2]
ROP surgery	£1731	National Cost Collection Data Publication. National Schedule of NHS Costs 2018/19. HRG code BZ86C, elective care [1]

Neonatal surgery***	Various	HRG4+ 2017/18 Reference Costs Grouper; inflated to 2018/19 prices [4]

ROP: retinopathy of prematurity; *Surfactant use was reported from 3 separate fields from the datasets: Surfactant given today, Drugs given today and Surfactant given at resuscitation. The first two variables were recorded at the daily level, while the third variable was recorded at the episode level and therefore we defined its use for the first daily record of this episode; **The use of total parental nutrition (TPN) was reported from 2 separate fields from the datasets: TPN given today and Drugs given today; ***Different types of neonatal surgery were identified from OPCS and ICD-10 codes using the HRG4+ 2017/18 Reference Costs Grouper Software.

Confidential: For Review Only

Table A2: Baseline characteristics of babies born between 27 and 31 weeks gestation with and without missing NNRD data on daily records or level of daily care provided – data are frequencies (percentages) unless otherwise stated

	Babies with missing daily records or level of care data (n=1,669)	Babies with complete information (n=28,173)	p value*
	n (%)	n (%)	
**Gestational age at birth**			
27 weeks	284 (17.02%)	3,296 (11.70%)	
28 weeks	304 (18.21%)	4,370 (15.51%)	
29 weeks	289 (17.32%)	5,036 (17.88%)	0.000
30 weeks	354 (21.21%)	6,625 (23.52%)	
31 weeks	435 (26.06%)	8,827 (31.33%)	
Missing	*3 (0.18%)*	*19 (0.07%)*	
**Gender of baby**			
Male	961 (57.58%)	15,363 (54.53%)	0.046
Female	704 (42.18%)	12,755 (45.27%)	
Missing	*4 (0.24%)*	*55 (0.20%)*	
**Number of fetus**			
Singleton birth	1,229 (73.64%)	20,555 (72.96%)	0.632
Multiple birth	438 (26.24%)	7,598 (26.97%)	
Missing	*2 (0.12%)*	*20 (0.07%)*	
**Birthweight (g) - mean(SD)**	1282.64 (358.15)	1330.17 (332.18)	0.000
Missing	*8 (0.48%)*	*82 (0.29%)*	
**Apgar score at 5min - mean(SD)**	7.87 (1.85)	8.05 (1.76)	0.000
Missing	*156 (9.35%)*	*2,877 (10.21%)*	
**Died in neonatal care**	22 (1.32%)	985 (3.50%)	0.000
Missing	*1 (0.06%)*	*0 (0.00%)*	

SD: standard deviation; *Continuous variables were tested by independent t-test, categorical variables by chi-square test

Confidential: For Review Only

Table A3: A comparison of gestational age at birth between very preterm births in the study cohort and those identified from national live births statistics for England for in 2016, 2017 and 2018

Year<br>Gestational weeks	Study cohort	National data for England from Office for National Statistics (ONS)	p-value*
**2016**			
27	704 (12.63%)	768 (12.57%)	
28	883 (15.84%)	950 (15.54%)	
29	1,015 (18.21%)	1,101 (18.01%)	0.977
30	1,282 (23.00%)	1,412 (23.10%)	
31	1,689 (30.30%)	1,881 (30.78%)	
**2017**			
27	638 (11.68%)	690 (11.63%)	
28	860 (15.74%)	949 (16.00%)	
29	991 (18.14%)	1,078 (18.18%)	0.987
30	1,305 (23.88%)	1,427 (24.06%)	
31	1,670 (30.56%)	1,787 (30.13%)	
**2018**			
27	476 (10.96%)	696 (11.97%)	
28	620 (14.27%)	888 (15.27%)	
29	760 (17.50%)	1,039 (17.86%)	0.151
30	1,035 (23.83%)	1,358 (23.35%)	
31	1,448 (33.33%)	1,835 (31.55%)	

*chi-square test of proportions
Source: Office for National Statistics; births extracted from a dataset containing birth registrations. 2016 and 2017 figures exclude births where mothers' usual residence was outside of England. 2018 figures include births where mothers' usual residence was in England and Wales. All figures were based on babies born in the calendar year.

Confidential: For Review Only

Table A4: Counts of hospital transfers, surgeries and days receiving other non-routine procedures during neonatal admissions for very pre-term babies in England for the period 2014-2018

	27 weeks gestation n=3,296	28 weeks gestation n=4,370	29 weeks gestation n=5,036	30 weeks gestation n=6,625	31 weeks gestation n=8,827
**Hospital transfer within 24 hours of birth***					
Number of transfers	287	330	293	286	311
Number (%) of babies receiving	276 (8.4%)	323 (7.4%)	286 (5.7%)	282 (4.3%)	307 (3.5%)
**Hospital transfer after 24 hours of birth***					
Number of transfers	872	836	713	659	571
Number (%) of babies receiving	654 (19.8%)	665 (15.2%)	593 (11.8%)	588 (8.9%)	523 (5.9%)
**Nitric oxide**					
Number of days of care	1,333	1,363	953	918	660
Number (%) of babies receiving	425 (12.9%)	467 (10.7%)	360 (7.1%)	419 (6.3%)	326 (3.7%)
**Surfactant replacement**					
Number of days of care	5,932	6,561	5,525	4,201	3,667
Number (%) of babies receiving	2,743 (83.2%)	3,258 (74.6%)	3,023 (60.0%)	2,677 (40.4%)	2,386 (27.0%)
**TPN**					
Number of days of care (over 14 days' use)	20,182	17,919	11,200	7,525	5,074
Number (%) of babies receiving	1,391 (42.2%)	1,411 (32.3%)	990 (19.7%)	692 (10.4%)	515 (5.8%)
**Palivizumab**					
Number of days of care	90	103	52	31	14
Number (%) of babies receiving	77 (2.3%)	68 (1.6%)	38 (0.8%)	26 (0.4%)	12 (0.1%)
**ROP surgery**					
Number of days of care	116	53	36	34	15
Number (%) of babies receiving	91 (2.8%)	45 (1.0%)	32 (0.6%)	30 (0.5%)	15 (0.2%)
**Neonatal surgery***					
Number of surgeries	291	283	203	175	169
Number (%) of babies receiving	255 (7.7%)	266 (6.1%)	185 (3.7%)	162 (2.4%)	161 (1.8%)

TPN: total parenteral nutrition, ROP: retinopathy or prematurity,*For these items, numbers recorded are reported. For remaining items total number of days receiving treatment are reported.

Confidential: For Review Only

Figure A1: Flow of NNRD data available to estimate neonatal care costs for the very preterm babies discharged from neonatal units in England between 2014-2018.

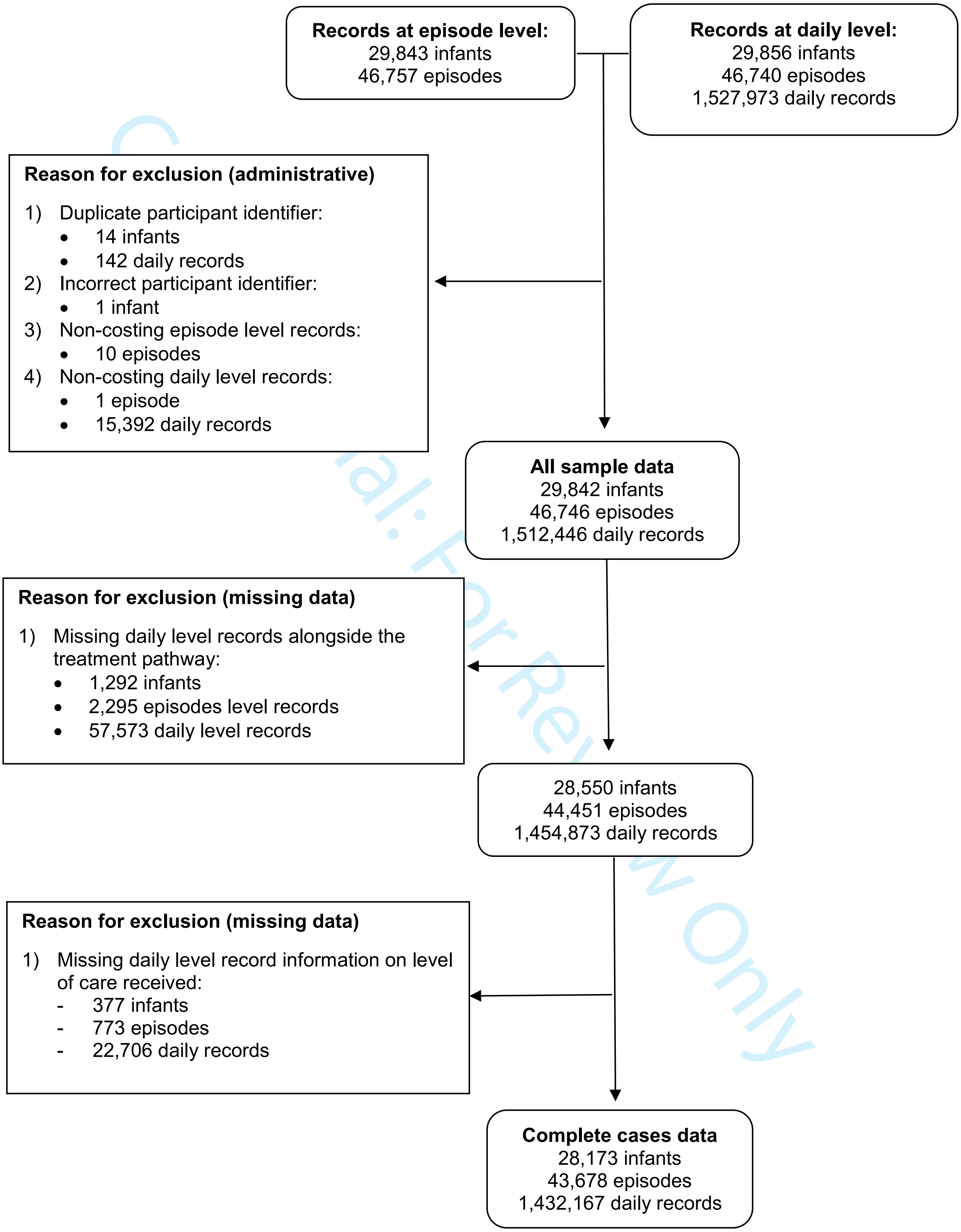

Confidential: For Review Only

References

1. NHS England. (2019). National Cost Collection Data Publication. National Schedule of NHS Costs 2018/2019. Available from https://www.england.nhs.uk/publication/2018-19-national-cost-collection-data-publication/. [Accessed 12th November 2022].

2. Joint Formulary Committee. British National Formulary (online). Available from http://www.medicinescomplete.com. [Accessed 12th November 2022].

3. Walter, E., Liu, F.X., Maton, P., Storme, T., Perrinet, M., von Delft, O., Puntis, J., Hartigan, D., Dragosits, A., and Sondhi, S. Cost analysis of neonatal and pediatric parenteral nutrition in Europe: a multi-country study. Eur J Clin Nutr, 2012. **66**(5): 639-44.

4. NHS Digital. HRG4 2017/18 Local Payment Grouper. Available from https://digital.nhs.uk/services/national-casemix-office/downloads-groupers-and-tools/payment-hrg4-2017-18-local-payment-grouper. [Accessed 12 November 2022].

Confidential: For Review Only

List of participating members of the UK Neonatal Collaborative in the OPTI-PREM Study

Institution	Neonatal Network	Clinical Lead
Airedale General Hospital	Yorkshire Neonatal Network	Dr Matthew Babirecki
Barnet Hospital	London - North Central Neonatal Network	Dr Tim Wickham
Barnsley District General Hospital	North Trent Neonatal Network	Dr Sanaa Hamdan
Basildon Hospital	London - North East and North Middlesex Neonatal Network	Dr Aashish Gupta
Basingstoke & North Hampshire Hospital	Thames Valley & Wessex Neonatal Networks	Dr Ruth Wigfield
City Hospital, Birmingham	Midlands South West Newborn Network	Dr Julie Nycyk
Broomfield Hospital	Norfolk, Suffolk & Cambridgeshire Neonatal Network	Dr Ahmed Hassan
Calderdale Royal Hospital	Yorkshire Neonatal Network	Dr Karin Schwarz
Chesterfield & North Derbyshire Royal Hospital	North Trent Neonatal Network	Dr Aiwyne Foo
Colchester General Hospital	Norfolk, Suffolk & Cambridgeshire Neonatal Network	Dr Aravind Shastri
Countess of Chester Hospital	Cheshire and Merseyside Neonatal Network	Dr Stephen Brearey
Croydon University Hospital	London - South West Neonatal Network	Dr John Chang
Diana Princess of Wales Hospital	North Trent Neonatal Network	Dr Pauline Adiotomre
Doncaster Royal Infirmary	North Trent Neonatal Network	Dr Jamal S Ahmed
Dorset County Hospital	Thames Valley & Wessex Neonatal Networks	Dr Abby Deketelaere
East Surrey Hospital	South East Coast Neonatal ODN	Dr K Abdul Khader
Great Western Hospital	South West Region	Dr Stanley Zengeya
Hillingdon Hospital	London - North West Neonatal Network	Dr Tristan Bate
Hinchingbrooke Hospital	Norfolk, Suffolk & Cambridgeshire Neonatal Network	Dr Hilary Dixon
Ipswich Hospital	Norfolk, Suffolk & Cambridgeshire Neonatal Network	Dr Matthew James
James Paget Hospital	Norfolk, Suffolk & Cambridgeshire Neonatal Network	Dr Ambadkar
Kettering General Hospital	Midlands Central Neonatal Network	Dr Patty Rao
King's Mill Hospital	Trent Perinatal Network	Dr Dhaval Dave
Kingston Hospital	London - South West Neonatal Network	Dr Vinay Pai
Leighton Hospital	Cheshire and Merseyside Neonatal Network	Dr Jayachandran
Lincoln County Hospital	Trent Perinatal Network	Dr Kollipara
Lister Hospital	Beds-Herts Neonatal Network	Dr J Kefas
Macclesfield District General Hospital	Cheshire and Merseyside Neonatal Network	Dr Gail Whitehead
Manor Hospital	Staffordshire, Shropshire & Black Country Newborn & Maternity Network	Dr Krishnamurthy
Milton Keynes Foundation Trust Hospital	Thames Valley & Wessex Neonatal Networks	Dr I Misra
Newham General Hospital	London - North East and North Middlesex Neonatal Network	Dr Imdad Ali
North Middlesex University Hospital	London - North East and North Middlesex Neonatal Network	Dr Lesley Alsford
North Tyneside General Hospital	Northern Neonatal Network	Vivien Spencer
Northampton General Hospital	Midlands Central Neonatal Network	Dr Subodh Gupta
Northwick Park Hospital	London - North West Neonatal Network	Dr Richard Nicholl
Ormskirk District General Hospital	Cheshire and Merseyside Neonatal Network	Dr Tim McBride
Peterborough City Hospital	Norfolk, Suffolk & Cambridgeshire Neonatal Network	Dr Katharine McDevitt
Pinderfields General Hospital	Yorkshire Neonatal Network	Dr David Gibson
Poole Hospital NHS Foundation Trust	Thames Valley & Wessex Neonatal Networks	Prof Minesh Khashu
Princess Alexandra Hospital	Norfolk, Suffolk & Cambridgeshire Neonatal Network	Dr Caitlin Toh
Queen Elizabeth Hospital, King's Lynn	Norfolk, Suffolk & Cambridgeshire Neonatal Network	Dr Glynis Rewitzky
Queen Elizabeth Hospital, Woolwich	London - South East Neonatal Network	Dr Olutoyin Banjoko
Queen's Hospital, Romford	London - North East and North Middlesex Neonatal Network	Dr Wilson Lopez
Rotherham District General Hospital	North Trent Neonatal Network	Dr Shameel Mattara
Royal Albert Edward Infirmary	Greater Manchester Neonatal Network	Dr Christos Zipitis
Royal Berkshire Hospital	Thames Valley & Wessex Neonatal Networks	Dr Peter De Halpert
Royal Cornwall Hospital	South West Region	Dr Paul Munyard
Royal Derby Hospital	Trent Perinatal Network	Dr John McIntyre
Royal Devon & Exeter Hospital	South West Region	Dr David Bartle
Royal Hampshire County Hospital	Thames Valley & Wessex Neonatal Networks	Dr Katie Yallop

Confidential: For Review Only

Royal Lancaster Infirmary	Lancashire and South Cumbria Neonatal Network	Dr Joanne Fedee
Royal Oldham Hospital	Greater Manchester Neonatal Network	Dr Natasha Maddock
Royal Shrewsbury Hospital	Staffordshire, Shropshire & Black Country Newborn & Maternity Network	Dr Deshpande
Royal United Hospital	South West Region	Dr Stephen Jones
Russells Hall Hospital	Staffordshire, Shropshire & Black Country Newborn & Maternity Network	Dr Mahadevan
Salisbury District Hospital	Thames Valley & Wessex Neonatal Networks	Dr Nick Brown
Scunthorpe General Hospital	North Trent Neonatal Network	Dr Pauline Adiotomre
Southend Hospital	London - North East and North Middlesex Neonatal Network	Dr Arfa Khan
St Helier Hospital	London - South West Neonatal Network	Dr Salim Yasin
St Mary's Hospital, IOW	Thames Valley & Wessex Neonatal Networks	Dr Sian Butterworth
St Mary's Hospital, London	London - North West Neonatal Network	Dr Sunit Godambe
St Richard's Hospital	Thames Valley & Wessex Neonatal Networks	Dr Nick Brennan
Stepping Hill Hospital	Greater Manchester Neonatal Network	Dr Carrie Heal
Stoke Mandeville Hospital	Thames Valley & Wessex Neonatal Networks	Dr Sanjay Salgia
Tameside General Hospital	Greater Manchester Neonatal Network	Dr Jacqeline Birch
Taunton & Somerset Hospital	South West Region	Dr Chris Knight
Tunbridge Wells Hospital	South East Coast Neonatal ODN	Dr Hamudi Kisat
University Hospital Lewisham	London - South East Neonatal Network	Dr Jauro Kuna
University Hospital of North Durham	Northern Neonatal Network	Dr Mehdi Garbash
University Hospital of South Manchester	Greater Manchester Neonatal Network	Dr Gopi Vemuri
Victoria Hospital, Blackpool	Lancashire and South Cumbria Neonatal Network	Dr Chris Rawlingson
Warrington Hospital	Cheshire and Merseyside Neonatal Network	Dr Delyth Webb
Watford General Hospital	Beds-Herts Neonatal Network	Dr Sankara Narayanan
West Suffolk Hospital	Norfolk, Suffolk & Cambridgeshire Neonatal Network	Dr Ian Evans
Wexham Park Hospital	Thames Valley & Wessex Neonatal Networks	Dr Rekha Sanghavi
Whipps Cross University Hospital	London - North East and North Middlesex Neonatal Network	Dr Caroline Sullivan
Whiston Hospital	Cheshire and Merseyside Neonatal Network	Dr Rosaline Garr
Whittington Hospital	London - North Central Neonatal Network	Dr Wynne Leith
Worcestershire Royal Hospital	Midlands South West Newborn Network	Dr Andrew Gallagher
York District Hospital	Yorkshire Neonatal Network	Dr Guy Millman
Gloucestershire Royal Hospital	South West Region	Dr Simon Pirie
Arrowe Park Hospital	Cheshire and Merseyside Neonatal Network	Dr Anand Kamalanathan
Birmingham Heartlands Hospital	Midlands South West Newborn Network	Dr Phil Simmons
Birmingham Women's Hospital	Midlands South West Newborn Network	Dr Anju Singh
Bradford Royal Infirmary	Yorkshire Neonatal Network	Dr Sunita Seal
Chelsea & Westminster Hospital	London - North West Neonatal Network	Dr Mark Thomas
Derriford Hospital	South West Region	Dr Alex Allwood
Guy's & St Thomas' Hospital	London - South East Neonatal Network	Dr Timothy Watts
Homerton Hospital	London - North East and North Middlesex Neonatal Network	Dr Narendra Aladangady
hull royal infirmary	Yorkshire Neonatal Network	Dr Hassan Gaili
James Cook University Hospital	Northern Neonatal Network	Dr M Lal
King's College Hospital	London - South East Neonatal Network	Dr Ann Hickey
Lancashire Women & Newborn Centre	Lancashire and South Cumbria Neonatal Network	Dr Meera Lama
Leeds Neonatal Service	Yorkshire Neonatal Network	Dr Lawrence Miall
Leicester Neonatal Service	Midlands Central Neonatal Network	Dr Jonathan Cusack
Liverpool Women's Hospital	Cheshire and Merseyside Neonatal Network	Dr Bill Yoxall
Luton & Dunstable Hospital	Beds-Herts Neonatal Network	Dr Jennifer Birch
Medway Maritime Hospital	South East Coast Neonatal ODN	Dr Aung Soe
New Cross Hospital	Staffordshire, Shropshire & Black Country Newborn & Maternity Network	Dr Tilly Pillay
Norfolk & Norwich University Hospital	Norfolk, Suffolk & Cambridgeshire Neonatal Network	Dr Mark Dyke
Nottingham City Hospital	Trent Perinatal Network	Dr Steven Wardle
Nottingham University Hospital (QMC)	Trent Perinatal Network	Dr Steven Wardle

Confidential: For Review Only

Oxford University Hospitals, John Radcliffe Hospital	Thames Valley & Wessex Neonatal Networks	Dr Eleri Adams
Princess Anne Hospital	Thames Valley & Wessex Neonatal Networks	Dr Mike Hall
Queen Alexandra Hospital	Thames Valley & Wessex Neonatal Networks	Dr Charlotte Groves
Queen Charlotte's Hospital	London - North West Neonatal Network	Dr Sunit Godambe
Rosie Maternity Hospital, Addenbrookes	Norfolk, Suffolk & Cambridgeshire Neonatal Network	Dr Angela D'Amore
Royal Bolton Hospital	Greater Manchester Neonatal Network	Dr Paul Settle
Royal Preston Hospital	Lancashire and South Cumbria Neonatal Network	Dr Richa Gupta
Royal Stoke University Hospital	Staffordshire, Shropshire & Black Country Newborn & Maternity Network	Dr Alison Moore
Royal Sussex County Hospital	South East Coast Neonatal ODN	Dr P Amess
Royal Victoria Infirmary	Northern Neonatal Network	Dr Alan Fenton
NORTH BRISTOL NHS TRUST (SOUTHMEAD)	South West Region	Dr Paul Mannix
St George's Hospital	London - South West Neonatal Network	Dr Charlotte Huddy
St Mary's Hospital, Manchester	Greater Manchester Neonatal Network	Dr Ngozi Edi-Osagie
St Michael's Hospital	South West Region	Dr Pamela Cairns
St Peter's Hospital	South East Coast Neonatal ODN	Dr Peter Reynolds
Sunderland Royal Hospital	Northern Neonatal Network	Dr Majd Abu-Harb
The Jessop Wing, Sheffield	North Trent Neonatal Network	Dr Simon Clark
The Royal London Hospital - Constance Green	London - North East and North Middlesex Neonatal Network	Dr Vadivelam Murthy
University College Hospital	London - North Central Neonatal Network	Dr Giles Kendall
University Hospital Coventry	Midlands Central Neonatal Network	Dr Kate Blake
University Hospital of North Tees	Northern Neonatal Network	Dr Hari Kumar
William Harvey Hospital	South East Coast Neonatal ODN	Dr Vimal Vasu

Confidential: For Review Only

BMJ Paediatrics Open

Neonatal health care costs of very preterm babies in England: a retrospective analysis of a national birth cohort

Journal:	*BMJ Paediatrics Open*
Manuscript ID	bmjpo-2022-001818.R1
Article Type:	Original research
Date Submitted by the Author:	21-Mar-2023

Complete List of Authors:	Yang, Miaoqing; University of Oxford, Nuffield Department of Population Health<br>Campbell, Helen; University of Oxford, Nuffield Department of Population Health<br>Pillay, Thillagavathie; University Hospitals of Leicester NHS Trust, Neonatology; University of Wolverhampton Faculty of Science and Engineering, School of Medicine and Clinical Practice<br>Boyle, Elaine M.; University of Leicester, Health Sciences<br>Modi, Neena; Imperial College London, Neonatal Medicine<br>Rivero-Arias, Oliver; University of Oxford, Nuffield Department of Population Health

Keywords:	Health services research, Neonatology

SCHOLARONE™
Manuscripts

I, the Submitting Author has the right to grant and does grant on behalf of all authors of the Work (as defined in the below author licence), an exclusive licence and/or a non-exclusive licence for contributions from authors who are: i) UK Crown employees; ii) where BMJ has agreed a CC-BY licence shall apply, and/or iii) in accordance with the terms applicable for US Federal Government officers or employees acting as part of their official duties; on a worldwide, perpetual, irrevocable, royalty-free basis to BMJ Publishing Group Ltd ("BMJ") its licensees and where the relevant Journal is co-owned by BMJ to the co-owners of the Journal, to publish the Work in this journal and any other BMJ products and to exploit all rights, as set out in our licence.

The Submitting Author accepts and understands that any supply made under these terms is made by BMJ to the Submitting Author unless you are acting as an employee on behalf of your employer or a postgraduate student of an affiliated institution which is paying any applicable article publishing charge ("APC") for Open Access articles. Where the Submitting Author wishes to make the Work available on an Open Access basis (and intends to pay the relevant APC), the terms of reuse of such Open Access shall be governed by a Creative Commons licence – details of these licences and which Creative Commons licence will apply to this Work are set out in our licence referred to above.

Other than as permitted in any relevant BMJ Author's Self Archiving Policies, I confirm this Work has not been accepted for publication elsewhere, is not being considered for publication elsewhere and does not duplicate material already published. I confirm all authors consent to publication of this Work and authorise the granting of this licence.

Confidential: For Review Only

Neonatal health care costs of very preterm babies in England: a retrospective analysis of a national birth cohort

Miaoqing Yang[1,2], Helen Campbell[1], Thillagavathie Pillay[3], Elaine Boyle[4], Neena Modi[5], Oliver Rivero-Arias[1]

Running title: Neonatal costs of very preterm babies in England

[1] National Perinatal Epidemiology Unit (NPEU), Nuffield Department of Population Health, University of Oxford, Oxford, UK.
[2] Centre for Guidelines, National Institute for Health and Care Excellence, London, UK.
[3] Faculty of Science and Engineering, University of Wolverhampton, Wolverhampton, UK.
[4] Department of Population Health Sciences, University of Leicester, Leicester, UK.
[5] Section of Neonatal Medicine, School of Public Health, Chelsea and Westminster Hospital campus, Imperial College London, London, UK

Corresponding author
Associate Professor Oliver Rivero-Arias
National Perinatal Epidemiology Unit (NPEU)
Nuffield Department of Population Health
Old Road Campus
University of Oxford
OX3 7LF
Email: oliver.rivero@npeu.ox.ac.uk

Confidential: For Review Only

Abstract (244 words)

Objectives: Babies born between 27^{+0} and 31^{+6} weeks of gestation represent the largest group of very preterm babies requiring NHS care, however up-to-date cost figures for the UK are not currently available. This study estimates neonatal costs to hospital discharge for this group of very preterm babies in England.

Design: Retrospective analysis of resource use data recorded within the National Neonatal Research Database (NNRD).

Setting: Neonatal units in England.

Patients: Babies born between 27^{+0} and 31^{+6} weeks of gestation in England and discharged from a neonatal unit between 2014 and 2018.

Main outcome measures: Days receiving different levels of neonatal care were costed, along with other specialised clinical activities. Mean resource use and costs per baby are presented by gestational age at birth, along with total costs for the cohort.

Results: Based upon data for 28,154 very preterm babies, the annual total costs of neonatal care were estimated to be £262 million, with 95% of costs attributable to routine daily care provided by units. The mean (SD) cost per baby of daily care varied by gestational age at birth; £75,594 (£34,874) at 27 weeks as compared with £27,401 (£14,947) at 31 weeks.

Conclusions: Neonatal healthcare costs for very preterm babies vary substantially by gestational age at birth. The findings presented here are a useful resource to stakeholders including NHS managers, clinicians, researchers, and policy makers.

Confidential: For Review Only

What is already known on this topic

Existing cost estimates for very preterm care in England are now over a decade old and may not reflect modern care practices.

What this study adds

This study has generated current cost estimates at the level of the individual baby and for the cohort as a whole in England and confirmed the previously reported inverse relationship between healthcare costs and gestational age at birth.

How this study might affect research, practice or policy

The outputs from this work provide a valuable resource for research assessing the economic implications of interventions to prevent preterm birth, the provision of care for preterm babies, as well as helping inform NHS resource allocation decisions.

Confidential: For Review Only

Introduction

Babies born between 27^{+0} and 31^{+6} weeks of gestation (hereafter called 'born at 27–31 weeks') represent the largest group of very preterm babies requiring NHS care.[1] These babies also account for about 12% of all viable preterm babies born in England and usually require admission to a neonatal unit.[1]

Previous work in the UK and elsewhere has attempted to estimate the healthcare and societal cost of preterm birth.[2-6] However, there is marked variability in reported cost estimates, due to differences in study perspectives, included babies, data sources and methods used to assign costs. A number of studies have reported cost estimates associated with the initial period of hospitalisation for babies born at 27-31 weeks.[2 3 6] Whilst these studies have shown the costs of neonatal care for very preterm babies to be inversely related to gestational age at birth, estimates for the UK are now over a decade old and there is a need for new analyses. As part of the OPTI-PREM suite of studies aimed at optimising neonatal service provision for very preterm babies in England, we conducted a retrospective cohort study to describe the levels of neonatal care, key specialist procedures, and healthcare costs attributable to the management of these babies.[1] The analysis makes use of healthcare resource use data routinely collated within the National Neonatal Research Database (NNRD) on babies admitted to all neonatal units in England.

Methods

Study design and population

For this retrospective analysis, we included all admissions to neonatal units in England for babies born at 27-31 weeks and discharged or died between January 2014 and December 2018. Admissions were identifiable through the NNRD, which was created in 2007 to support activities including audit, evaluations, and clinical, health services and policy research, and is maintained at the Neonatal Data Analysis Unit (NDAU) at Imperial College London [https://www.imperial.ac.uk/neonatal-data-analysis-unit/]. All NHS neonatal units in England, Scotland, Wales, and the Isle of Man use their Electronic Patient Record (EPR) supplier systems to routinely submit detailed information to the NNRD on the

Confidential: For Review Only

clinical care they provide to babies. Submitted data are quality-assured and curated to a research standard. Data comprise demographics, diagnoses, health outcomes, and daily interventions (including the level of care, namely intensive, high dependency, special, or normal, provided each day) and treatments administered during the inpatient episode.

Data were extracted from the NNRD on 5th October 2020. Daily intervention data and treatment episode data were received in two separate datasets and were linked by means of participant identification number. Data were imported into Stata software and manipulated such that for each baby, a daily intervention record (which included the level of care provided) was available for each day of the admission episode. Babies missing daily intervention records or for whom daily records were available but level of care data had not been recorded, were excluded from the primary analysis.

Resource use and costs

The cost analysis was conducted from the perspective of the National Health Service (NHS) in England. To calculate the costs associated with the routine daily neonatal care received by each baby, we multiplied the number of days spent receiving each level of care, by level-specific national average bed day costs sourced from the 2018/19 National Schedule of NHS Costs.[7] Such costs are estimated by assigning each level of care a healthcare resource group (HRG) code. HRGs are groupings of clinically meaningful activities made primarily on the basis of diagnosis and procedure codes, and within the NHS, are the 'units' of health care for which providers receive payment. Costs assigned to each HRG are based upon nationallyestimated tariffs developed to adequately cover the cost of providing high quality and cost-effective care. . For this analysis the neonatal bed day costs used were: intensive care (HRG XA01Z), high dependency care (HRG XA02Z), special care without carer resident alongside baby (HRG XA03Z), special care with carer resident alongside baby (HRG XA04Z), and normal care (HRG XA05Z). Supplementary Table A1 shows these unit costs. Detailed information on the items included within each daily level of care cost are available from NHS England.[8]

Not all major clinical activities are included in these neonatal critical care HRGs. After in-depth discussions with clinical experts, we identified five high cost non-routine procedures captured within the NNRD, that were considered to be important in this particular population. These were nitric oxide,

Confidential: For Review Only

surfactant replacement, total parental nutrition (TPN) (over 14 days of use), palivizumab use and surgical care. Types of neonatal surgery were identified from their corresponding OPCS and ICD-10 codes for costing purposes, and costs for other activities were obtained from various sources as detailed in supplementary Table A1. NNRD data for these five non-routine procedures were assumed to be complete, with no entry in the corresponding dataset fields taken as an indication that the procedure did not take place.

Statistical analysis

When describing neonatal and maternal characteristics, counts and proportions and means and standard deviations (SD) were used for categorical and continuous variables respectively. Analyses of resource use data and costs were performed by gestational age at birth. Means (SD) were used to summarise days provided at each level of care with associated 95% confidence interval following a Poisson distribution. Counts were made of the numbers of babies for whom non-routine procedures were recorded, along with the number of cases (for example surgeries) or days (for example for nitric oxide) for which such treatment was given. [trend] Total costs were reported for the cohort as a whole and again by gestational age at birth. A secondary analysis using multiple imputation of the level of care costs was carried out to understand the impact of the missing information on level of care in the overall cost results (see supplementary Multiple Imputation Methods). Analyses were conducted using Stata MP.[9]

Patient and Public Involvement

An OPTI-PREM parent panel of mothers and fathers of babies born at 27-31 weeks in England was established with support from the national charity BLISS. The parent panel engaged in the study design and review of the funding protocol, development of parent information leaflets and neonatal unit posters. They attended team and study steering committee meetings, provided input at national stakeholder discussions, and contributed with the interpretation of final results, and our dissemination strategy.

Confidential: For Review Only

Results

Study population

Within the NNRD data extraction were 29,842 infants, with a total of 1,512,446 daily records and 46,746 episode records. Following the removal of infants with missing daily record information (n=1,292) and those with missing data on the level of daily care provided (n=377), a total of 28,173 babies (94% of the starting cohort of 29,842) remained and were included in the cost analysis (full details of the record matching process and data cleaning can be found in supplementary Figure A1). Table 1 summarises the characteristics of these babies and their mothers. A comparison between babies included in the analysis and those excluded due to missing data (n=1,669) was performed and showed the latter were more likely to have been born at earlier gestations (for example 17% versus 12% were born at 27 weeks; see supplementary Table A2). Despite this, birth statistics for the study cohort were still comparable to those of all very preterm babies born in England between 2016 to 2018 (data compiled by the Office for National Statistics; see supplementary Table A3).

Resource use

Table 2 shows the mean (SD) per baby duration spent receiving each level of daily care according to gestational age at birth. Data show a consistent inverse relationship between gestational age at birth and the intensity of daily care provided. Figure 1 plots the mean durations along with mean overall length of stay on the neonatal unit and illustrates the longer durations of higher intensity care (and indeed overall neonatal care) provided to babies born at earlier gestations. For example, babies born at 27 weeks spent on average, 18.1 days (SD=15.7 days) receiving intensive care and 26.8 days (SD=22 days) receiving high-dependency care, while babies born at 31 weeks received an average of 3.33 days (SD=6.7 days) of intensive care and 5.1 days (SD=7.5 days) of high-dependency care. Also of note is that across all gestational age at birth groups, days spent receiving special care without a carer present accounted for the largest proportion of days spent on the neonatal unit.

Supplementary Table A4 presents the number of babies who underwent hospital transfers and received key non-routine procedures, again by gestational age at birth. Greater resource utilisation amongst infants born at earlier gestations can be seen.

Confidential: For Review Only

Costs

The mean (SD) cost per baby for the various levels of care and non-routine procedures are shown by gestational age at birth in Table 3 using a complete case analysis. As expected, and given the observations for resource use, costs can be seen to increase as gestational age at birth decreases. Mean (SD) routine daily care costs for a baby born at 27 weeks, for example, were, at £75,594 (£34,874), 2.8 times greater than costs for a baby born at 31 weeks (£27,401 (£14,974)). The final column in Table 3 shows that for the 2014-2018 cohort as a whole, just over 50% of total costs were attributable to the provision of intensive and high dependency care, with a further 42% coming from the provision of special care (without a carer). Further exploration of the composition of total costs in Figure 2 reveals variation by gestational age at birth. Almost 70% of total costs for babies born at 27 weeks were associated with the use of intensive- and high-dependency care, while the proportion decreased to only 36% for babies born at 31 weeks. The usage, and thus cost of special care (without a carer present) increases with increasing gestational age at birth. The results of the multiple imputation analysis for the level of care costs (supplementary Table A5) were similar to the complete case analysis. Across all gestational at birth groups, non-routine procedures accounted for only a very small proportion of total costs.

The overall total cost estimated for the complete case cohort (n = 28,154) over the five years from 2014 to 2018 (£1.3billion in Table 3) suggests the annual costs of neonatal care for babies born between 27 and 31 gestational weeks and admitted to a neonatal unit in England to be around £262 million, with the provision of routine daily care on these units accounting for 96% (£252 million) of overall costs. Using the overall sample of 29,842 infants would increase the total cohort costs and the estimates of annual total costs shown in Table 3 from £1,309,192,377 to £1,393,642,826 and the annual total cost from £262 million to £279 million.

Confidential: For Review Only

Discussion

Main findings

Analyses at the level of the individual baby revealed the main cost drivers to be the daily neonatal care provided to support babies. Analyses by gestational age at birth provided further evidence of the previously reported inverse relationship between resource use and healthcare costs and the degree of prematurity, with care for a baby born at 27 weeks estimated to cost almost three times more than for a baby born at 31 weeks.[5] Our work also provides valuable information on the contribution of different resource components to overall costs and illustrates how the mix of intensive of care required by these babies varies with gestational age at birth.

A small number of studies have previously estimated neonatal care costs for preterm babies, one of the most recent being by Rios et al. in Canada[6] which also used individual patient level resource use data from a neonatal database (the Canadian Neonatal Network Database) and included around 8,000 babies born between 27 and 31 weeks gestation. A comparison between the costs estimated for the babies in the Rios et al. study and the costs presented here (currency conversion from Can $ to UK £ made using Purchasing Power Parities) revealed the Canadian cost estimates to be consistently lower across all gestational age groups.[10] The most obvious explanation for these differences lies with the scope of the neonatal databases used by each study and the resulting implications for duration of neonatal unit stay. In general Rios et al. reported consistently lower mean lengths of stay for all gestional ages compared to our estimates due to their focus on intensive care units and the exclusion of the costs of care in lower dependency neonatal units. Thus the Rios et al. estimates do not reflect the true costs of healthcare provision for this group of babies.

In 2009, Mangham and colleagues used a decision analytic model to estimate neonatal costs for a hypothetical cohort of preterm babies born in England and Wales.[2] Model parameters were sourced from various cohort studies and the mean cost for a very pre-term baby (<33 weeks) was estimated to be £57,726 (95% CI: 28,779 to 94,868) (2006 GBP). With that analysis using a different methodology, reporting results for a wider gestational age range, and now being well over a decade old, comparisons with the estimates reported here are challenging. Whilst a number of more recent UK studies have been published on the costs of pre-term birth, these works have focussed specifically on births at other

Confidential: For Review Only

gestations (i.e. moderate and late preterm births or extremely preterm births).[2 4 11 12] One reassuring finding however is that the costs of neonatal care for very preterm births presented here fall between the costs reported for moderate / late preterm infants and for extremely preterm infants in the UK.

Strengths and limitations

This study makes a number of contributions to the published literature. Firstly, existing UK cost estimates for neonatal care following very preterm birth are over a decade old and so are unlikely to reflect current standards of practice.[2] This study provides up-to-date figures based on a large cohort of more than 28,000 babies who were discharged from or died within neonatal units across England between 2014 and 2018. Secondly, previous UK cost studies in the area have mainly relied on data synthesised from secondary sources to estimate costs. In utilising the NNRD, this study has been able to employ detailed and quality assured individual participant data derived from electronic patient records (EPR). Furthermore, the dataset offered a unique opportunity to capture near population-wide data (and thus costs) on day-to-day care provided across all neonatal units without imposing any additional burden on study participants. Thirdly, the richness of the dataset permitted us not only to cost routine daily care provided at differing levels of intensity, but also to consider the cost implications of a number of major non-routine procedures, which are not captured within the HRG codes for neonatal critical care. Finally, by generating up to date estimates of the mean resource use and costs of neonatal care for very preterm babies in England, this study provides valuable data of interest to a range of stakeholders, including NHS managers, clinicians providing care, researchers assessing the economic implications of therapies and interventions to prevent and treat preterm birth, and decision makers charged with implementing new policies and allocating resources.

A number of limitations must also be acknowledged. This study considered only healthcare costs associated with the initial period of hospitalisation, though the economic consequences of preterm birth extend over prolonged periods of time, in some cases over the lifetime of the individual.[2 12 13] Preterm birth can lead to additional healthcare as well as social care needs and special educational needs throughout childhood.[14 15] Further research is needed to adequately capture these wider costs accurately, including the economic costs to families while their preterm baby was hospitalised, and then throughout their lives. A further limitation is the exclusion from the analysis of babies with missing data

Confidential: For Review Only

on daily care provision, who were shown to have been born at earlier gestations than babies with complete data (supplementary Table A2). Whilst these babies accounted for only 6% of the initial NNRD cohort and data showed no differences in gestational age at birth between the babies with complete data and all very preterm births registered in England (supplementary Table A4), we observed small significant differences in baseline characteristics between babies with missing and complete data. We conducted a multiple imputation approach to understand the implications of these differences and our results suggested limited impact of the missing data in the overall cost results.

Conclusion

This study has generated up-to-date estimates of the costs of providing neonatal care to very preterm babies in England. Resource use and costs vary by gestational age at birth. The outputs from this work can be used to inform clinical and budgetary service planning and ensure the efficient allocation of health care resources.

Confidential: For Review Only

References

1. Pillay T, Modi N, Rivero-Arias O, et al. Optimising neonatal service provision for preterm babies born between 27 and 31 weeks gestation in England (OPTI-PREM), using national data, qualitative research and economic analysis: a study protocol. *BMJ Open* 2019;9(8):e029421. doi: 10.1136/bmjopen-2019-029421 [published Online First: 20190822]
2. Mangham LJ, Petrou S, Doyle LW, et al. The cost of preterm birth throughout childhood in England and Wales. *Pediatrics* 2009;123(2):e312-27. doi: 10.1542/peds.2008-1827
3. Korvenranta E, Linna M, Rautava L, et al. Hospital costs and quality of life during 4 years after very preterm birth. *Arch Pediatr Adolesc Med* 2010;164(7):657-63. doi: 10.1001/archpediatrics.2010.99
4. Khan KA, Petrou S, Dritsaki M, et al. Economic costs associated with moderate and late preterm birth: a prospective population-based study. *BJOG* 2015;122(11):1495-505. doi: 10.1111/1471-0528.13515 [published Online First: 20150722]
5. Petrou S, Yiu HH, Kwon J. Economic consequences of preterm birth: a systematic review of the recent literature (2009-2017). *Arch Dis Child* 2019;104(5):456-65. doi: 10.1136/archdischild-2018-315778 [published Online First: 20181109]
6. Rios JD, Shah PS, Beltempo M, et al. Costs of Neonatal Intensive Care for Canadian Infants with Preterm Birth. *J Pediatr* 2021;229:161-67 e12. doi: 10.1016/j.jpeds.2020.09.045 [published Online First: 20200923]
7. NHS England. National Cost Collection Data Publication. National Schedule of NHS Costs 2018/2019 2019 [Available from: https://www.england.nhs.uk/publication/2018-19-national-cost-collection-data-publication/ accessed 12th November 2022.
8. NHS England. Clinicians quick reference guide – Neonatal Critical Care HRGs 2016 & NCCMDS Codes: National Casemix Office; 2016 [Available from: https://www.networks.nhs.uk/nhs-networks/staffordshire-shropshire-and-black-country-newborn/documents/nhs-england-quick-reference-neonatal-critical-care-hrgs-2016-nccmds-codes accessed 2nd March 2023.
9. Stata Statistical Software Release 15 [program]. College Station, TX: StataCorp LP, 2015.
10. Eurostat-OECD. Methodological Manual on Purchasing Power Parities. Luxembourg: Publications Office of the European Union: OECD 2012.
11. Hinchliffe SR, Seaton SE, Lambert PC, et al. Modelling time to death or discharge in neonatal care: an application of competing risks. *Paediatr Perinat Epidemiol* 2013;27(4):426-33. doi: 10.1111/ppe.12053 [published Online First: 20130415]
12. Kim SW, Andronis L, Seppanen AV, et al. Economic costs at age five associated with very preterm birth: multinational European cohort study. *Pediatr Res* 2022;92(3):700-11. doi: 10.1038/s41390-021-01769-z [published Online First: 20211112]
13. Ni Y, Mendonca M, Baumann N, et al. Social Functioning in Adults Born Very Preterm: Individual Participant Meta-analysis. *Pediatrics* 2021;148(5) doi: 10.1542/peds.2021-051986 [published Online First: 20211026]
14. Alterman N, Johnson S, Carson C, et al. Gestational age at birth and child special educational needs: a UK representative birth cohort study. *Arch Dis Child* 2021;106(9):842-48. doi: 10.1136/archdischild-2020-320213 [published Online First: 20210122]
15. Alterman N, Johnson S, Carson C, et al. Gestational age at birth and academic attainment in primary and secondary school in England: Evidence from a national cohort study. *PloS one* 2022;17(8):e0271952. doi: 10.1371/journal.pone.0271952 [published Online First: 20220817]

For peer review only

Data availability statement

The National Neonatal Research Database can be accessed by making a request to the Neonatal Data Analysis Unit at Imperial College London through the Health Data Research UK Gateway (https://web.www.healthdatagateway.org/search?search=NNRD&datasetSort=latest&tab=Datasets).

Ethics statements

Patient consent for publication

Not required.

Ethics approval

Research ethics approval for the OPTI-PREM programme of work was obtained through the national Integrated Research Application System (IRAS, reference number 212 304 and research ethics committee reference number 17/NE/0800; North East – Tyne and Wear South).

Funding

This work is supported by the National Institute for Health Research, Health Services and Delivery Research Stream, project number 15/70/104 CRN accrual was approved by the NIHR for the period (1 August 2017 to 31 August 2018).

Competing interests

Authors declare no conflict of interests.

Contributors

ORA developed the study concept and aims, and developed the study proposal with MY, TP, EB, and NM. MY and ORA conducted the statistical analyses. MY, HC and ORA wrote the first draft of the manuscript. All other authors reviewed the draft and provided critical input. All authors approve the final version.

Acknowledgments

Confidential: For Review Only

To parents, patients and families participating in the OPTI-PREM study, and to the OPTI-PREM Parent Panel, for its support. The OPTI-PREM study is grateful to its NIHR Project Steering Committee: Andrew Ewer (chair), Stavros Petrou, Gillian Santorelli, Josie Anderson, David Loughton, Lisa Stanton, Karen Luyt, and to the National Neonatal Collaborative. We are indebted to all members of the UK Neonatal Collaborative that participated and made this study possible (see supplementary file for list of members).

The OPTI-PREM Study team: Elaine M Boyle, Neena Modi, Oliver Rivero-Arias, Bradley Manktelow, Sarah E Seaton, Natalie Armstrong, Miaoqing Yang, Abdul Qader T Ismail, Vasiliki Bountziouka, Caroline S Cupit, Alexis Paton, Victor L Banda, Elizabeth S Draper, Kelvin Dawson and Thillagavathie Pillay (Chief Investigator).

Confidential: For Review Only

Table 1: Baby and maternal characteristics of study cohort - data are frequencies (percentages) unless otherwise stated

	Cohort (n = 28,173)
	**n (%)**
Baby characteristics	
**Gestational age at birth**	
27 weeks	3,296 (11.7%)
28 weeks	4,370 (15.5%)
29 weeks	5,036 (17.9%)
30 weeks	6,625 (23.5%)
31 weeks	8,827 (31.3%)
Missing	*19 (0.1%)*
**Gender of baby**	
Male	15,363 (54.5%)
Female	12,755 (45.3%)
Missing	*55 (0.2%)*
**Number of fetuses**	
Singleton birth	20,555 (73.0%)
Multiple birth	7,598 (27.0%)
Missing	*20 (0.1%)*
**Birthweight (g) – mean (SD)**	1330.2 (332.2)
Missing	*82 (0.3%)*
**Apgar score at 5min – mean (SD)**	8.1 (1.8)
Missing	*2,877 (10.2%)*
**Died in neonatal care**	985 (3.5%)
Missing	*0 (0.0%)*
Maternal characteristics	
**Age (years) – mean (SD)**	30.7 (6.3)
Missing	*279 (1.0%)*
**Ethnicity**	
White	17,647 (62.6%)
Black	2,011 (7.1%)
Asian	3,144 (11.2%)
Mixed	433 (1.5%)
Other	483 (1.7%)
Missing	*4,455 (15.8%)*
**Diabetes**	2,378 (8.4%)
Missing	*10,736 (38.1%)*
**Hypertension**	3,506 (12.4%)
Missing	*10,460 (37.1%)*
**Infection**	3,029 (10.6%)
Missing	*10,595 (37.6%)*
**Mode of delivery**	
Vaginal-spontaneous	8,200 (29.1%)
Vaginal-instrumental	765 (2.7%)
Caesarean section	17,691 (62.3%)
Missing	*1,517 (5.4%)*

Confidential: For Review Only

**Quintiles of IMD**	
1st Q, least deprived	3,355 (11.9%)
2nd Q	3,761 (13.4%)
3rd Q	4,516 (16.0%)
4th Q	5,930 (21.1%)
5th Q, most deprived	8,057 (28.6%)
Missing	*2,554 (9.1%)*

SD: standard deviation; IMD: Index of Multiple Deprivation; Q: quintile

Confidential: For Review Only

Table 2: Summary of type and duration of daily care received by very preterm babies during neonatal unit admissions in England for the period 2014-2018

	Level of care provided (HRG Code)				
**Gestational age at birth**	**Intensive care (XA01Z)**	**High-dependency care (XA02Z)**	**Special care without carer (XA03Z)**	**Special care with carer (XA04Z)**	**Normal care (XA05Z)**
**27 weeks gestation (n=3,296)**					
Number of days of care	59,494	88,212	100,770	5,113	0
Number of babies receiving care	3,275	3,065	3,021	1,681	0
Mean (SD) duration of care (days)*	18.1 (15.7)	26.8 (22.0)	30.5 (18.0)	1.5 (2.5)	0.0 (0.0)
95% CI	17.9 to 18.2	26.6 to 27.0	30.4 to 30.7	1.5 to 1.6	0.0 to 0.0
Median (range) duration of care (days)*	14.0 (0.0 to 174.0)	23.0 (0.0 to 243.0)	30.5 (0.0 to 161.0)	1.0 (0.0 to 31.0)	0.0 (0.0 to 0.0)
**28 weeks gestation (n=4,370)**					
Number of days of care	59,440	83,399	141,630	7,345	4
Number of babies receiving care	4,298	4,050	4,097	2,268	1
Mean (SD) duration of care (days)*	13.6 (13.4)	19.1 (19.5)	32.4 (16.5)	1.7 (3.0)	0.0 (0.1)
95% CI	13.5 to 13.7	18.9 to 19.2	32.2 to 32.6	1.6 to 1.7	0.0 to 0.0
Median (range) duration of care (days)*	11.0 (0.0 to 246.0)	14.0 (0.0 to 203.0)	33.0 (0.0 to 127.0)	1.0 (0.0 to 44.0)	0.0 (0.0 to 4.0)
**29 weeks gestation (n=5,036)**					
Number of days of care	47,728	57,613	165,090	8,449	0
Number of babies receiving care	4,774	4,631	4,868	2,671	0
Mean (SD) duration of care (days)*	9.5 (10.2)	11.4 (14.1)	32.8 (13.8)	1.7 (3.0)	0.0 (0.0)
95% CI	9.4 to 9.6	11.4 to 11.5	32.6 to 32.9	1.6 to 1.7	0.0 to 0.0
Median (range) duration of care (days)*	8.0 (0.0 to 258.0)	7.0 (0.0 to 196.0)	33.0 (0.0 to 119.0)	1.0 (0.0 to 45.0)	0.0 (0.0 to 0.0)
**30 weeks gestation (n=6,625)**					
Number of days of care	36,599	51,479	199,283	11,428	3
Number of babies receiving care	5,196	5,724	6,471	3,582	1
Mean (SD) duration of care (days)*	5.5 (7.1)	7.8 (11.1)	30.1 (12.1)	1.7 (2.9)	0.0 (0.0)
95% CI	5.5 to 5.6	7.7 to 7.8	29.9 to 30.2	1.7 to 1.8	0.0 to 0.0
Median (range) duration of care (days)*	4.0 (0.0 to 133.0)	5.0 (0.0 to 213.0)	30.0 (0.0 to 115.0)	1.0 (0.0 to 37.0)	0.0 (0.0 to 3.0)
**31 weeks gestation (n=8,827)**					
Number of days of care	29,094	44,547	219,355	14,948	9
Number of babies receiving care	5,124	7,055	8,672	4,718	2
Mean (SD) duration of care (days)*	3.3 (6.7)	5.1 (7.5)	24.9 (10.5)	1.7 (2.8)	0.0 (0.1)
95% CI	3.3 to 3.3	5.0 to 5.1	24.7 to 25.0	1.7 to 1.7	0.0 to 0.0
Median (range) duration of care (days)*	1.0 (0.0 to 201.0)	3.0 (0.0 to 154.0)	24.0 (0.0 to 240.0)	1.0 (0.0 to 36.0)	0.0 (0.0 to 7.0)

SD: standard deviation, CI: parametric confidence interval using Poisson distribution, *Estimated across all babies within a gestational age group

Confidential: For Review Only

Table 3: Mean (SD) per baby and total neonatal care costs (2018/19 UK £) for very preterm babies in England over the period 2014-2018 using complete case analysis (n = 28,154)*

	27 weeks gestation (n = 3,296)	28 weeks gestation (n = 4,370)	29 weeks gestation (n = 5,036)	30 weeks gestation (n = 6,625)	31 weeks gestation (n = 8,827)	Total neonatal care costs (percentage) for 2014-2018
**Resource use item**	**Mean (SD) cost per baby**	**Mean (SD) cost per baby**	**Mean (SD) cost per baby**	**Mean (SD) cost per baby**	**Mean (SD) cost per baby**	
**Level of daily care**						
Intensive care	£27,690 (£24,039)	£20,865 (£20,473)	£14,548 (£15,603)	£8,482 (£10,887)	£5,062 (£10,235)	£356,747,496 (27.3%)
High-dependency care	£26,956 (£22,157)	£19,221 (£19,584)	£11,523 (£14,203)	£7,828 (£11,186)	£5,083 (£7,567)	£327,832,878 (25.0%)
Special care without carer	£20,187 (£11,880)	£21,407 (£10,921)	£21,659 (£9,110)	£19,875 (£8,019)	£16,423 (£6,939)	£546,285,433 (41.7%)
Special care with carer	£761 (£1,235)	£826 (£1,454)	£824 (£1,453)	£847 (£1,444)	£831 (£1,378)	£23,230,160 (1.8%)
Normal care	£0 (£0)	£0.47 (£31)	£0 (£0)	£0.23 (£19)	£0.52 (£40)	£8,224 (0.0%)
**Total level of care costs**	**£75,594 (£34,874)**	**£62,319 (£30,841)**	**£48,554 (£23,426)**	**£37,033 (£18,276)**	**£27,401 (£14,947)**	**£1,254,104,191 (95.8%)**
**Hospital transfers**	**£1,120 (£1,435)**	**£886 (£1,277)**	**£712 (£1,148)**	**£566 (£997)**	**£423 (£860)**	**£18,660,165 (1.4%)**
**Non-routine procedures**						
Nitric oxide	£105 (£557)	£81 (£436)	£49 (£354)	£36 (£312)	£19 (£148)	£1,356,929 (0.1%)
Surfactant replacement	£1,006 (£2,052)	£933 (£1,980)	£784 (£1,970)	£497 (£995)	£351 (£1,340)	£17,742,128 (1.4%)
TPN >14 days use	£930 (£2,203)	£609 (£1,838)	£316 (£1,398)	£147 (£883)	£71 (£550)	£8,919,898 (0.7%)
Palivizumab	£172 (£1,188)	£152 (£1,518)	£74 (£1,007)	£40 (£716)	£16 (£467)	£2,022,625 (0.2%)
ROP surgery	£61 (£398)	£21 (£223)	£12 (£165)	£8.9 (£138)	£2.9 (£71)	£439,674 (0.03%)
Neonatal surgery	£456 (£2,053)	£358 (£1,844)	£206 (£1,325)	£135 (£996)	£107 (£934)	£5,946,767 (0.5%)
**Total non-routine procedures costs**	**£2,730 (£4,594)**	**£2,153 (£4,228)**	**£1,442 (£3,333)**	**£864 (£2,143)**	**£567 (£1,955)**	**£36,428,021 (2.78%)**
Total neonatal care costs for 2014-2018						***£1,309,192,377***
Annual total neonatal costs						***£261,838,475***

SD: standard deviation, NHS: National Health Service, TPN: total parenteral nutrition, ROP: retinopathy of prematurity
* 19 babies had missing gestational age information

Confidential: For Review Only

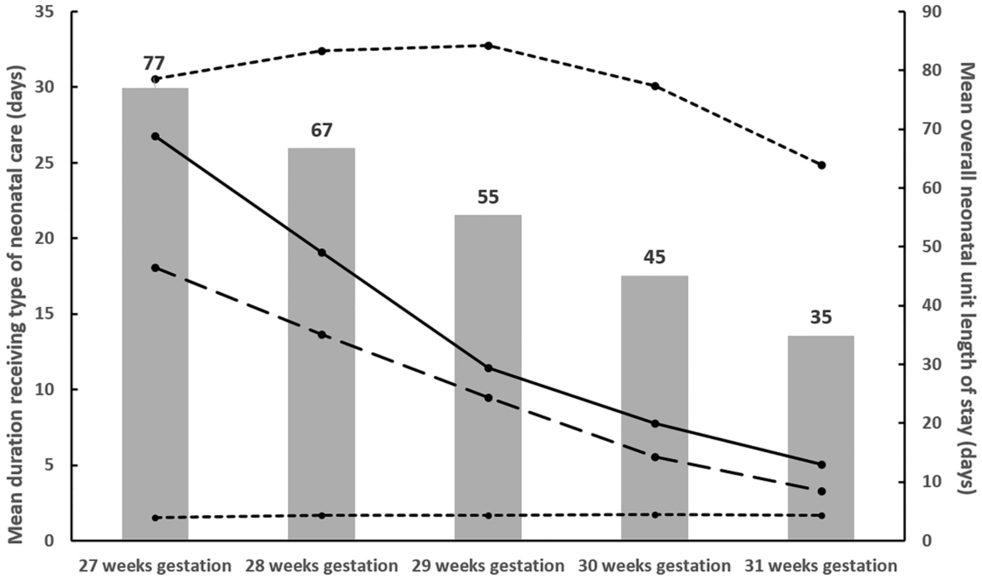

Figure 1 - Mean durations of different levels of daily care provided and overall length of stay per baby (in days) during neonatal unit admissions in England for the period 2014-2018. Data shown by gestational age at birth

463x275mm (300 x 300 DPI)

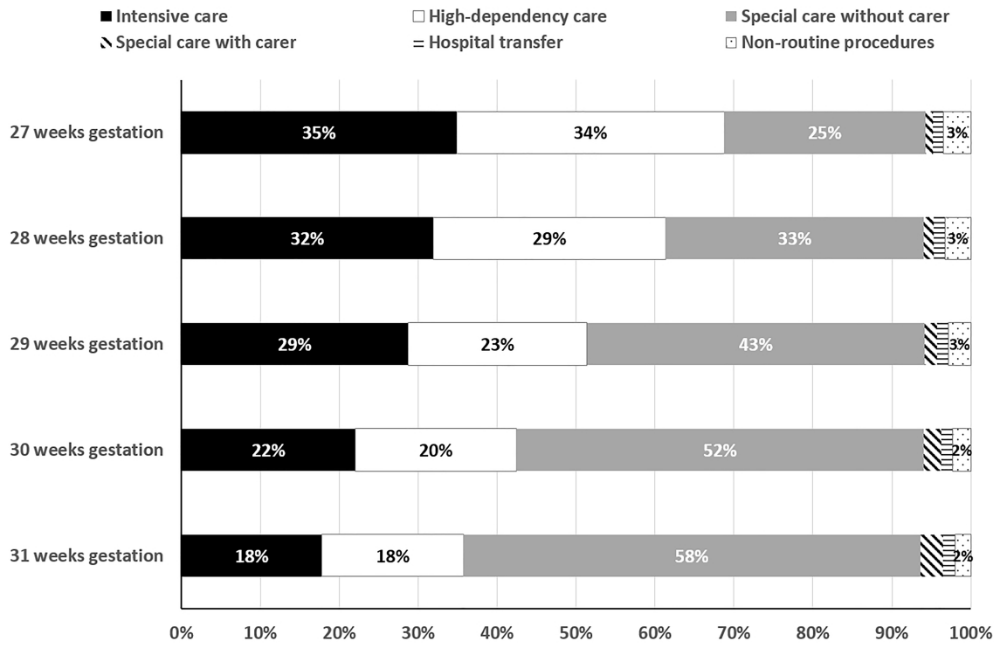

Figure 2 - Proportion of neonatal care cost attributable to different cost categories, by gestational age at birth

420x277mm (300 x 300 DPI)

Supplementary file

Multiple Imputation Methods

An imputation model was constructed that included complete data on baseline baby and maternal characteristics, and the individual level of care costs (intensive care, high-dependency care, special care without carer, special care with carer and normal care. Baseline characteristics included gestational age at birth, gender, number of fetuses, birthweight, neonatal death, maternal age and mode of delivery. All baseline covariates were subject to a small number of missing data that was imputed using conditional mean imputation prior to inclusion in the imputation model. A predictive mean matching estimation using chained equations with 50 imputations was implemented [1]. Mean estimates and estimates of standard errors were combined between imputed datasets using Rubin's rule [2] without any adjustment. Combined mean cost estimates and adjusted standard errors (SE) by gestational week across imputed datasets are shown to report the results of the multiple imputation analysis.

Confidential: For Review Only

Figure A1: Flow of NNRD data available to estimate neonatal care costs for the very preterm babies discharged from neonatal units in England between 2014-2018.

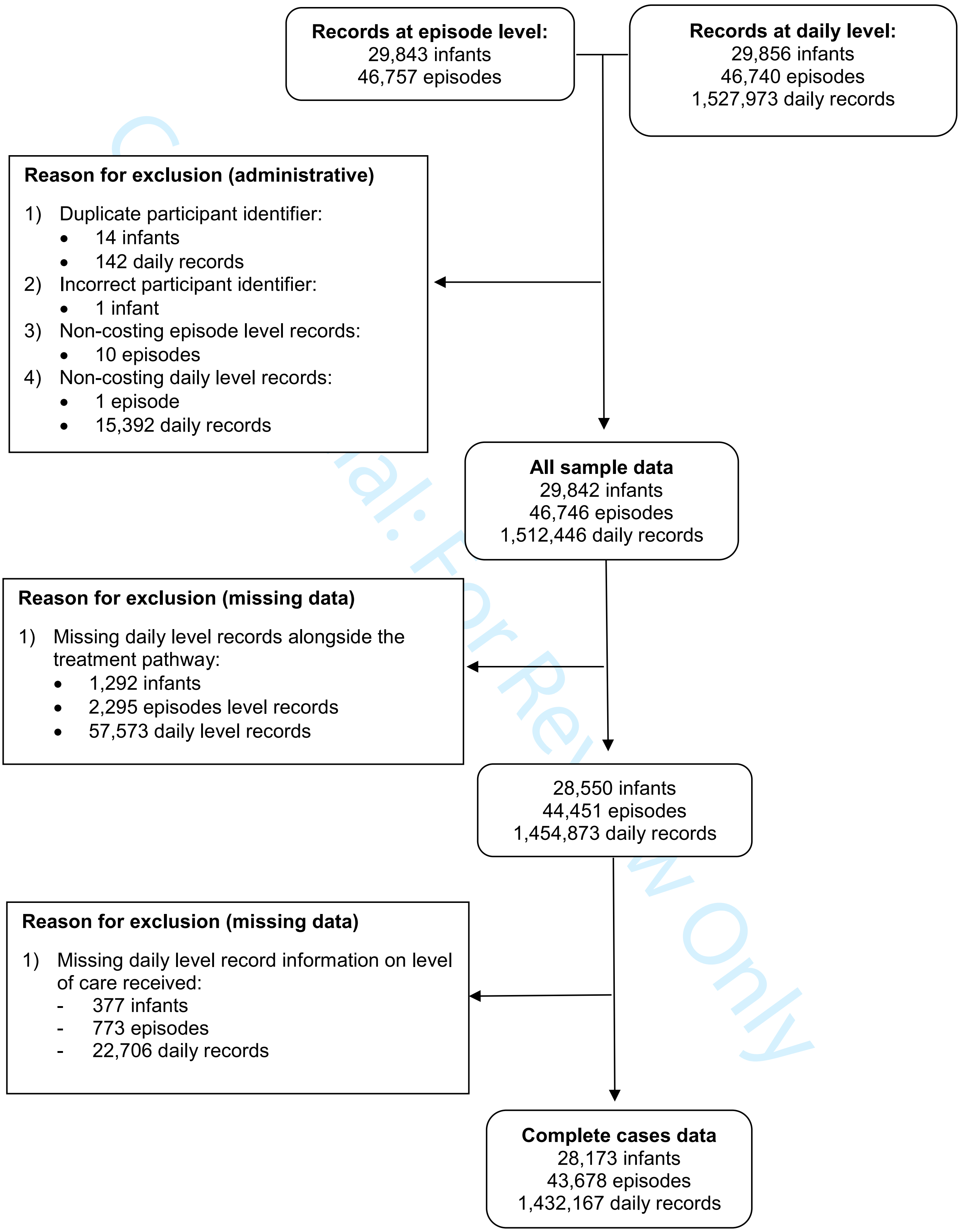

Confidential: For Review Only

Table A1: Unit costs (expressed in 2018/19 UK pound sterling) used within the cost analysis

Resource Use Item	Unit cost 2018/19 UK £	Source
Neonatal unit inpatient bed days		
- intensive care level	£1,531	National Cost Collection Data Publication. National Schedule of NHS Costs 2018/19. HRG code XA01Z [3]
- high dependency care level	£1,007	National Cost Collection Data Publication. National Schedule of NHS Costs 2018/19. HRG code XA02Z [3]
- special care level, (carer not resident alongside baby)	£661	National Cost Collection Data Publication. National Schedule of NHS Costs 2018/19. HRG code XA03Z [3]
- special care level, (carer resident at cot-side and caring for baby)	£493	National Cost Collection Data Publication. National Schedule of NHS Costs 2018/19. HRG code XA04Z [3]
- normal care level	£514	National Cost Collection Data Publication. National Schedule of NHS Costs 2018/19. HRG code XA05Z [3]
Neonatal Critical Care, Transportation	£1,257	National Cost Collection Data Publication. National Schedule of NHS Costs 2018/19. HRG code XA06Z [3]
Inhaled nitric oxide (iNO) (per day)	£267	European iNO Registry for Liverpool Women's NHS Foundation Trust
*Surfactant replacement – poractant alfa (per day, by birth weight)**		
- Birth weight ≤0.6kg (1 bottle 1.5ml)	£282	British National Formulary online: NHS indicative price [4]
- 0.7kg≤ Birth weight ≤1.2kg (1 bottle 3ml)	£547	
- 1.3kg≤ Birth weight ≤1.8kg (1 bottle 3ml and 1 bottle 1.5ml)	£829	
- 1.9kg≤ Birth weight ≤2.4kg (2 bottles 3ml)	£1,095	
- 2.5kg≤ Birth weight ≤3kg (2 bottles 3ml and 1 bottle 1.5ml)	£1,376	
- 3.1kg≤ Birth weight ≤3.6kg (3 bottles 3ml)	£1,642	
- 3.7kg ≤ Birth weight≤4.2kg (3 bottles 3ml and 1 bottle 1.5ml)	£1,924	
Total parental nutrition (per day, over 14 days of use)**	£48	Walter et al. (2012); inflated to 2018/19 prices [5]
Palivizumab (per day at 15ml/kg birth weight)	£435	British National Formulary online: NHS indicative price [4]
ROP surgery	£1,731	National Cost Collection Data Publication. National Schedule of NHS Costs 2018/19. HRG code BZ86C, elective care [3]
Neonatal surgery***	Various	HRG4+ 2017/18 Reference Costs Grouper; inflated to 2018/19 prices [6]

Confidential: For Review Only

ROP: retinopathy of prematurity;
*Surfactant use was reported from 3 separate fields from the datasets: Surfactant given today, Drugs given today and Surfactant given at resuscitation. The first two variables were recorded at the daily level, while the third variable was recorded at the episode level and therefore we defined its use for the first daily record of this episode;
**The use of total parental nutrition (TPN) was reported from 2 separate fields from the datasets: TPN given today and Drugs given today;
***Different types of neonatal surgery were identified from OPCS and ICD-10 codes using the HRG4+ 2017/18 Reference Costs Grouper Software.

Confidential: For Review Only

Table A2: Baseline characteristics of babies born between 27 and 31 weeks gestation with and without missing NNRD data on daily records or level of daily care provided – data are frequencies (percentages) unless otherwise stated

	Babies with missing daily records or level of care data (n=1,669)	Babies with complete information (n=28,173)	p value*
	n (%)	n (%)	
**Gestational age at birth**			
27 weeks	284 (17.0%)	3,296 (11.7%)	
28 weeks	304 (18.2%)	4,370 (15.5%)	
29 weeks	289 (17.3%)	5,036 (17.9%)	p<0.001
30 weeks	354 (21.2%)	6,625 (23.5%)	
31 weeks	435 (26.1%)	8,827 (31.3%)	
Missing	*3 (0.2%)*	*19 (0.1%)*	
**Gender of baby**			
Male	961 (57.6%)	15,363 (54.5%)	0.046
Female	704 (42.2%)	12,755 (45.3%)	
Missing	*4 (0.2%)*	*55 (0.2%)*	
**Number of fetus**			
Singleton birth	1,229 (73.6%)	20,555 (73.0%)	0.632
Multiple birth	438 (26.2%)	7,598 (27.0%)	
Missing	*2 (0.1%)*	*20 (0.1%)*	
**Birthweight (g) - mean(SD)**	1,282.6 (358.2)	1,330.2 (332.2)	p<0.001
Missing	*8 (0.5%)*	*82 (0.3%)*	
**Apgar score at 5min - mean(SD)**	8.1 (1.7)	8.1 (1.8)	0.030
Missing	*156 (9.4%)*	*2,877 (10.2%)*	
**Died in neonatal care**	22 (1.3%)	985 (3.5%)	p<0.001
Missing	*1 (0.1%)*	*0 (0.0%)*	

SD: standard deviation; *Continuous variables were tested by independent t-test, categorical variables by chi-square test

Confidential: For Review Only

Table A3: A comparison of gestational age at birth between very preterm births in the study cohort and those identified from national live births statistics for England for in 2016, 2017 and 2018

Year<br>Gestational weeks	Study cohort	National data for England from Office for National Statistics (ONS)	p-value*
**2016**			
27	704 (12.6%)	768 (12.6%)	
28	883 (15.8%)	950 (15.5%)	
29	1,015 (18.2%)	1,101 (18.0%)	0.977
30	1,282 (23.0%)	1,412 (23.1%)	
31	1,689 (30.3%)	1,881 (30.8%)	
**2017**			
27	638 (11.7%)	690 (11.6%)	
28	860 (15.7%)	949 (16.0%)	
29	991 (18.1%)	1,078 (18.2%)	0.987
30	1,305 (23.9%)	1,427 (24.1%)	
31	1,670 (30.6%)	1,787 (30.1%)	
**2018**			
27	476 (11.0%)	696 (12.0%)	
28	620 (14.3%)	888 (15.3%)	
29	760 (17.5%)	1,039 (17.9%)	0.151
30	1,035 (23.8%)	1,358 (23.4%)	
31	1,448 (33.3%)	1,835 (31.6%)	

*chi-square test of proportions

Source: Office for National Statistics; births extracted from a dataset containing birth registrations. 2016 and 2017 figures exclude births where mothers' usual residence was outside of England. 2018 figures include births where mothers' usual residence was in England and Wales. All figures were based on babies born in the calendar year.

Confidential: For Review Only

Table A4: Counts of hospital transfers, surgeries and days receiving other non-routine procedures during neonatal admissions for very preterm babies in England for the period 2014-2018

	27 weeks gestation n=3,296	28 weeks gestation n=4,370	29 weeks gestation n=5,036	30 weeks gestation n=6,625	31 weeks gestation n=8,827
**Hospital transfer within 24 hours of birth***					
Number of transfers	287	330	293	286	311
Number (%) of babies receiving	276 (8.4%)	323 (7.4%)	286 (5.7%)	282 (4.3%)	307 (3.5%)
**Hospital transfer after 24 hours of birth***					
Number of transfers	872	836	713	659	571
Number (%) of babies receiving	654 (19.8%)	665 (15.2%)	593 (11.8%)	588 (8.9%)	523 (5.9%)
**Nitric oxide**					
Number of days of care	1,333	1,363	953	918	660
Number (%) of babies receiving	425 (12.9%)	467 (10.7%)	360 (7.1%)	419 (6.3%)	326 (3.7%)
**Surfactant replacement**					
Number of days of care	5,932	6,561	5,525	4,201	3,667
Number (%) of babies receiving	2,743 (83.2%)	3,258 (74.6%)	3,023 (60.0%)	2,677 (40.4%)	2,386 (27.0%)
**TPN**					
Number of days of care (over 14 days' use)	20,182	17,919	11,200	7,525	5,074
Number (%) of babies receiving	1,391 (42.2%)	1,411 (32.3%)	990 (19.7%)	692 (10.4%)	515 (5.8%)
**Palivizumab**					
Number of days of care	90	103	52	31	14
Number (%) of babies receiving	77 (2.3%)	68 (1.6%)	38 (0.8%)	26 (0.4%)	12 (0.1%)
**ROP surgery**					
Number of days of care	116	53	36	34	15
Number (%) of babies receiving	91 (2.8%)	45 (1.0%)	32 (0.6%)	30 (0.5%)	15 (0.2%)
**Neonatal surgery***					
Number of surgeries	291	283	203	175	169
Number (%) of babies receiving	255 (7.7%)	266 (6.1%)	185 (3.7%)	162 (2.4%)	161 (1.8%)

TPN: total parenteral nutrition, ROP: retinopathy or prematurity,*For these items, numbers recorded are reported. For remaining items total number of days receiving treatment are reported.

Confidential: For Review Only

Table A5: Mean (SE) level of care cost per baby (2018/19 UK £) for very preterm babies in England over the period 2014-2018 using multiple imputation (n = 29,842)

	27 weeks gestation (n = 3,580)	28 weeks gestation (n = 4,674)	29 weeks gestation (n = 5,325)	30 weeks gestation (n = 6,979)	31 weeks gestation (n = 9,284)
**Resource use item**	**Mean (SD) cost per baby**	**Mean (SD) cost per baby**	**Mean (SD) cost per baby**	**Mean (SD) cost per baby**	**Mean (SD) cost per baby**
**Level of daily care**					
Intensive care	£27,814 (£415)	£20,854 (£311)	£14,468 (£215)	£8,500 (£137)	£5,082 (£107)
High-dependency care	£27,169 (£385)	£19,164 (£288)	£11,561 (£202)	£7,823 (£135)	£5,115 (£81)
Special care without carer	£19,720 (£200)	£21,080 (£164)	£21,380 (£128)	£19,612 (£98)	£16,235 (£75)
Special care with carer	£776 (£22)	£840 (£23)	£828 (£20)	£842 (£18)	£835 (£15)
Normal care	£0 (£0)	£0.44 (£0.44)	£0 (£0)	£0.22 (£0.22)	£0.50 (£0.40)
**Total level of care costs**	**£75,479 (£604)**	**£61,938 (£462)**	**£48,236 (£331)**	**£36,778 (£225)**	**£27,269 (£160)**

SE: standard error

Confidential: For Review Only

References

1. White, I.R., Royston, P., and Wood, A.M. Multiple imputation using chained equations: Issues and guidance for practice. Stat Med, 2011. **30**(4): 377-99.

2. Little, R.J. and Rubin, D.B. Statistical Analysis with Missing Data. 2nd ed. Wiley Series in Probability and Statistics. 2002, Hoboken, NJ: Wiley.

3. NHS England. (2019). National Cost Collection Data Publication. National Schedule of NHS Costs 2018/2019. Available from https://www.england.nhs.uk/publication/2018-19-national-cost-collection-data-publication/. [Accessed 12th November 2022].

4. Joint Formulary Committee. British National Formulary (online). Available from http://www.medicinescomplete.com. [Accessed 12th November 2022].

5. Walter, E., Liu, F.X., Maton, P., Storme, T., Perrinet, M., von Delft, O., Puntis, J., Hartigan, D., Dragosits, A., and Sondhi, S. Cost analysis of neonatal and pediatric parenteral nutrition in Europe: a multi-country study. Eur J Clin Nutr, 2012. **66**(5): 639-44.

6. NHS Digital. HRG4 2017/18 Local Payment Grouper. Available from https://digital.nhs.uk/services/national-casemix-office/downloads-groupers-and-tools/payment-hrg4-2017-18-local-payment-grouper. [Accessed 12 November 2022].

Confidential: For Review Only

List of participating members of the UK Neonatal Collaborative in the OPTI-PREM Study

Institution	Neonatal Network	Clinical Lead
Airedale General Hospital	Yorkshire Neonatal Network	Dr Matthew Babirecki
Barnet Hospital	London - North Central Neonatal Network	Dr Tim Wickham
Barnsley District General Hospital	North Trent Neonatal Network	Dr Sanaa Hamdan
Basildon Hospital	London - North East and North Middlesex Neonatal Network	Dr Aashish Gupta
Basingstoke & North Hampshire Hospital	Thames Valley & Wessex Neonatal Networks	Dr Ruth Wigfield
City Hospital, Birmingham	Midlands South West Newborn Network	Dr Julie Nycyk
Broomfield Hospital	Norfolk, Suffolk & Cambridgeshire Neonatal Network	Dr Ahmed Hassan
Calderdale Royal Hospital	Yorkshire Neonatal Network	Dr Karin Schwarz
Chesterfield & North Derbyshire Royal Hospital	North Trent Neonatal Network	Dr Aiwyne Foo
Colchester General Hospital	Norfolk, Suffolk & Cambridgeshire Neonatal Network	Dr Aravind Shastri
Countess of Chester Hospital	Cheshire and Merseyside Neonatal Network	Dr Stephen Brearey
Croydon University Hospital	London - South West Neonatal Network	Dr John Chang
Diana Princess of Wales Hospital	North Trent Neonatal Network	Dr Pauline Adiotomre
Doncaster Royal Infirmary	North Trent Neonatal Network	Dr Jamal S Ahmed
Dorset County Hospital	Thames Valley & Wessex Neonatal Networks	Dr Abby Deketelaere
East Surrey Hospital	South East Coast Neonatal ODN	Dr K Abdul Khader
Great Western Hospital	South West Region	Dr Stanley Zengeya
Hillingdon Hospital	London - North West Neonatal Network	Dr Tristan Bate
Hinchingbrooke Hospital	Norfolk, Suffolk & Cambridgeshire Neonatal Network	Dr Hilary Dixon
Ipswich Hospital	Norfolk, Suffolk & Cambridgeshire Neonatal Network	Dr Matthew James
James Paget Hospital	Norfolk, Suffolk & Cambridgeshire Neonatal Network	Dr Ambadkar
Kettering General Hospital	Midlands Central Neonatal Network	Dr Patty Rao
King's Mill Hospital	Trent Perinatal Network	Dr Dhaval Dave
Kingston Hospital	London - South West Neonatal Network	Dr Vinay Pai
Leighton Hospital	Cheshire and Merseyside Neonatal Network	Dr Jayachandran
Lincoln County Hospital	Trent Perinatal Network	Dr Kollipara
Lister Hospital	Beds-Herts Neonatal Network	Dr J Kefas
Macclesfield District General Hospital	Cheshire and Merseyside Neonatal Network	Dr Gail Whitehead
Manor Hospital	Staffordshire, Shropshire & Black Country Newborn & Maternity Network	Dr Krishnamurthy
Milton Keynes Foundation Trust Hospital	Thames Valley & Wessex Neonatal Networks	Dr I Misra
Newham General Hospital	London - North East and North Middlesex Neonatal Network	Dr Imdad Ali
North Middlesex University Hospital	London - North East and North Middlesex Neonatal Network	Dr Lesley Alsford
North Tyneside General Hospital	Northern Neonatal Network	Vivien Spencer
Northampton General Hospital	Midlands Central Neonatal Network	Dr Subodh Gupta
Northwick Park Hospital	London - North West Neonatal Network	Dr Richard Nicholl
Ormskirk District General Hospital	Cheshire and Merseyside Neonatal Network	Dr Tim McBride
Peterborough City Hospital	Norfolk, Suffolk & Cambridgeshire Neonatal Network	Dr Katharine McDevitt
Pinderfields General Hospital	Yorkshire Neonatal Network	Dr David Gibson
Poole Hospital NHS Foundation Trust	Thames Valley & Wessex Neonatal Networks	Prof Minesh Khashu
Princess Alexandra Hospital	Norfolk, Suffolk & Cambridgeshire Neonatal Network	Dr Caitlin Toh
Queen Elizabeth Hospital, King's Lynn	Norfolk, Suffolk & Cambridgeshire Neonatal Network	Dr Glynis Rewitzky
Queen Elizabeth Hospital, Woolwich	London - South East Neonatal Network	Dr Olutoyin Banjoko
Queen's Hospital, Romford	London - North East and North Middlesex Neonatal Network	Dr Wilson Lopez

Confidential: For Review Only

Rotherham District General Hospital	North Trent Neonatal Network	Dr Shameel Mattara
Royal Albert Edward Infirmary	Greater Manchester Neonatal Network	Dr Christos Zipitis
Royal Berkshire Hospital	Thames Valley & Wessex Neonatal Networks	Dr Peter De Halpert
Royal Cornwall Hospital	South West Region	Dr Paul Munyard
Royal Derby Hospital	Trent Perinatal Network	Dr John McIntyre
Royal Devon & Exeter Hospital	South West Region	Dr David Bartle
Royal Hampshire County Hospital	Thames Valley & Wessex Neonatal Networks	Dr Katie Yallop
Royal Lancaster Infirmary	Lancashire and South Cumbria Neonatal Network	Dr Joanne Fedee
Royal Oldham Hospital	Greater Manchester Neonatal Network	Dr Natasha Maddock
Royal Shrewsbury Hospital	Staffordshire, Shropshire & Black Country Newborn & Maternity Network	Dr Deshpande
Royal United Hospital	South West Region	Dr Stephen Jones
Russells Hall Hospital	Staffordshire, Shropshire & Black Country Newborn & Maternity Network	Dr Mahadevan
Salisbury District Hospital	Thames Valley & Wessex Neonatal Networks	Dr Nick Brown
Scunthorpe General Hospital	North Trent Neonatal Network	Dr Pauline Adiotomre
Southend Hospital	London - North East and North Middlesex Neonatal Network	Dr Arfa Khan
St Helier Hospital	London - South West Neonatal Network	Dr Salim Yasin
St Mary's Hospital, IOW	Thames Valley & Wessex Neonatal Networks	Dr Sian Butterworth
St Mary's Hospital, London	London - North West Neonatal Network	Dr Sunit Godambe
St Richard's Hospital	Thames Valley & Wessex Neonatal Networks	Dr Nick Brennan
Stepping Hill Hospital	Greater Manchester Neonatal Network	Dr Carrie Heal
Stoke Mandeville Hospital	Thames Valley & Wessex Neonatal Networks	Dr Sanjay Salgia
Tameside General Hospital	Greater Manchester Neonatal Network	Dr Jacqeline Birch
Taunton & Somerset Hospital	South West Region	Dr Chris Knight
Tunbridge Wells Hospital	South East Coast Neonatal ODN	Dr Hamudi Kisat
University Hospital Lewisham	London - South East Neonatal Network	Dr Jauro Kuna
University Hospital of North Durham	Northern Neonatal Network	Dr Mehdi Garbash
University Hospital of South Manchester	Greater Manchester Neonatal Network	Dr Gopi Vemuri
Victoria Hospital, Blackpool	Lancashire and South Cumbria Neonatal Network	Dr Chris Rawlingson
Warrington Hospital	Cheshire and Merseyside Neonatal Network	Dr Delyth Webb
Watford General Hospital	Beds-Herts Neonatal Network	Dr Sankara Narayanan
West Suffolk Hospital	Norfolk, Suffolk & Cambridgeshire Neonatal Network	Dr Ian Evans
Wexham Park Hospital	Thames Valley & Wessex Neonatal Networks	Dr Rekha Sanghavi
Whipps Cross University Hospital	London - North East and North Middlesex Neonatal Network	Dr Caroline Sullivan
Whiston Hospital	Cheshire and Merseyside Neonatal Network	Dr Rosaline Garr
Whittington Hospital	London - North Central Neonatal Network	Dr Wynne Leith
Worcestershire Royal Hospital	Midlands South West Newborn Network	Dr Andrew Gallagher
York District Hospital	Yorkshire Neonatal Network	Dr Guy Millman
Gloucestershire Royal Hospital	South West Region	Dr Simon Pirie
Arrowe Park Hospital	Cheshire and Merseyside Neonatal Network	Dr Anand Kamalanathan
Birmingham Heartlands Hospital	Midlands South West Newborn Network	Dr Phil Simmons
Birmingham Women's Hospital	Midlands South West Newborn Network	Dr Anju Singh
Bradford Royal Infirmary	Yorkshire Neonatal Network	Dr Sunita Seal
Chelsea & Westminster Hospital	London - North West Neonatal Network	Dr Mark Thomas
Derriford Hospital	South West Region	Dr Alex Allwood
Guy's & St Thomas' Hospital	London - South East Neonatal Network	Dr Timothy Watts
Homerton Hospital	London - North East and North Middlesex Neonatal Network	Dr Narendra Aladangady
hull royal infirmary	Yorkshire Neonatal Network	Dr Hassan Gaili
James Cook University Hospital	Northern Neonatal Network	Dr M Lal
King's College Hospital	London - South East Neonatal Network	Dr Ann Hickey
Lancashire Women & Newborn Centre	Lancashire and South Cumbria Neonatal Network	Dr Meera Lama
Leeds Neonatal Service	Yorkshire Neonatal Network	Dr Lawrence Miall
Leicester Neonatal Service	Midlands Central Neonatal Network	Dr Jonathan Cusack

Confidential: For Review Only

Liverpool Women's Hospital	Cheshire and Merseyside Neonatal Network	Dr Bill Yoxall
Luton & Dunstable Hospital	Beds-Herts Neonatal Network	Dr Jennifer Birch
Medway Maritime Hospital	South East Coast Neonatal ODN	Dr Aung Soe
New Cross Hospital	Staffordshire, Shropshire & Black Country Newborn & Maternity Network	Dr Tilly Pillay
Norfolk & Norwich University Hospital	Norfolk, Suffolk & Cambridgeshire Neonatal Network	Dr Mark Dyke
Nottingham City Hospital	Trent Perinatal Network	Dr Steven Wardle
Nottingham University Hospital (QMC)	Trent Perinatal Network	Dr Steven Wardle
Oxford University Hospitals, John Radcliffe Hospital	Thames Valley & Wessex Neonatal Networks	Dr Eleri Adams
Princess Anne Hospital	Thames Valley & Wessex Neonatal Networks	Dr Mike Hall
Queen Alexandra Hospital	Thames Valley & Wessex Neonatal Networks	Dr Charlotte Groves
Queen Charlotte's Hospital	London - North West Neonatal Network	Dr Sunit Godambe
Rosie Maternity Hospital, Addenbrookes	Norfolk, Suffolk & Cambridgeshire Neonatal Network	Dr Angela D'Amore
Royal Bolton Hospital	Greater Manchester Neonatal Network	Dr Paul Settle
Royal Preston Hospital	Lancashire and South Cumbria Neonatal Network	Dr Richa Gupta
Royal Stoke University Hospital	Staffordshire, Shropshire & Black Country Newborn & Maternity Network	Dr Alison Moore
Royal Sussex County Hospital	South East Coast Neonatal ODN	Dr P Amess
Royal Victoria Infirmary	Northern Neonatal Network	Dr Alan Fenton
NORTH BRISTOL NHS TRUST (SOUTHMEAD)	South West Region	Dr Paul Mannix
St George's Hospital	London - South West Neonatal Network	Dr Charlotte Huddy
St Mary's Hospital, Manchester	Greater Manchester Neonatal Network	Dr Ngozi Edi-Osagie
St Michael's Hospital	South West Region	Dr Pamela Cairns
St Peter's Hospital	South East Coast Neonatal ODN	Dr Peter Reynolds
Sunderland Royal Hospital	Northern Neonatal Network	Dr Majd Abu-Harb
The Jessop Wing, Sheffield	North Trent Neonatal Network	Dr Simon Clark
The Royal London Hospital - Constance Green	London - North East and North Middlesex Neonatal Network	Dr Vadivelam Murthy
University College Hospital	London - North Central Neonatal Network	Dr Giles Kendall
University Hospital Coventry	Midlands Central Neonatal Network	Dr Kate Blake
University Hospital of North Tees	Northern Neonatal Network	Dr Hari Kumar
William Harvey Hospital	South East Coast Neonatal ODN	Dr Vimal Vasu

Confidential: For Review Only

BMJ Paediatrics Open

Neonatal health care costs of very preterm babies in England: a retrospective analysis of a national birth cohort

Journal:	*BMJ Paediatrics Open*
Manuscript ID	bmjpo-2022-001818.R2
Article Type:	Original research
Date Submitted by the Author:	05-Apr-2023

Complete List of Authors:	Yang, Miaoqing; University of Oxford, Nuffield Department of Population Health<br>Campbell, Helen; University of Oxford, Nuffield Department of Population Health<br>Pillay, Thillagavathie; University Hospitals of Leicester NHS Trust, Neonatology; University of Wolverhampton Faculty of Science and Engineering, School of Medicine and Clinical Practice<br>Boyle, Elaine M.; University of Leicester, Health Sciences<br>Modi, Neena; Imperial College London, Neonatal Medicine<br>Rivero-Arias, Oliver; University of Oxford, Nuffield Department of Population Health

Keywords:	Health services research, Neonatology

SCHOLARONE™
Manuscripts

I, the Submitting Author has the right to grant and does grant on behalf of all authors of the Work (as defined in the below author licence), an exclusive licence and/or a non-exclusive licence for contributions from authors who are: i) UK Crown employees; ii) where BMJ has agreed a CC-BY licence shall apply, and/or iii) in accordance with the terms applicable for US Federal Government officers or employees acting as part of their official duties; on a worldwide, perpetual, irrevocable, royalty-free basis to BMJ Publishing Group Ltd ("BMJ") its licensees and where the relevant Journal is co-owned by BMJ to the co-owners of the Journal, to publish the Work in this journal and any other BMJ products and to exploit all rights, as set out in our licence.

The Submitting Author accepts and understands that any supply made under these terms is made by BMJ to the Submitting Author unless you are acting as an employee on behalf of your employer or a postgraduate student of an affiliated institution which is paying any applicable article publishing charge ("APC") for Open Access articles. Where the Submitting Author wishes to make the Work available on an Open Access basis (and intends to pay the relevant APC), the terms of reuse of such Open Access shall be governed by a Creative Commons licence – details of these licences and which Creative Commons licence will apply to this Work are set out in our licence referred to above.

Other than as permitted in any relevant BMJ Author's Self Archiving Policies, I confirm this Work has not been accepted for publication elsewhere, is not being considered for publication elsewhere and does not duplicate material already published. I confirm all authors consent to publication of this Work and authorise the granting of this licence.

Confidential: For Review Only

Neonatal health care costs of very preterm babies in England: a retrospective analysis of a national birth cohort

Miaoqing Yang[1,2], Helen Campbell[1], Thillagavathie Pillay[3,4], Elaine Boyle[5], Neena Modi[6], Oliver Rivero-Arias[1]

Confidential: For Review Only

Running title: Neonatal costs of very preterm babies in England

[1] National Perinatal Epidemiology Unit (NPEU), Nuffield Department of Population Health, University of Oxford, Oxford, UK.
[2] Centre for Guidelines, National Institute for Health and Care Excellence, London, UK.
[3] Faculty of Science and Engineering, University of Wolverhampton, Wolverhampton, UK.
[4] University Hospitals Leicester NHS Trust, Department of Neonatology, Leicester Royal Infirmary, Leicester, UK
[5] Department of Population Health Sciences, University of Leicester, Leicester, UK.
[6] Section of Neonatal Medicine, School of Public Health, Chelsea and Westminster Hospital Campus, Imperial College London, London, UK

Corresponding author
Associate Professor Oliver Rivero-Arias
National Perinatal Epidemiology Unit (NPEU)
Nuffield Department of Population Health
Old Road Campus
University of Oxford
OX3 7LF
Email: oliver.rivero@npeu.ox.ac.uk

Abstract (244 words)

Objectives: Babies born between 27^{+0} and 31^{+6} weeks of gestation represent the largest group of very preterm babies requiring NHS care, however up-to-date cost figures for the UK are not currently available. This study estimates neonatal costs to hospital discharge for this group of very preterm babies in England.

Design: Retrospective analysis of resource use data recorded within the National Neonatal Research Database (NNRD).

Setting: Neonatal units in England.

Patients: Babies born between 27^{+0} and 31^{+6} weeks of gestation in England and discharged from a neonatal unit between 2014 and 2018.

Main outcome measures: Days receiving different levels of neonatal care were costed, along with other specialised clinical activities. Mean resource use and costs per baby are presented by gestational age at birth, along with total costs for the cohort.

Results: Based upon data for 28,154 very preterm babies, the annual total costs of neonatal care were estimated to be £262 million, with 95% of costs attributable to routine daily care provided by units. The mean (SD) total cost per baby of this routine care varied by gestational age at birth; £75,594 (£34,874) at 27 weeks as compared with £27,401 (£14,947) at 31 weeks.

Conclusions: Neonatal healthcare costs for very preterm babies vary substantially by gestational age at birth. The findings presented here are a useful resource to stakeholders including NHS managers, clinicians, researchers, and policy makers.

Confidential: For Review Only

What is already known on this topic

Existing cost estimates for very preterm care in England are now over a decade old and may not reflect modern care practices.

What this study adds

This study has generated current cost estimates at the level of the individual baby and for the cohort as a whole in England and confirmed the previously reported inverse relationship between healthcare costs and gestational age at birth.

How this study might affect research, practice or policy

The outputs from this work provide a valuable resource for research assessing the economic implications of interventions to prevent preterm birth, the provision of care for preterm babies, as well as helping inform NHS resource allocation decisions.

Confidential: For Review Only

Introduction

Babies born between 27^{+0} and 31^{+6} weeks of gestation (hereafter called 'born at 27–31 weeks') represent the largest group of very preterm babies requiring NHS care.[1] These babies also account for about 12% of all viable preterm babies born in England and usually require admission to a neonatal unit.[1]

Previous work in the UK and elsewhere has attempted to estimate the healthcare and societal cost of preterm birth.[2-7] However, there is marked variability in reported cost estimates, due to differences in study perspectives, included babies, data sources and methods used to assign costs. A number of studies have reported cost estimates associated with the initial period of hospitalisation for babies born at 27-31 weeks.[2 3 6] Whilst these studies have shown the costs of neonatal care for very preterm babies to be inversely related to gestational age at birth, estimates for the UK are now over a decade old and there is a need for new analyses. As part of the OPTI-PREM suite of studies aimed at optimising neonatal service provision for very preterm babies in England, we conducted a retrospective cohort study to describe the levels of neonatal care, key specialist procedures, and healthcare costs attributable to the management of these babies.[1] The analysis makes use of healthcare resource use data routinely collated within the National Neonatal Research Database (NNRD) on babies admitted to all neonatal units in England.

Methods

Study design and population

For this retrospective analysis, we included all admissions to neonatal units in England for babies born at 27-31 weeks and discharged or died between January 2014 and December 2018. Admissions were identifiable through the NNRD, which was created in 2007 to support activities including audit, evaluations, and clinical, health services and policy research, and is maintained at the Neonatal Data Analysis Unit (NDAU) at Imperial College London [https://www.imperial.ac.uk/neonatal-data-analysis-unit/]. All NHS neonatal units in England, Scotland, Wales, and the Isle of Man use their Electronic Patient Record (EPR) supplier systems to routinely submit detailed information to the NNRD on the

Confidential: For Review Only

clinical care they provide to babies. Submitted data are quality-assured and curated to a research standard. Data comprise demographics, diagnoses, health outcomes, and daily interventions (including the level of care, namely intensive, high dependency, special, or normal, provided each day) and treatments administered during the inpatient episode.

Data were extracted from the NNRD on 5th October 2020. Daily intervention data and treatment episode data were received in two separate datasets and were linked by means of participant identification number. Data were imported into Stata software and manipulated such that for each baby, a daily intervention record (which included the level of care provided) was available for each day of the admission episode. Babies missing daily intervention records or for whom daily records were available but level of care data had not been recorded, were excluded from the primary analysis.

Resource use and costs

The cost analysis was conducted from the perspective of the National Health Service (NHS) in England. To calculate the costs associated with the routine daily neonatal care received by each baby, we multiplied the number of days spent receiving each level of care, by level-specific national average bed day costs sourced from the 2018/19 National Schedule of NHS Costs.[8] Such costs are estimated by assigning each level of care a healthcare resource group (HRG) code. HRGs are groupings of clinically meaningful activities made primarily on the basis of diagnosis and procedure codes, and within the NHS, are the 'units' of health care for which providers receive payment. Costs assigned to each HRG are based upon nationally estimated tariffs developed to adequately cover the cost of providing high quality and cost-effective care. For this analysis the neonatal bed day costs used were: intensive care (HRG XA01Z), high dependency care (HRG XA02Z), special care without carer resident alongside baby (HRG XA03Z), special care with carer resident alongside baby (HRG XA04Z), and normal care (HRG XA05Z). Supplementary Table A1 shows these unit costs. Detailed information on the items included within each daily level of care cost are available from NHS England.[9]

Not all major clinical activities are included in these neonatal critical care HRGs. After in-depth discussions with clinical experts, we identified five high cost non-routine procedures captured within the NNRD, that were considered to be important in this particular population. These were nitric oxide,

Confidential: For Review Only

surfactant replacement, total parental nutrition (TPN) (over 14 days of use), palivizumab use and surgical care. Days receiving nitric oxide and TPN (beyond 14 days) were costed using *per diem* unit costs. Surfactant replacement and palivizumab were costed when given using unit costs specific to a baby's weight. Types of neonatal surgery were identified from their corresponding OPCS and ICD-10 codes for costing purposes and unit costs obtained from the 2018/19 National Schedule of NHS Costs. Unit costs for these non-routine procedures are detailed in supplementary Table A1. NNRD data for these five non-routine procedures were assumed to be complete, with no entry in the corresponding dataset fields taken as an indication that the procedure did not take place.

Statistical analysis

When describing neonatal and maternal characteristics, counts and proportions and means and standard deviations (SD) were used for categorical and continuous variables respectively. Analyses of resource use data and costs were performed by gestational age at birth. Means (SD) were used to summarise days provided at each level of care with associated 95% confidence interval following a Poisson distribution. Counts were made of the numbers of babies for whom non-routine procedures were recorded, along with the number of cases (for example surgeries) or days (for example for nitric oxide) for which such treatment was given. Total costs were reported for the cohort as a whole and again by gestational age at birth. A secondary analysis used multiple imputation to impute routine care costs at each level (intensive care, high dependency care etc.) for babies with missing daily record and level of care data (see supplementary Multiple Imputation Methods). Analyses were conducted using Stata MP.[10]

Patient and Public Involvement

An OPTI-PREM parent panel of mothers and fathers of babies born at 27-31 weeks in England was established with support from the national charity BLISS. The parent panel engaged in the study design and review of the funding protocol, development of parent information leaflets and neonatal unit posters. They attended team and study steering committee meetings, provided input at national stakeholder discussions, and contributed with the interpretation of final results, and our dissemination strategy.

Confidential: For Review Only

Results

Study population

Within the NNRD data extraction were 29,842 infants, with a total of 1,512,446 daily records and 46,746 episode records. Following the removal of infants with missing daily record information (n=1,292) and those with missing data on the level of daily care provided (n=377), a total of 28,173 babies (94% of the starting cohort of 29,842) remained and were included in the cost analysis (full details of the record matching process and data cleaning can be found in supplementary Figure A1). Table 1 summarises the characteristics of these babies and their mothers. A comparison between babies included in the analysis and those excluded due to missing data (n=1,669) was performed and showed the latter were more likely to have been born at earlier gestations (for example 17% versus 12% were born at 27 weeks; see supplementary Table A2). Despite this, birth statistics for the study cohort were still comparable to those of all very preterm babies born in England between 2016 to 2018 (data compiled by the Office for National Statistics; see supplementary Table A3).

Resource use

Table 2 shows the mean (SD) per baby duration (days) spent receiving each level of care according to gestational age at birth. Data show a consistent inverse relationship between gestational age at birth and the intensity of daily care provided. Figure 1 plots the mean durations along with mean overall length of stay on the neonatal unit and illustrates the longer durations of higher intensity care (and indeed overall neonatal care) provided to babies born at earlier gestations. For example, babies born at 27 weeks spent on average, 18.1 days (SD=15.7 days) receiving intensive care and 26.8 days (SD=22 days) receiving high-dependency care, while babies born at 31 weeks received an average of 3.33 days (SD=6.7 days) of intensive care and 5.1 days (SD=7.5 days) of high-dependency care. Also of note is that across all gestational age at birth groups, days spent receiving special care without a carer present accounted for the largest proportion of days spent on the neonatal unit.

Supplementary Table A4 presents the number of babies who underwent hospital transfers and received key non-routine procedures, again by gestational age at birth. Greater resource utilisation amongst infants born at earlier gestations can be seen.

Confidential: For Review Only

Costs

The mean (SD) cost per baby for the various levels of care and non-routine procedures are shown by gestational age at birth in Table 3 for the complete case analysis. As expected, and given the observations for resource use, costs can be seen to increase as gestational age at birth decreases. Mean (SD) total costsof routine care for a baby born at 27 weeks, for example, were, at £75,594 (£34,874), 2.8 times greater than costs for a baby born at 31 weeks (£27,401 (£14,974)). The final column in Table 3 shows that for the 2014-2018 cohort as a whole, just over 50% of total costs were attributable to the provision of intensive and high dependency care, with a further 42% coming from the provision of special care (without a carer). Further exploration of the composition of total costs in Figure 2 reveals variation by gestational age at birth. Almost 70% of total costs for babies born at 27 weeks were associated with the use of intensive- and high-dependency care, while the proportion decreased to only 36% for babies born at 31 weeks. The usage, and thus cost of special care (without a carer present) increases with increasing gestational age at birth. The results of the multiple imputation analysis for missing level of care costs (supplementary Table A5) were similar to the complete case analysis. Across all gestational at birth groups, non-routine procedures accounted for only a very small proportion of total costs.

The overall total cost estimated for the complete case cohort (n = 28,154) over the five years from 2014 to 2018 (£1.3billion in Table 3) suggests the annual costs of neonatal care for babies born between 27 and 31 gestational weeks and admitted to a neonatal unit in England to be around £262 million, with the provision of routine daily care on these units accounting for 96% (£252 million) of overall costs. Using the overall sample of 29,842 infants would increase the total cohort costs and the estimates of annual total costs shown in Table 3 from £1,309,192,377 to £1,394,308,706 and the annual total cost from £262 million to £279 million.

Confidential: For Review Only

Discussion

Main findings

Analyses at the level of the individual baby revealed the main cost drivers to be the daily neonatal care provided to support babies. Analyses by gestational age at birth provided further evidence of the previously reported inverse relationship between resource use and healthcare costs and the degree of prematurity, with care for a baby born at 27 weeks estimated to cost almost three times more than for a baby born at 31 weeks.[5 7] Our work also provides valuable information on the contribution of different resource components to overall costs and illustrates how the mix of intensive of care required by these babies varies with gestational age at birth.

A number of studies have previously estimated neonatal care costs for preterm babies across a range of gestational ages.[2-7] One of the most recent by Rios et al. in Canada[6] also used individual patient level resource use data from a neonatal database (the Canadian Neonatal Network Database) and included around 8,000 babies born between 27 and 31 weeks gestation. A comparison between the costs estimated for the babies in the Rios et al. study and the costs presented here (currency conversion from Can $ to UK £ made using Purchasing Power Parities) revealed the Canadian cost estimates to be consistently lower across all gestational age groups.[11] The most obvious explanation for these differences lies with the scope of the neonatal databases used by each study and the resulting implications for duration of neonatal unit stay. In general Rios et al. reported consistently lower mean lengths of stay for all gestional ages compared to our estimates due to their focus on intensive care units and the exclusion of the costs of care in lower dependency neonatal units. Thus the Rios et al. estimates do not reflect the true costs of healthcare provision for this group of babies.

In 2009, Mangham and colleagues used a decision analytic model to estimate neonatal costs for a hypothetical cohort of preterm babies born in England and Wales.[2] Model parameters were sourced from various cohort studies and the mean cost for a very pre-term baby (<33 weeks) was estimated to be £57,726 (95% CI: 28,779 to 94,868) (2006 GBP). With that analysis using a different methodology, reporting results for a wider gestational age range, and now being well over a decade old, comparisons with the estimates reported here are challenging. Whilst a number of more recent UK studies have been published on the costs of pre-term birth, these works have focussed specifically on births at other

Confidential: For Review Only

gestations (i.e. moderate and late preterm births or extremely preterm births).[2 4 12 13] One reassuring finding however is that the costs of neonatal care for very preterm births presented here fall between the costs reported for moderate / late preterm infants and for extremely preterm infants in the UK.

Strengths and limitations

This study makes a number of contributions to the published literature. Firstly, existing UK cost estimates for neonatal care following very preterm birth are over a decade old and so are unlikely to reflect current standards of practice.[2] This study provides up-to-date figures based on a large cohort of more than 28,000 babies who were discharged from or died within neonatal units across England between 2014 and 2018. Secondly, previous UK cost studies in the area have mainly relied on data synthesised from secondary sources to estimate costs. In utilising the NNRD, this study has been able to employ detailed and quality assured individual participant data derived from electronic patient records (EPR). Furthermore, the dataset offered a unique opportunity to capture near population-wide data (and thus costs) on day-to-day care provided across all neonatal units without imposing any additional burden on study participants. Thirdly, the richness of the dataset permitted us not only to cost routine daily care provided at differing levels of intensity, but also to consider the cost implications of a number of major non-routine procedures, which are not captured within the HRG codes for neonatal critical care. These detailed data will help inform the planning and provision of care for very pre-term babies. Finally, by generating up to date estimates of the mean resource use and costs of neonatal care for very preterm babies in England, this study provides valuable data of interest to a range of stakeholders, including NHS managers, clinicians providing care, researchers assessing the economic implications of therapies and interventions to prevent and treat preterm birth, and decision makers charged with implementing new policies and allocating resources. The estimates are also likely to be informative for other countries with levels of neonatal care provision that are similar to England.

A number of limitations must also be acknowledged. This study considered only healthcare costs associated with the initial period of hospitalisation, though the economic consequences of preterm birth extend over prolonged periods of time, in some cases over the lifetime of the individual.[2 13 14] Preterm birth can lead to additional healthcare as well as social care needs and special educational needs throughout childhood.[15 16] Further research is needed to adequately capture these wider costs

Confidential: For Review Only

accurately, including the economic costs to families while their preterm baby was hospitalised, and then throughout their lives. A further limitation is the exclusion from the analysis of babies with missing data on daily care provision, who were shown to have been born at earlier gestations than babies with complete data (supplementary Table A2). Whilst these babies accounted for only 6% of the initial NNRD cohort and data showed no differences in gestational age at birth between the babies with complete data and all very preterm births registered in England (supplementary Table A4), we observed small significant differences in baseline characteristics between babies with missing and complete data. We conducted a multiple imputation approach to understand the implications of these differences and our results suggested limited impact of the missing data in the overall cost results.

Conclusion

This study has generated up-to-date estimates of the costs of providing neonatal care to very preterm babies in England. Resource use and costs increase as gestational age decreases. The outputs from this work can be used to inform clinical and budgetary service planning and ensure the efficient allocation of health care resources. The estimates will also be of interest to countries with neonatal care provision similar to England.

Confidential: For Review Only

References

1. Pillay T, Modi N, Rivero-Arias O, et al. Optimising neonatal service provision for preterm babies born between 27 and 31 weeks gestation in England (OPTI-PREM), using national data, qualitative research and economic analysis: a study protocol. *BMJ Open* 2019;9(8):e029421. doi: 10.1136/bmjopen-2019-029421 [published Online First: 20190822]
2. Mangham LJ, Petrou S, Doyle LW, et al. The cost of preterm birth throughout childhood in England and Wales. *Pediatrics* 2009;123(2):e312-27. doi: 10.1542/peds.2008-1827
3. Korvenranta E, Linna M, Rautava L, et al. Hospital costs and quality of life during 4 years after very preterm birth. *Arch Pediatr Adolesc Med* 2010;164(7):657-63. doi: 10.1001/archpediatrics.2010.99
4. Khan KA, Petrou S, Dritsaki M, et al. Economic costs associated with moderate and late preterm birth: a prospective population-based study. *BJOG* 2015;122(11):1495-505. doi: 10.1111/1471-0528.13515 [published Online First: 20150722]
5. Petrou S, Yiu HH, Kwon J. Economic consequences of preterm birth: a systematic review of the recent literature (2009-2017). *Arch Dis Child* 2019;104(5):456-65. doi: 10.1136/archdischild-2018-315778 [published Online First: 20181109]
6. Rios JD, Shah PS, Beltempo M, et al. Costs of Neonatal Intensive Care for Canadian Infants with Preterm Birth. *J Pediatr* 2021;229:161-67 e12. doi: 10.1016/j.jpeds.2020.09.045 [published Online First: 20200923]
7. Rolnitsky A, Unger SL, Urbach DR, et al. Cost of neonatal intensive care for extremely preterm infants in Canada. *Transl Pediatr* 2021;10(6):1630-36. doi: 10.21037/tp-21-36
8. NHS England. National Cost Collection Data Publication. National Schedule of NHS Costs 2018/2019 2019 [Available from: https://www.england.nhs.uk/publication/2018-19-national-cost-collection-data-publication/ accessed 12th November 2022.
9. NHS England. Clinicians quick reference guide – Neonatal Critical Care HRGs 2016 & NCCMDS Codes: National Casemix Office; 2016 [Available from: https://www.networks.nhs.uk/nhs-networks/staffordshire-shropshire-and-black-country-newborn/documents/nhs-england-quick-reference-neonatal-critical-care-hrgs-2016-nccmds-codes accessed 2nd March 2023.
10. Stata Statistical Software Release 15 [program]. College Station, TX: StataCorp LP, 2015.
11. Eurostat-OECD. Methodological Manual on Purchasing Power Parities. Luxembourg: Publications Office of the European Union: OECD 2012.
12. Hinchliffe SR, Seaton SE, Lambert PC, et al. Modelling time to death or discharge in neonatal care: an application of competing risks. *Paediatr Perinat Epidemiol* 2013;27(4):426-33. doi: 10.1111/ppe.12053 [published Online First: 20130415]
13. Kim SW, Andronis L, Seppanen AV, et al. Economic costs at age five associated with very preterm birth: multinational European cohort study. *Pediatr Res* 2022;92(3):700-11. doi: 10.1038/s41390-021-01769-z [published Online First: 20211112]
14. Ni Y, Mendonca M, Baumann N, et al. Social Functioning in Adults Born Very Preterm: Individual Participant Meta-analysis. *Pediatrics* 2021;148(5) doi: 10.1542/peds.2021-051986 [published Online First: 20211026]
15. Alterman N, Johnson S, Carson C, et al. Gestational age at birth and child special educational needs: a UK representative birth cohort study. *Arch Dis Child* 2021;106(9):842-48. doi: 10.1136/archdischild-2020-320213 [published Online First: 20210122]
16. Alterman N, Johnson S, Carson C, et al. Gestational age at birth and academic attainment in primary and secondary school in England: Evidence from a national cohort study. *PloS one* 2022;17(8):e0271952. doi: 10.1371/journal.pone.0271952 [published Online First: 20220817]

For peer review only

Data availability statement

The National Neonatal Research Database can be accessed by making a request to the Neonatal Data Analysis Unit at Imperial College London through the Health Data Research UK Gateway (https://web.www.healthdatagateway.org/search?search=NNRD&datasetSort=latest&tab=Datasets).

Ethics statements

Patient consent for publication

Not required.

Ethics approval

Research ethics approval for the OPTI-PREM programme of work was obtained through the national Integrated Research Application System (IRAS, reference number 212 304 and research ethics committee reference number 17/NE/0800; North East – Tyne and Wear South).

Funding

This work is supported by the National Institute for Health Research, Health Services and Delivery Research Stream, project number 15/70/104 CRN accrual was approved by the NIHR for the period (1 August 2017 to 31 August 2018).

Competing interests

Authors declare no conflict of interests.

Contributors

ORA developed the study concept and aims, and developed the study proposal with MY, TP, EB, and NM. MY and ORA conducted the statistical analyses. MY, HC and ORA wrote the first draft of the manuscript. All other authors reviewed the draft and provided critical input. All authors approve the final version.

Acknowledgments

Confidential: For Review Only

To parents, patients and families participating in the OPTI-PREM study, and to the OPTI-PREM Parent Panel, for its support. The OPTI-PREM study is grateful to its NIHR Project Steering Committee: Andrew Ewer (chair), Stavros Petrou, Gillian Santorelli, Josie Anderson, David Loughton, Lisa Stanton, Karen Luyt, and to the National Neonatal Collaborative. We are indebted to all members of the UK Neonatal Collaborative that participated and made this study possible (see supplementary file for list of members).

The OPTI-PREM Study team: Elaine M Boyle, Neena Modi, Oliver Rivero-Arias, Bradley Manktelow, Sarah E Seaton, Natalie Armstrong, Miaoqing Yang, Abdul Qader T Ismail, Vasiliki Bountziouka, Caroline S Cupit, Alexis Paton, Victor L Banda, Elizabeth S Draper, Kelvin Dawson and Thillagavathie Pillay (Chief Investigator).

Confidential: For Review Only

Table 1: Baby and maternal characteristics of study cohort - data are frequencies (percentages) unless otherwise stated

	Cohort (n = 28,173)
	**n (%)**
Baby characteristics	
**Gestational age at birth**	
27 weeks	3,296 (11.7%)
28 weeks	4,370 (15.5%)
29 weeks	5,036 (17.9%)
30 weeks	6,625 (23.5%)
31 weeks	8,827 (31.3%)
Missing	*19 (0.1%)*
**Gender of baby**	
Male	15,363 (54.5%)
Female	12,755 (45.3%)
Missing	*55 (0.2%)*
**Number of fetuses**	
Singleton birth	20,555 (73.0%)
Multiple birth	7,598 (27.0%)
Missing	*20 (0.1%)*
**Birthweight (g) – mean (SD)**	1330.2 (332.2)
Missing	*82 (0.3%)*
**Apgar score at 5min – mean (SD)**	8.1 (1.8)
Missing	*2,877 (10.2%)*
**Died in neonatal care**	985 (3.5%)
Missing	*0 (0.0%)*
Maternal characteristics	
**Age (years) – mean (SD)**	30.7 (6.3)
Missing	*279 (1.0%)*
**Ethnicity**	
White	17,647 (62.6%)
Black	2,011 (7.1%)
Asian	3,144 (11.2%)
Mixed	433 (1.5%)
Other	483 (1.7%)
Missing	*4,455 (15.8%)*
**Diabetes**	2,378 (8.4%)
Missing	*10,736 (38.1%)*
**Hypertension**	3,506 (12.4%)
Missing	*10,460 (37.1%)*
**Infection**	3,029 (10.6%)
Missing	*10,595 (37.6%)*
**Mode of delivery**	
Vaginal-spontaneous	8,200 (29.1%)
Vaginal-instrumental	765 (2.7%)
Caesarean section	17,691 (62.3%)
Missing	*1,517 (5.4%)*

Confidential: For Review Only

**Quintiles of IMD**	
1st Q, least deprived	3,355 (11.9%)
2nd Q	3,761 (13.4%)
3rd Q	4,516 (16.0%)
4th Q	5,930 (21.1%)
5th Q, most deprived	8,057 (28.6%)
Missing	*2,554 (9.1%)*

SD: standard deviation; IMD: Index of Multiple Deprivation; Q: quintile

Confidential: For Review Only

Table 2: Summary of type and duration of daily care received by very preterm babies during neonatal unit admissions in England for the period 2014-2018 (n = 28,154)*

	Level of care provided (HRG Code)				
**Gestational age at birth**	**Intensive care (XA01Z)**	**High-dependency care (XA02Z)**	**Special care without carer (XA03Z)**	**Special care with carer (XA04Z)**	**Normal care (XA05Z)**
**27 weeks gestation (n=3,296)**					
Number of days of care	59,494	88,212	100,770	5,113	0
Number of babies receiving care	3,275	3,065	3,021	1,681	0
Mean (SD) duration of care (days)†	18.1 (15.7)	26.8 (22.0)	30.5 (18.0)	1.5 (2.5)	0.0 (0.0)
95% CI	17.9 to 18.2	26.6 to 27.0	30.4 to 30.7	1.5 to 1.6	0.0 to 0.0
Median (range) duration of care (days)†	14.0 (0.0 to 174.0)	23.0 (0.0 to 243.0)	30.5 (0.0 to 161.0)	1.0 (0.0 to 31.0)	0.0 (0.0 to 0.0)
**28 weeks gestation (n=4,370)**					
Number of days of care	59,440	83,399	141,630	7,345	4
Number of babies receiving care	4,298	4,050	4,097	2,268	1
Mean (SD) duration of care (days)†	13.6 (13.4)	19.1 (19.5)	32.4 (16.5)	1.7 (3.0)	0.0 (0.1)
95% CI	13.5 to 13.7	18.9 to 19.2	32.2 to 32.6	1.6 to 1.7	0.0 to 0.0
Median (range) duration of care (days)†	11.0 (0.0 to 246.0)	14.0 (0.0 to 203.0)	33.0 (0.0 to 127.0)	1.0 (0.0 to 44.0)	0.0 (0.0 to 4.0)
**29 weeks gestation (n=5,036)**					
Number of days of care	47,728	57,613	165,090	8,449	0
Number of babies receiving care	4,774	4,631	4,868	2,671	0
Mean (SD) duration of care (days)†	9.5 (10.2)	11.4 (14.1)	32.8 (13.8)	1.7 (3.0)	0.0 (0.0)
95% CI	9.4 to 9.6	11.4 to 11.5	32.6 to 32.9	1.6 to 1.7	0.0 to 0.0
Median (range) duration of care (days)†	8.0 (0.0 to 258.0)	7.0 (0.0 to 196.0)	33.0 (0.0 to 119.0)	1.0 (0.0 to 45.0)	0.0 (0.0 to 0.0)
**30 weeks gestation (n=6,625)**					
Number of days of care	36,599	51,479	199,283	11,428	3
Number of babies receiving care	5,196	5,724	6,471	3,582	1
Mean (SD) duration of care (days)†	5.5 (7.1)	7.8 (11.1)	30.1 (12.1)	1.7 (2.9)	0.0 (0.0)
95% CI	5.5 to 5.6	7.7 to 7.8	29.9 to 30.2	1.7 to 1.8	0.0 to 0.0
Median (range) duration of care (days)†	4.0 (0.0 to 133.0)	5.0 (0.0 to 213.0)	30.0 (0.0 to 115.0)	1.0 (0.0 to 37.0)	0.0 (0.0 to 3.0)
**31 weeks gestation (n=8,827)**					
Number of days of care	29,094	44,547	219,355	14,948	9
Number of babies receiving care	5,124	7,055	8,672	4,718	2
Mean (SD) duration of care (days)†	3.3 (6.7)	5.1 (7.5)	24.9 (10.5)	1.7 (2.8)	0.0 (0.1)
95% CI	3.3 to 3.3	5.0 to 5.1	24.7 to 25.0	1.7 to 1.7	0.0 to 0.0
Median (range) duration of care (days)†	1.0 (0.0 to 201.0)	3.0 (0.0 to 154.0)	24.0 (0.0 to 240.0)	1.0 (0.0 to 36.0)	0.0 (0.0 to 7.0)

SD: standard deviation, CI: parametric confidence interval using Poisson distribution, *19 babies had missing gestational age information, †Estimated across all babies within a gestational age group

Confidential: For Review Only

Table 3: Mean (SD) per baby and total neonatal care costs (2018/19 UK £) for very preterm babies in England over the period 2014-2018 using complete case analysis (n = 28,154)*

	27 weeks gestation (n = 3,296)	28 weeks gestation (n = 4,370)	29 weeks gestation (n = 5,036)	30 weeks gestation (n = 6,625)	31 weeks gestation (n = 8,827)	Total neonatal care costs (percentage) for 2014-2018
**Resource use item**	**Mean (SD) cost per baby**	**Mean (SD) cost per baby**	**Mean (SD) cost per baby**	**Mean (SD) cost per baby**	**Mean (SD) cost per baby**	
**Level of daily care**						
Intensive care	£27,690 (£24,039)	£20,865 (£20,473)	£14,548 (£15,603)	£8,482 (£10,887)	£5,062 (£10,235)	£356,747,496 (27.3%)
High-dependency care	£26,956 (£22,157)	£19,221 (£19,584)	£11,523 (£14,203)	£7,828 (£11,186)	£5,083 (£7,567)	£327,832,878 (25.0%)
Special care without carer	£20,187 (£11,880)	£21,407 (£10,921)	£21,659 (£9,110)	£19,875 (£8,019)	£16,423 (£6,939)	£546,285,433 (41.7%)
Special care with carer	£761 (£1,235)	£826 (£1,454)	£824 (£1,453)	£847 (£1,444)	£831 (£1,378)	£23,230,160 (1.8%)
Normal care	£0 (£0)	£0.47 (£31)	£0 (£0)	£0.23 (£19)	£0.52 (£40)	£8,224 (0.0%)
**Total level of care costs**	**£75,594 (£34,874)**	**£62,319 (£30,841)**	**£48,554 (£23,426)**	**£37,033 (£18,276)**	**£27,401 (£14,947)**	**£1,254,104,191 (95.8%)**
**Hospital transfers**	**£1,120 (£1,435)**	**£886 (£1,277)**	**£712 (£1,148)**	**£566 (£997)**	**£423 (£860)**	**£18,660,165 (1.4%)**
**Non-routine procedures**						
Nitric oxide	£105 (£557)	£81 (£436)	£49 (£354)	£36 (£312)	£19 (£148)	£1,356,929 (0.1%)
Surfactant replacement	£1,006 (£2,052)	£933 (£1,980)	£784 (£1,970)	£497 (£995)	£351 (£1,340)	£17,742,128 (1.4%)
TPN >14 days use	£930 (£2,203)	£609 (£1,838)	£316 (£1,398)	£147 (£883)	£71 (£550)	£8,919,898 (0.7%)
Palivizumab	£172 (£1,188)	£152 (£1,518)	£74 (£1,007)	£40 (£716)	£16 (£467)	£2,022,625 (0.2%)
ROP surgery	£61 (£398)	£21 (£223)	£12 (£165)	£8.9 (£138)	£2.9 (£71)	£439,674 (0.03%)
Neonatal surgery	£456 (£2,053)	£358 (£1,844)	£206 (£1,325)	£135 (£996)	£107 (£934)	£5,946,767 (0.5%)
**Total non-routine procedures costs**	**£2,730 (£4,594)**	**£2,153 (£4,228)**	**£1,442 (£3,333)**	**£864 (£2,143)**	**£567 (£1,955)**	**£36,428,021 (2.78%)**
Total neonatal care costs for 2014-2018						***£1,309,192,377***
Annual total neonatal costs						***£261,838,475***

SD: standard deviation, NHS: National Health Service, TPN: total parenteral nutrition, ROP: retinopathy of prematurity
*19 babies had missing gestational age information

Confidential: For Review Only

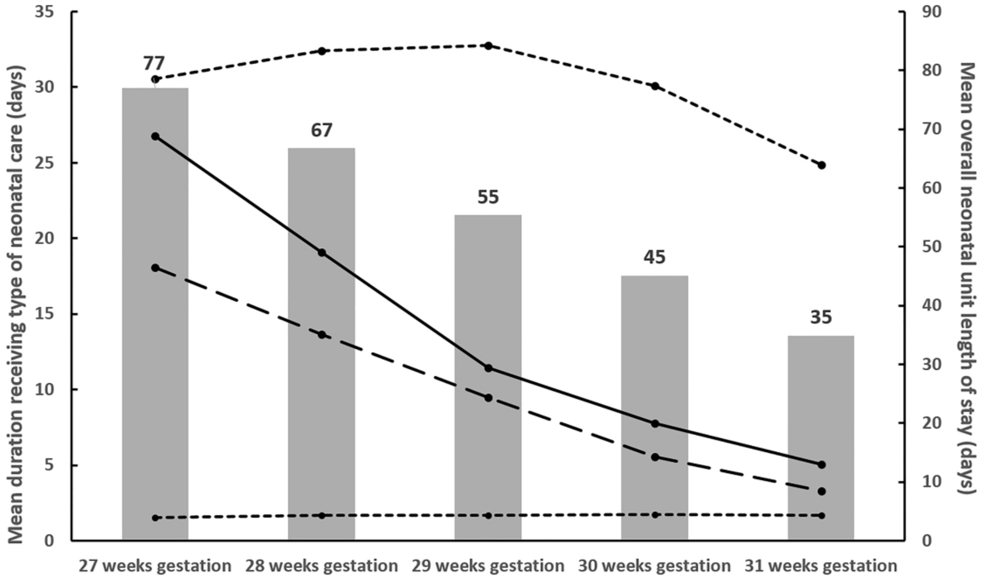

Figure 1 - Mean durations of different levels of daily care provided and overall length of stay per baby (in days) during neonatal unit admissions in England for the period 2014-2018. Data shown by gestational age at birth

463x275mm (300 x 300 DPI)

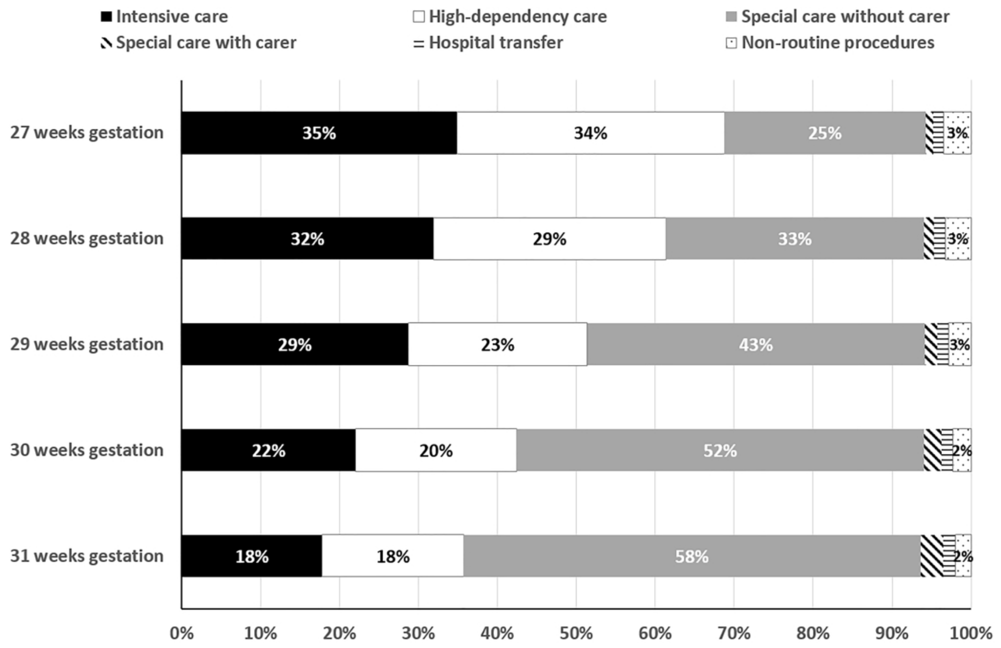

Figure 2 - Proportion of neonatal care cost attributable to different cost categories, by gestational age at birth

420x277mm (300 x 300 DPI)

Supplementary file

Multiple Imputation Methods

An imputation model was constructed, that included complete data on baseline baby and maternal characteristics, and the total cost of care provided at each level of care (intensive care, high-dependency care, special care without carer, special care with carer and normal care). We imputed costs for each level of care for babies with missing data (see Figure A1 below). Baseline characteristics included gestational age at birth, gender, number of fetuses, birthweight, neonatal death, maternal age and mode of delivery. All baseline covariates were subject to a small number of missing data that was imputed using conditional mean imputation prior to inclusion in the imputation model. A predictive mean matching estimation using chained equations with 50 imputations was implemented [1]. Mean estimates and estimates of standard errors were combined between imputed datasets using Rubin's rule [2] without any adjustment. Combined mean cost estimates and adjusted standard errors (SE) by gestational week across imputed datasets are shown to report the results of the multiple imputation analysis (Table A5 below).

Confidential: For Review Only

Figure A1: Flow of NNRD data available to estimate neonatal care costs for the very preterm babies discharged from neonatal units in England between 2014-2018.

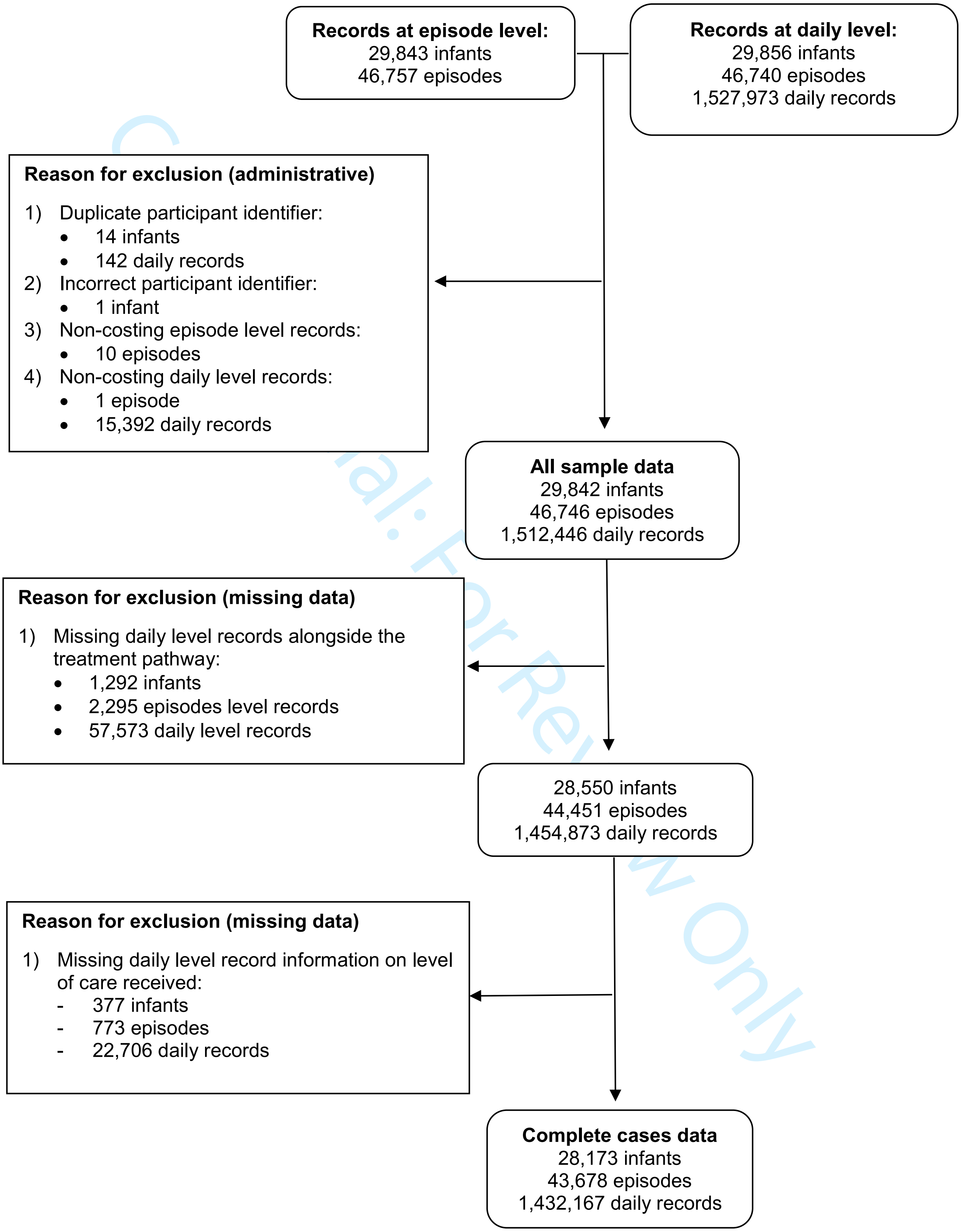

For Review Only

Table A1: Unit costs (expressed in 2018/19 UK pound sterling) used within the cost analysis

**Resource Use Item**	**Unit cost 2018/19 UK £**	**Source**
Neonatal unit inpatient bed days		
- intensive care level	£1,531	National Cost Collection Data Publication. National Schedule of NHS Costs 2018/19. HRG code XA01Z [3]
- high dependency care level	£1,007	National Cost Collection Data Publication. National Schedule of NHS Costs 2018/19. HRG code XA02Z [3]
- special care level, (carer not resident alongside baby)	£661	National Cost Collection Data Publication. National Schedule of NHS Costs 2018/19. HRG code XA03Z [3]
- special care level, (carer resident at cot-side and caring for baby)	£493	National Cost Collection Data Publication. National Schedule of NHS Costs 2018/19. HRG code XA04Z [3]
- normal care level	£514	National Cost Collection Data Publication. National Schedule of NHS Costs 2018/19. HRG code XA05Z [3]
Neonatal Critical Care, Transportation	£1,257	National Cost Collection Data Publication. National Schedule of NHS Costs 2018/19. HRG code XA06Z [3]
Inhaled nitric oxide (iNO) (per day)	£267	European iNO Registry for Liverpool Women's NHS Foundation Trust
*Surfactant replacement – poractant alfa (per day, by birth weight)**		
- Birth weight ≤0.6kg (1 bottle 1.5ml)	£282	British National Formulary online: NHS indicative price [4]
- 0.7kg≤ Birth weight ≤1.2kg (1 bottle 3ml)	£547	
- 1.3kg≤ Birth weight ≤1.8kg (1 bottle 3ml and 1 bottle 1.5ml)	£829	
- 1.9kg≤ Birth weight ≤2.4kg (2 bottles 3ml)	£1,095	
- 2.5kg≤ Birth weight ≤3kg (2 bottles 3ml and 1 bottle 1.5ml)	£1,376	
- 3.1kg≤ Birth weight ≤3.6kg (3 bottles 3ml)	£1,642	
- 3.7kg ≤ Birth weight≤4.2kg (3 bottles 3ml and 1 bottle 1.5ml)	£1,924	
Total parental nutrition (per day, over 14 days of use)**	£48	Walter et al. (2012); inflated to 2018/19 prices [5]
Palivizumab (per day at 15ml/kg birth weight)	£435	British National Formulary online: NHS indicative price [4]
ROP surgery	£1,731	National Cost Collection Data Publication. National Schedule of NHS Costs 2018/19. HRG code BZ86C, elective care [3]
Neonatal surgery***	Various	HRG4+ 2017/18 Reference Costs Grouper; inflated to 2018/19 prices [6]

Confidential: For Review Only

ROP: retinopathy of prematurity;
*Surfactant use was reported from 3 separate fields from the datasets: Surfactant given today, Drugs given today and Surfactant given at resuscitation. The first two variables were recorded at the daily level, while the third variable was recorded at the episode level and therefore we defined its use for the first daily record of this episode;
**The use of total parental nutrition (TPN) was reported from 2 separate fields from the datasets: TPN given today and Drugs given today;
***Different types of neonatal surgery were identified from OPCS and ICD-10 codes using the HRG4+ 2017/18 Reference Costs Grouper Software.

Confidential: For Review Only

Table A2: Baseline characteristics of babies born between 27 and 31 weeks gestation with and without missing NNRD data on daily records or level of daily care provided – data are frequencies (percentages) unless otherwise stated

	Babies with missing daily records or level of care data (n=1,669)	Babies with complete information (n=28,173)	p value*
	n (%)	n (%)	
**Gestational age at birth**			
27 weeks	284 (17.0%)	3,296 (11.7%)	
28 weeks	304 (18.2%)	4,370 (15.5%)	
29 weeks	289 (17.3%)	5,036 (17.9%)	p<0.001
30 weeks	354 (21.2%)	6,625 (23.5%)	
31 weeks	435 (26.1%)	8,827 (31.3%)	
Missing	*3 (0.2%)*	*19 (0.1%)*	
**Gender of baby**			
Male	961 (57.6%)	15,363 (54.5%)	0.046
Female	704 (42.2%)	12,755 (45.3%)	
Missing	*4 (0.2%)*	*55 (0.2%)*	
**Number of fetus**			
Singleton birth	1,229 (73.6%)	20,555 (73.0%)	0.632
Multiple birth	438 (26.2%)	7,598 (27.0%)	
Missing	*2 (0.1%)*	*20 (0.1%)*	
**Birthweight (g) - mean(SD)**	1,282.6 (358.2)	1,330.2 (332.2)	p<0.001
Missing	*8 (0.5%)*	*82 (0.3%)*	
**Apgar score at 5min - mean(SD)**	8.1 (1.7)	8.1 (1.8)	0.030
Missing	*156 (9.4%)*	*2,877 (10.2%)*	
**Died in neonatal care**	22 (1.3%)	985 (3.5%)	p<0.001
Missing	*1 (0.1%)*	*0 (0.0%)*	

SD: standard deviation; *Continuous variables were tested by independent t-test, categorical variables by chi-square test

Confidential: For Review Only

Table A3: A comparison of gestational age at birth between very preterm births in the study cohort and those identified from national live births statistics for England for in 2016, 2017 and 2018

Year<br>Gestational weeks	Study cohort	National data for England from Office for National Statistics (ONS)	p-value*
**2016**			
27	704 (12.6%)	768 (12.6%)	
28	883 (15.8%)	950 (15.5%)	
29	1,015 (18.2%)	1,101 (18.0%)	0.977
30	1,282 (23.0%)	1,412 (23.1%)	
31	1,689 (30.3%)	1,881 (30.8%)	
**2017**			
27	638 (11.7%)	690 (11.6%)	
28	860 (15.7%)	949 (16.0%)	
29	991 (18.1%)	1,078 (18.2%)	0.987
30	1,305 (23.9%)	1,427 (24.1%)	
31	1,670 (30.6%)	1,787 (30.1%)	
**2018**			
27	476 (11.0%)	696 (12.0%)	
28	620 (14.3%)	888 (15.3%)	
29	760 (17.5%)	1,039 (17.9%)	0.151
30	1,035 (23.8%)	1,358 (23.4%)	
31	1,448 (33.3%)	1,835 (31.6%)	

*chi-square test of proportions

Source: Office for National Statistics; births extracted from a dataset containing birth registrations. 2016 and 2017 figures exclude births where mothers' usual residence was outside of England. 2018 figures include births where mothers' usual residence was in England and Wales. All figures were based on babies born in the calendar year.

Confidential: For Review Only

Table A4: Counts of hospital transfers, surgeries and days receiving other non-routine procedures during neonatal admissions for very preterm babies in England for the period 2014-2018 (n = 28,154)*

	27 weeks gestation n=3,296	28 weeks gestation n=4,370	29 weeks gestation n=5,036	30 weeks gestation n=6,625	31 weeks gestation n=8,827
**Hospital transfer within 24 hours of birth**					
Number of transfers	287	330	293	286	311
Number (%) of babies receiving	276 (8.4%)	323 (7.4%)	286 (5.7%)	282 (4.3%)	307 (3.5%)
**Hospital transfer after 24 hours of birth**					
Number of transfers	872	836	713	659	571
Number (%) of babies receiving	654 (19.8%)	665 (15.2%)	593 (11.8%)	588 (8.9%)	523 (5.9%)
**Nitric oxide**					
Number of days of care	1,333	1,363	953	918	660
Number (%) of babies receiving	425 (12.9%)	467 (10.7%)	360 (7.1%)	419 (6.3%)	326 (3.7%)
**Surfactant replacement**					
Number of times given	5,932	6,561	5,525	4,201	3,667
Number (%) of babies receiving	2,743 (83.2%)	3,258 (74.6%)	3,023 (60.0%)	2,677 (40.4%)	2,386 (27.0%)
**TPN**					
Number of days of care (over 14 days' use)	20,182	17,919	11,200	7,525	5,074
Number (%) of babies receiving	1,391 (42.2%)	1,411 (32.3%)	990 (19.7%)	692 (10.4%)	515 (5.8%)
**Palivizumab**					
Number of times given	90	103	52	31	14
Number (%) of babies receiving	77 (2.3%)	68 (1.6%)	38 (0.8%)	26 (0.4%)	12 (0.1%)
**ROP surgery**					
Number of surgeries	116	53	36	34	15
Number (%) of babies receiving	91 (2.8%)	45 (1.0%)	32 (0.6%)	30 (0.5%)	15 (0.2%)
**Neonatal surgery**					
Number of surgeries	291	283	203	175	169
Number (%) of babies receiving	255 (7.7%)	266 (6.1%)	185 (3.7%)	162 (2.4%)	161 (1.8%)

TPN: total parenteral nutrition, ROP: retinopathy or prematurity.*19 babies had missing gestational age information.

Confidential: For Review Only

Table A5: Mean (SE) level of care cost per baby (2018/19 UK £) for very preterm babies in England over the period 2014-2018 using multiple imputation (n = 29,842)

	27 weeks gestation (n = 3,580)	28 weeks gestation (n = 4,674)	29 weeks gestation (n = 5,325)	30 weeks gestation (n = 6,979)	31 weeks gestation (n = 9,284)
**Resource use item**	**Mean (SE) cost per baby**	**Mean (SE) cost per baby**	**Mean (SE) cost per baby**	**Mean (SE) cost per baby**	**Mean (SE) cost per baby**
**Level of daily care**					
Intensive care	£27,845 (£436)	£20,883 (£309)	£14,512 (£217)	£8,445 (£133)	£5,097 (£108)
High-dependency care	£27,212 (£386)	£19,284 (£292)	£11,486 (£198)	£7,788 (£135)	£5,112 (£82)
Special care without carer	£20,254 (£201)	£21,506 (£164)	£21,712 (£127)	£19,883 (£98)	£16,434 (£75)
Special care with carer	£762 (£22)	£829 (£22)	£825 (£20)	£850 (£18)	£832 (£15)
Normal care	£0 (£0)	£0.44 (£0.44)	£0 (£0)	£0.24 (£0.24)	£0.52 (£0.42)
**Total level of care costs**	**£76,072 (£613)**	**£62,502 (£461)**	**£48,534 (£326)**	**£36,966 (£222)**	**£27,475 (£159)**

SE: standard error

Confidential: For Review Only

References

1. White, I.R., Royston, P., and Wood, A.M. Multiple imputation using chained equations: Issues and guidance for practice. Stat Med, 2011. **30**(4): 377-99.

2. Little, R.J. and Rubin, D.B. Statistical Analysis with Missing Data. 2nd ed. Wiley Series in Probability and Statistics. 2002, Hoboken, NJ: Wiley.

3. NHS England. (2019). National Cost Collection Data Publication. National Schedule of NHS Costs 2018/2019. Available from https://www.england.nhs.uk/publication/2018-19-national-cost-collection-data-publication/. [Accessed 12th November 2022].

4. Joint Formulary Committee. British National Formulary (online). Available from http://www.medicinescomplete.com. [Accessed 12th November 2022].

5. Walter, E., Liu, F.X., Maton, P., Storme, T., Perrinet, M., von Delft, O., Puntis, J., Hartigan, D., Dragosits, A., and Sondhi, S. Cost analysis of neonatal and pediatric parenteral nutrition in Europe: a multi-country study. Eur J Clin Nutr, 2012. **66**(5): 639-44.

6. NHS Digital. HRG4 2017/18 Local Payment Grouper. Available from https://digital.nhs.uk/services/national-casemix-office/downloads-groupers-and-tools/payment-hrg4-2017-18-local-payment-grouper. [Accessed 12 November 2022].

Confidential: For Review Only

List of participating members of the UK Neonatal Collaborative in the OPTI-PREM Study

Institution	Neonatal Network	Clinical Lead
Airedale General Hospital	Yorkshire Neonatal Network	Dr Matthew Babirecki
Barnet Hospital	London - North Central Neonatal Network	Dr Tim Wickham
Barnsley District General Hospital	North Trent Neonatal Network	Dr Sanaa Hamdan
Basildon Hospital	London - North East and North Middlesex Neonatal Network	Dr Aashish Gupta
Basingstoke & North Hampshire Hospital	Thames Valley & Wessex Neonatal Networks	Dr Ruth Wigfield
City Hospital, Birmingham	Midlands South West Newborn Network	Dr Julie Nycyk
Broomfield Hospital	Norfolk, Suffolk & Cambridgeshire Neonatal Network	Dr Ahmed Hassan
Calderdale Royal Hospital	Yorkshire Neonatal Network	Dr Karin Schwarz
Chesterfield & North Derbyshire Royal Hospital	North Trent Neonatal Network	Dr Aiwyne Foo
Colchester General Hospital	Norfolk, Suffolk & Cambridgeshire Neonatal Network	Dr Aravind Shastri
Countess of Chester Hospital	Cheshire and Merseyside Neonatal Network	Dr Stephen Brearey
Croydon University Hospital	London - South West Neonatal Network	Dr John Chang
Diana Princess of Wales Hospital	North Trent Neonatal Network	Dr Pauline Adiotomre
Doncaster Royal Infirmary	North Trent Neonatal Network	Dr Jamal S Ahmed
Dorset County Hospital	Thames Valley & Wessex Neonatal Networks	Dr Abby Deketelaere
East Surrey Hospital	South East Coast Neonatal ODN	Dr K Abdul Khader
Great Western Hospital	South West Region	Dr Stanley Zengeya
Hillingdon Hospital	London - North West Neonatal Network	Dr Tristan Bate
Hinchingbrooke Hospital	Norfolk, Suffolk & Cambridgeshire Neonatal Network	Dr Hilary Dixon
Ipswich Hospital	Norfolk, Suffolk & Cambridgeshire Neonatal Network	Dr Matthew James
James Paget Hospital	Norfolk, Suffolk & Cambridgeshire Neonatal Network	Dr Ambadkar
Kettering General Hospital	Midlands Central Neonatal Network	Dr Patty Rao
King's Mill Hospital	Trent Perinatal Network	Dr Dhaval Dave
Kingston Hospital	London - South West Neonatal Network	Dr Vinay Pai
Leighton Hospital	Cheshire and Merseyside Neonatal Network	Dr Jayachandran
Lincoln County Hospital	Trent Perinatal Network	Dr Kollipara
Lister Hospital	Beds-Herts Neonatal Network	Dr J Kefas
Macclesfield District General Hospital	Cheshire and Merseyside Neonatal Network	Dr Gail Whitehead
Manor Hospital	Staffordshire, Shropshire & Black Country Newborn & Maternity Network	Dr Krishnamurthy
Milton Keynes Foundation Trust Hospital	Thames Valley & Wessex Neonatal Networks	Dr I Misra
Newham General Hospital	London - North East and North Middlesex Neonatal Network	Dr Imdad Ali
North Middlesex University Hospital	London - North East and North Middlesex Neonatal Network	Dr Lesley Alsford
North Tyneside General Hospital	Northern Neonatal Network	Vivien Spencer
Northampton General Hospital	Midlands Central Neonatal Network	Dr Subodh Gupta
Northwick Park Hospital	London - North West Neonatal Network	Dr Richard Nicholl
Ormskirk District General Hospital	Cheshire and Merseyside Neonatal Network	Dr Tim McBride
Peterborough City Hospital	Norfolk, Suffolk & Cambridgeshire Neonatal Network	Dr Katharine McDevitt
Pinderfields General Hospital	Yorkshire Neonatal Network	Dr David Gibson
Poole Hospital NHS Foundation Trust	Thames Valley & Wessex Neonatal Networks	Prof Minesh Khashu
Princess Alexandra Hospital	Norfolk, Suffolk & Cambridgeshire Neonatal Network	Dr Caitlin Toh
Queen Elizabeth Hospital, King's Lynn	Norfolk, Suffolk & Cambridgeshire Neonatal Network	Dr Glynis Rewitzky
Queen Elizabeth Hospital, Woolwich	London - South East Neonatal Network	Dr Olutoyin Banjoko
Queen's Hospital, Romford	London - North East and North Middlesex Neonatal Network	Dr Wilson Lopez

Confidential: For Review Only

Rotherham District General Hospital	North Trent Neonatal Network	Dr Shameel Mattara
Royal Albert Edward Infirmary	Greater Manchester Neonatal Network	Dr Christos Zipitis
Royal Berkshire Hospital	Thames Valley & Wessex Neonatal Networks	Dr Peter De Halpert
Royal Cornwall Hospital	South West Region	Dr Paul Munyard
Royal Derby Hospital	Trent Perinatal Network	Dr John McIntyre
Royal Devon & Exeter Hospital	South West Region	Dr David Bartle
Royal Hampshire County Hospital	Thames Valley & Wessex Neonatal Networks	Dr Katie Yallop
Royal Lancaster Infirmary	Lancashire and South Cumbria Neonatal Network	Dr Joanne Fedee
Royal Oldham Hospital	Greater Manchester Neonatal Network	Dr Natasha Maddock
Royal Shrewsbury Hospital	Staffordshire, Shropshire & Black Country Newborn & Maternity Network	Dr Deshpande
Royal United Hospital	South West Region	Dr Stephen Jones
Russells Hall Hospital	Staffordshire, Shropshire & Black Country Newborn & Maternity Network	Dr Mahadevan
Salisbury District Hospital	Thames Valley & Wessex Neonatal Networks	Dr Nick Brown
Scunthorpe General Hospital	North Trent Neonatal Network	Dr Pauline Adiotomre
Southend Hospital	London - North East and North Middlesex Neonatal Network	Dr Arfa Khan
St Helier Hospital	London - South West Neonatal Network	Dr Salim Yasin
St Mary's Hospital, IOW	Thames Valley & Wessex Neonatal Networks	Dr Sian Butterworth
St Mary's Hospital, London	London - North West Neonatal Network	Dr Sunit Godambe
St Richard's Hospital	Thames Valley & Wessex Neonatal Networks	Dr Nick Brennan
Stepping Hill Hospital	Greater Manchester Neonatal Network	Dr Carrie Heal
Stoke Mandeville Hospital	Thames Valley & Wessex Neonatal Networks	Dr Sanjay Salgia
Tameside General Hospital	Greater Manchester Neonatal Network	Dr Jacqeline Birch
Taunton & Somerset Hospital	South West Region	Dr Chris Knight
Tunbridge Wells Hospital	South East Coast Neonatal ODN	Dr Hamudi Kisat
University Hospital Lewisham	London - South East Neonatal Network	Dr Jauro Kuna
University Hospital of North Durham	Northern Neonatal Network	Dr Mehdi Garbash
University Hospital of South Manchester	Greater Manchester Neonatal Network	Dr Gopi Vemuri
Victoria Hospital, Blackpool	Lancashire and South Cumbria Neonatal Network	Dr Chris Rawlingson
Warrington Hospital	Cheshire and Merseyside Neonatal Network	Dr Delyth Webb
Watford General Hospital	Beds-Herts Neonatal Network	Dr Sankara Narayanan
West Suffolk Hospital	Norfolk, Suffolk & Cambridgeshire Neonatal Network	Dr Ian Evans
Wexham Park Hospital	Thames Valley & Wessex Neonatal Networks	Dr Rekha Sanghavi
Whipps Cross University Hospital	London - North East and North Middlesex Neonatal Network	Dr Caroline Sullivan
Whiston Hospital	Cheshire and Merseyside Neonatal Network	Dr Rosaline Garr
Whittington Hospital	London - North Central Neonatal Network	Dr Wynne Leith
Worcestershire Royal Hospital	Midlands South West Newborn Network	Dr Andrew Gallagher
York District Hospital	Yorkshire Neonatal Network	Dr Guy Millman
Gloucestershire Royal Hospital	South West Region	Dr Simon Pirie
Arrowe Park Hospital	Cheshire and Merseyside Neonatal Network	Dr Anand Kamalanathan
Birmingham Heartlands Hospital	Midlands South West Newborn Network	Dr Phil Simmons
Birmingham Women's Hospital	Midlands South West Newborn Network	Dr Anju Singh
Bradford Royal Infirmary	Yorkshire Neonatal Network	Dr Sunita Seal
Chelsea & Westminster Hospital	London - North West Neonatal Network	Dr Mark Thomas
Derriford Hospital	South West Region	Dr Alex Allwood
Guy's & St Thomas' Hospital	London - South East Neonatal Network	Dr Timothy Watts
Homerton Hospital	London - North East and North Middlesex Neonatal Network	Dr Narendra Aladangady
hull royal infirmary	Yorkshire Neonatal Network	Dr Hassan Gaili
James Cook University Hospital	Northern Neonatal Network	Dr M Lal
King's College Hospital	London - South East Neonatal Network	Dr Ann Hickey
Lancashire Women & Newborn Centre	Lancashire and South Cumbria Neonatal Network	Dr Meera Lama
Leeds Neonatal Service	Yorkshire Neonatal Network	Dr Lawrence Miall
Leicester Neonatal Service	Midlands Central Neonatal Network	Dr Jonathan Cusack

Confidential: For Review Only

Liverpool Women's Hospital	Cheshire and Merseyside Neonatal Network	Dr Bill Yoxall
Luton & Dunstable Hospital	Beds-Herts Neonatal Network	Dr Jennifer Birch
Medway Maritime Hospital	South East Coast Neonatal ODN	Dr Aung Soe
New Cross Hospital	Staffordshire, Shropshire & Black Country Newborn & Maternity Network	Dr Tilly Pillay
Norfolk & Norwich University Hospital	Norfolk, Suffolk & Cambridgeshire Neonatal Network	Dr Mark Dyke
Nottingham City Hospital	Trent Perinatal Network	Dr Steven Wardle
Nottingham University Hospital (QMC)	Trent Perinatal Network	Dr Steven Wardle
Oxford University Hospitals, John Radcliffe Hospital	Thames Valley & Wessex Neonatal Networks	Dr Eleri Adams
Princess Anne Hospital	Thames Valley & Wessex Neonatal Networks	Dr Mike Hall
Queen Alexandra Hospital	Thames Valley & Wessex Neonatal Networks	Dr Charlotte Groves
Queen Charlotte's Hospital	London - North West Neonatal Network	Dr Sunit Godambe
Rosie Maternity Hospital, Addenbrookes	Norfolk, Suffolk & Cambridgeshire Neonatal Network	Dr Angela D'Amore
Royal Bolton Hospital	Greater Manchester Neonatal Network	Dr Paul Settle
Royal Preston Hospital	Lancashire and South Cumbria Neonatal Network	Dr Richa Gupta
Royal Stoke University Hospital	Staffordshire, Shropshire & Black Country Newborn & Maternity Network	Dr Alison Moore
Royal Sussex County Hospital	South East Coast Neonatal ODN	Dr P Amess
Royal Victoria Infirmary	Northern Neonatal Network	Dr Alan Fenton
NORTH BRISTOL NHS TRUST (SOUTHMEAD)	South West Region	Dr Paul Mannix
St George's Hospital	London - South West Neonatal Network	Dr Charlotte Huddy
St Mary's Hospital, Manchester	Greater Manchester Neonatal Network	Dr Ngozi Edi-Osagie
St Michael's Hospital	South West Region	Dr Pamela Cairns
St Peter's Hospital	South East Coast Neonatal ODN	Dr Peter Reynolds
Sunderland Royal Hospital	Northern Neonatal Network	Dr Majd Abu-Harb
The Jessop Wing, Sheffield	North Trent Neonatal Network	Dr Simon Clark
The Royal London Hospital - Constance Green	London - North East and North Middlesex Neonatal Network	Dr Vadivelam Murthy
University College Hospital	London - North Central Neonatal Network	Dr Giles Kendall
University Hospital Coventry	Midlands Central Neonatal Network	Dr Kate Blake
University Hospital of North Tees	Northern Neonatal Network	Dr Hari Kumar
William Harvey Hospital	South East Coast Neonatal ODN	Dr Vimal Vasu

Confidential: For Review Only
